# Evolution and host-specific adaptation of *Pseudomonas aeruginosa*

**DOI:** 10.1126/science.adi0908

**Published:** 2024-07-05

**Authors:** Aaron Weimann, Adam M Dinan, Christopher Ruis, Audrey Bernut, Stéphane Pont, Karen Brown, Judy Ryan, Lúcia Santos, Louise Ellison, Emem Ukor, Arun P. Pandurangan, Sina Krokowski, Tom L. Blundell, Martin Welch, Beth Blane, Kim Judge, Rachel Bousfield, Nicholas Brown, Josephine M. Bryant, Irena Kukavica-Ibrulj, Giordano Rampioni, Livia Leoni, Patrick T. Harrison, Sharon J Peacock, Nicholas R. Thomson, Jeff Gauthier, Jo L Fothergill, Roger C Levesque, Julian Parkhill, R. Andres Floto

**Affiliations:** 1 Victor Phillip Dahdaleh Heart & Lung Research Institute, University of Cambridge, Cambridge UK; 2 University of Cambridge Molecular Immunity Unit, MRC Laboratory of Molecular Biology, Cambridge UK; 3 Cambridge Centre for AI in Medicine, University of Cambridge UK; 4 Department of Veterinary Medicine, University of Cambridge; Cambridge, UK; 5 Laboratory of Pathogens and Host Immunity (LPHI), UMR5235, CNRS/ Université de Montpellier, Montpellier, France; 6 Cambridge Centre for Lung Infection, Royal Papworth Hospital, Cambridge UK; 7 Department of Physiology, Bioscience Institute, University College Cork, Ireland; 8 Department of Biochemistry, University of Cambridge, Cambridge, UK; 9 Department of Medicine, University of Cambridge, Cambridge UK; 10 Cambridge University Hospitals Trust, Cambridge, UK; 11 Wellcome Sanger Institute, Hinxton, UK; 12 Institut de biologie intégrative et des systèmes (IBIS), Université Laval; Québec City, Québec, Canada; 13 Department of Science, University Roma Tre, Rome, Italy; 14 IRCCS Fondazione Santa Lucia, Rome, Italy; 15 London School of Hygiene and Tropical Medicine, london, UK; 16 Department of Clinical Infection, Microbiology and Immunology, University of Liverpool, Liverpool UK

## Abstract

The major human bacterial pathogen, *Pseudomonas aeruginosa*, causes multidrug-resistant infections in people with underlying immunodeficiencies or structural lung diseases, such as Cystic Fibrosis (CF). We show that a few environmental isolates, driven by horizontal gene acquisition, have become dominant epidemic clones that have sequentially emerged and spread through global transmission networks over the past 200 years. These clones demonstrate varying intrinsic propensities for infecting CF or non-CF individuals (linked to specific transcriptional changes enabling survival within macrophages), have undergone multiple rounds of convergent, host-specific adaptation, and have eventually lost their ability to transmit between different patient groups. Our findings thus explain the pathogenic evolution of *P. aeruginosa* and highlight the importance of global surveillance and cross-infection prevention in averting the emergence of future epidemic clones.

*P. aeruginosa* is found widely in natural and man-made environments (1–5) and has become an increasingly important opportunistic human pathogen, causing acute nosocomial lung, soft tissue, and systemic infections (6), as well as chronic pulmonary infections in individuals with underlying inflammatory lung diseases, such as Chronic Obstructive Pulmonary Disease (COPD) (7), CF (8), and non-CF bronchiectasis (9), where it causes increased morbidity and mortality (8, 10). Antimicrobial resistance (AMR) in *P. aeruginosa* is increasing globally (recognised by its inclusion in the WHO ESKAPE pathogen list (11)) and is responsible for over 300,000 deaths annually (12).

Although individuals frequently acquire *P. aeruginosa* independently from the environment, hospital-based person-to-person transmission is well recognised in people with CF, leading to strict cohort and individual segregation within clinics (8), but has not been considered a risk in other patient cohorts (13). Nevertheless, epidemic clones of *P. aeruginosa* have been identified in both CF and non-CF infection studies (14–17), suggesting the presence of wide-spread transmission networks. In CF, these epidemic clones (such as the Liverpool Epidemic Strain; LES) are associated with accelerated inflammatory lung damage and worse clinical outcomes, demonstrating that epidemic clones can emerge with increased virulence in particular patient groups.

We therefore sought to understand the pathogenic evolution of *P. aeruginosa*, by defining when and how epidemic clones emerged and spread, exploring how they have adapted to specific hosts, and understanding how within-host evolution has influenced ongoing transmission patterns.

## Results

### Phylogenetic analysis reveals global epidemic clones

**Figure 1:**
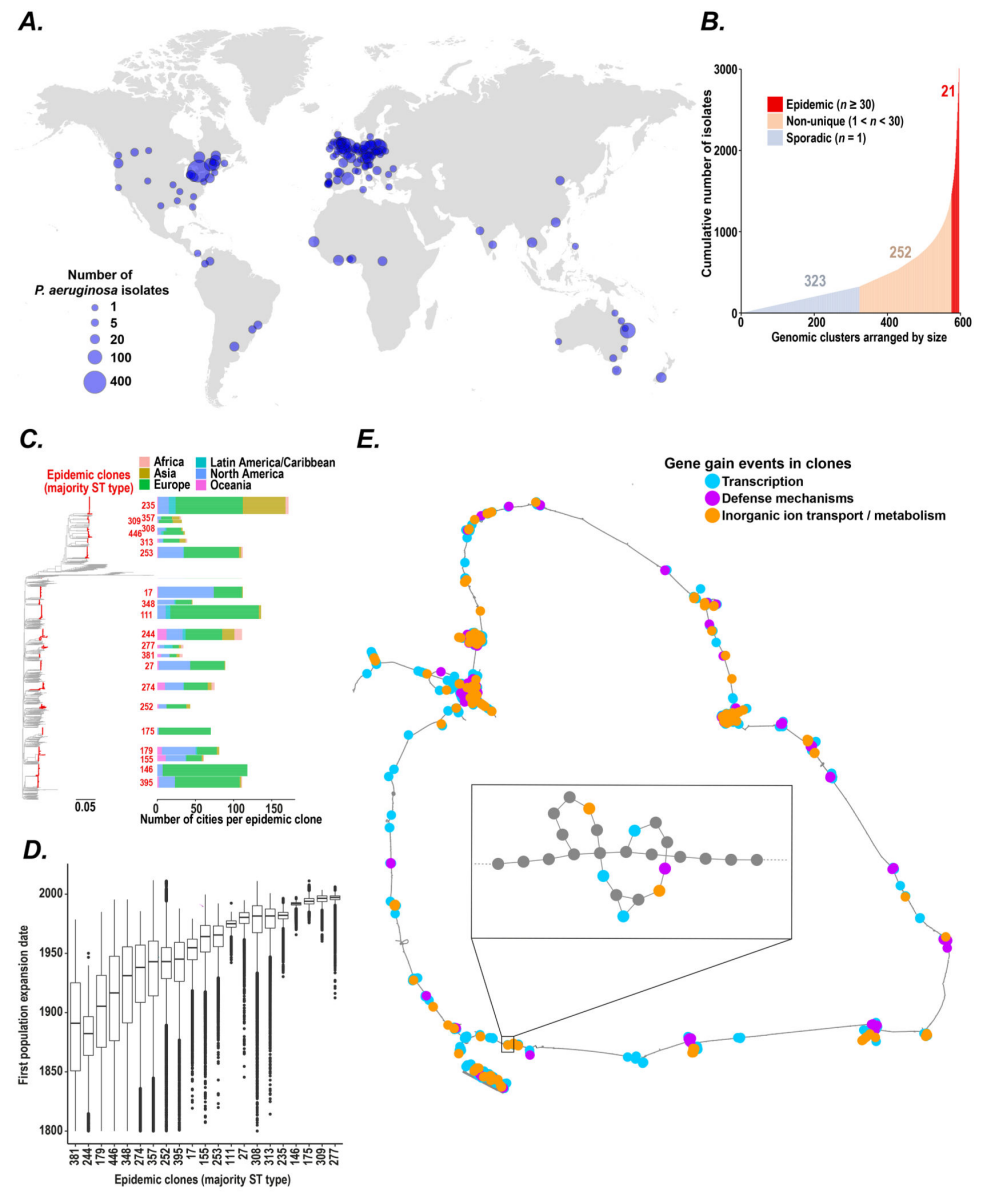
The emergence of epidemic clones of *Pseudomonas aeruginosa*. (**A**) Geographical location of the whole genome sequenced *P. aeruginosa* isolates obtained from patients, animals, and environment analysed in this study (*n =* 9,573). Number of samples from each location indicated by the size of blue dot. (**B**) Cumulative number of isolates across *P. aeruginosa* clones (defined by clustering genomes using the unweighted pair group method with arithmetic means; see Supplementary Methods), arranged by ascending number of genomes per clone and stratified into epidemic (*n* ≥30 isolates/clone; *red*), non-unique (1 < *n* < 30 isolates/clone; *light brown*), and unique (*n =* 1 isolate/clone; *blue*) groups. (**C**) *Left:* Maximum likelihood phylogenetic tree generated from genomes of all study isolates (major epidemic clones labelled in red). *Right* Bar plot representing the number of cities where each epidemic clone was found, coloured by continent. (**D**) Estimated date of first population expansion of 21 epidemic clones (predicted by Bayesian inference using *BEAST* (29)) with graph showing median and interquartile range (IQR; *boxplots*), 1.5 times IQR range (*whiskers*), and data points outside this range (*black points*). (**E**) Pangenome graph analysis of ancestral representatives of epidemic clones (*n* = 21) and sporadic clones (*n* = 80), constructed using *Panaroo* (39), where nodes represent clusters of orthologous genes and two nodes are connected by an edge if they are adjacent on a contig in any sample from the population, define gene gain events associated with the emergence of epidemic clones (described in detail in Figure S5) with genes highlighted that are involved in transcription (*blue*), defense mechanisms (*purple*), and inorganic ion transport and metabolism (*yellow*).For illustration purposes, the graph has been ordered against the genome of *P. aeruginosa* PAO1. *Inset:* magnified section of the pangenome graph is shown to illustrate node and edge structure.

We first analysed a globally-distributed collection of 9,829 human, animal, and environmental isolates of *P. aeruginosa*, including 9,573 human clinical samples from 2,765 patients (14, 18–27) (Figure 1A; Table S1), which we grouped into 596 genetically-related clones (based on pairwise single nucleotide polymorphism (SNP) distances) and then stratified by the number of patients infected by each clone (Figure 1B; Figure S1; Supplementary Methods). We identified 21 major clones each containing isolates from at least 30 individuals, which we term ‘epidemic’ and refer to by their majority multi-locus sequence type (28).

We found that these epidemic clones were more likely to be detected in infected humans than in the non-built environment (Fisher exact test *p* = 7.80 x 10^-12^), caused 51 % of all clinical *P. aeruginosa* infections worldwide, were widely distributed across the phylogenetic tree, and had all spread globally (Figure 1C; Figure S1; Table S1).

### Inferring population expansion and geography of epidemic clones over time

Since our sample collections spanned over 100 years (from 1900 to 2018), we wondered whether we could explore the historical origins of epidemic clones using Bayesian temporal reconstruction (29). We estimate that epidemic clones emerged non-synchronously between the late 17^th^ and late 20^th^ centuries (Figure S2) and (through Skyline demographic modelling (30); Figure S3) have each undergone at least one major population expansion between 1850 and 2000 (Figure 1D), suggesting (considering only extant clones) that *P. aeruginosa* has undergone relatively recent changes in host-pathogen dynamics, potentially driven by changes in human population density, migration patterns (31, 32), and/or increased susceptibility to infections (caused, for example, by increased air pollution during industrialisation (33–35)).

For epidemic clones with geographically clustered clades (association test *p* value <0.05), we implemented Bayesian phylogeographic methods (36) to infer the ancestral location of clones (accepting the limitations of our opportunistic sample collection). In some instances, for example ST235, we were able to find statistical support for the direction of intercontinental spread (from South America to North America and Europe, and then subsequently Asia and Africa; Figure S4), whereas, for other clones such as ST17 and ST27, we could identify only that transmission was restricted to between Europe and North America and peaked in the second half of the 20^th^ century (Figure S4). We conclude that epidemic clones have likely arisen from ancestral locations distributed around the world.

### Horizontal gene transfer may drive emergence of epidemic clones

We next asked why some *P. aeruginosa* clones had become epidemic and investigated whether gene acquisition, through horizontal transfer, might have driven large jumps in human infectivity through saltatory evolution (37) (meaning abrupt changes in evolutionary fitness caused by sudden large genetic changes), as previously observed in *Mycobacterium abscessus* (38). To accurately analyse the *P. aeruginosa* accessory genome, we first inferred a pan-genome graph (using *Panaroo* (39)), with nodes as clusters of orthologous genes and two nodes linked by an edge if they were found adjacent in any contig (Figure 1E). We then compared the accessory genomes of representatives of ancestral epidemic clones with those of sporadic isolates and found that epidemic clones had significant enrichment of genes involved in transcriptional regulation, inorganic ion transport, lipid metabolism, and protein turnover, with significant depletion of genes involved in bacterial defence and secretion (Fisher exact test, FDR = 0.1; Figure 1E; Figure S5, Table S2); suggesting that fundamental changes in bacterial physiology might have driven the development of epidemic clones.

### Epidemic clones vary in their intrinsic host preference

**Figure 2:**
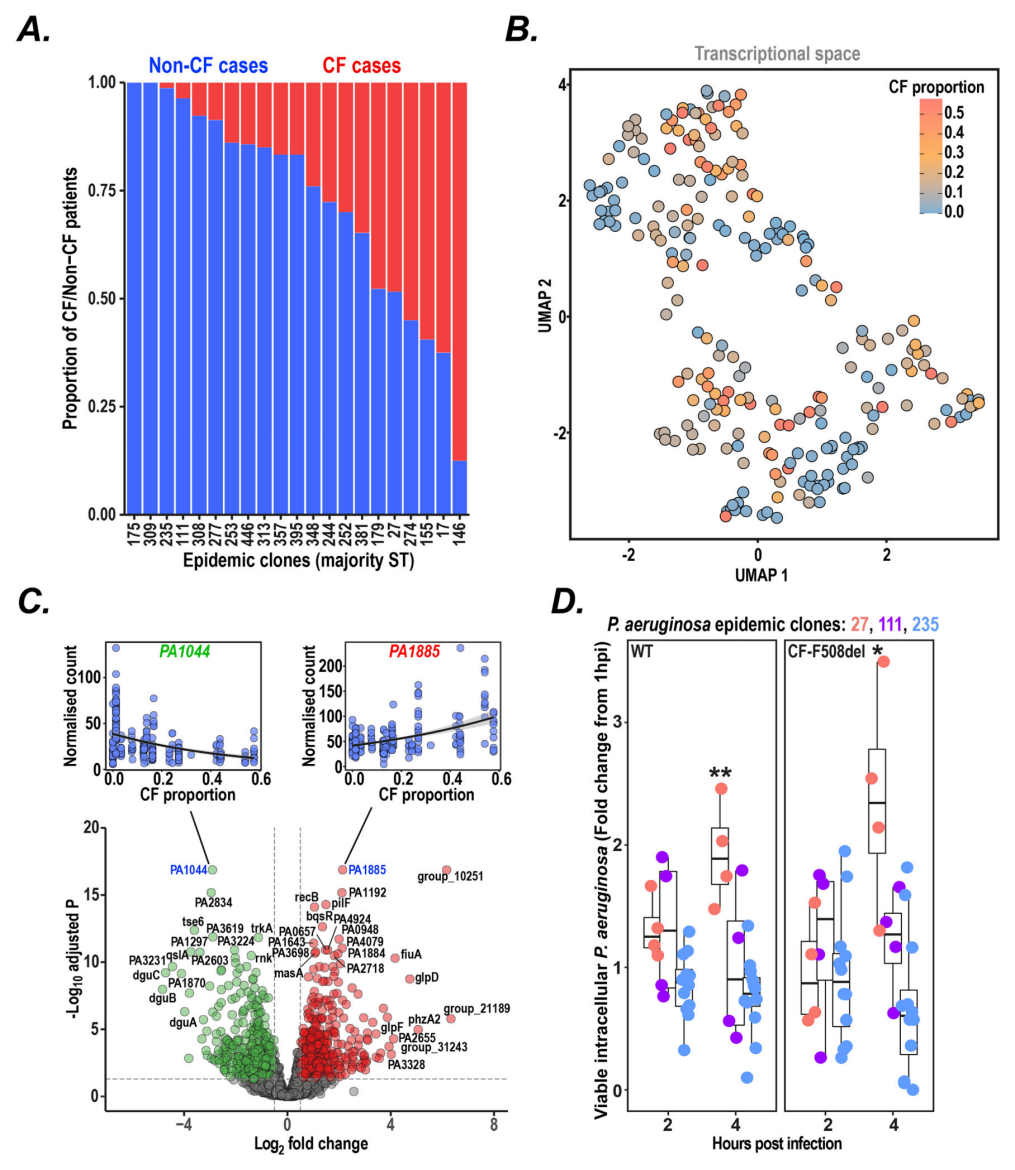
Variable intrinsic host preference of epidemic *P. aeruginosa* clones. (**A**) Proportion of infections caused by epidemic clones (labelled by their majority multi-locus sequence type, ST) in cystic fibrosis (CF; *red*) and non-CF (*blue*) patients. (**B**) UMAP projection of transcriptomes from representative isolates of epidemic clones (25), colour-coded by the CF affinity of each clone. Expression data were pseudo-aligned to strain-specific gene indices to produce estimates of gene transcript abundance. (**C**) Transcriptome-wide association of gene expression with CF affinity. Transcript abundances were modelled as a response to the proportion of CF infections caused by each epidemic clone using a negative binomial generalised linear model. Volcano plot visualization of the Log2-fold expression change with CF proportion for every gene in the 99% core genome of *Pseudomonas aeruginosa* (center). Genes with an adjusted p-value of less than 0.05 and a log2 fold change less than -0.5 were coloured in green, genes with a log2 fold change greater than 0.5 were coloured in red. The coefficients for gene models were assessed using the Wald test (FDR = 0.05). Normalized expression counts vs CF proportions per epidemic clone with a trendline for the two genes with the lowest and highest log2 fold change, respectively, are shown above (top left/top right). Bulk RNA seq data was analysed from 241 clinical isolates of epidemic clones (25) included in our strain collection. (**D**) Survival of epidemic clones within wildtype (WT) or isogenic F508del knock-in THP1 macrophages at 2 and 4h post infection, expressed as fold change from 1 hour post infection showing median and interquartile range (IQR; *boxplots*), 1.5 times IQR range (*whiskers*. Experiments (carried out at least in duplicate) were performed by exposing THP1 macrophages to pooled isolates of 51 clinical isolates at a multiplicity of infection (MOI) of less than 1. Viable bacteria were isolated from macrophages at time points indicated and grown on solid media. Isolate abundance was quantified using sequence-based deconvolution. Strains with less than 1% abundance at the 1h time point were excluded from the analysis. A difference in the abundance of ST27 strains vs ST111 and ST235 strains at the 4h timepoint was assessed using a two-tailed t-test. * p-value < 0.05, ** p-value < 0.01.

We next examined the types of human infection caused by the epidemic clones and found that ST146, also known as the Liverpool Epidemic Strain, caused infection almost exclusively in people with CF while other clones (e.g., ST175 and ST309) caused infection only in non-CF individuals, with a range of CF and non-CF host distributions for other clones (Figure 2A). While our collection was opportunistically sampled, our findings were replicated when re-analysing existing systematic UK surveillance data (40) for the frequency of CF and non-CF lung infections caused by each epidemic clone (Figure S6). We hypothesised that this apparent clone-specific host preference might relate to intrinsic differences in bacterial behaviour between clones. We found no evidence, on pangenome analysis, for an association of host affinity with acquisition of genes with a particular function (Figure S7) and therefore we reasoned that changes in gene expression could explain differences in host preference. We therefore analysed a previous transcriptomic study of clinical *P. aeruginosa* isolates (nearly all from non-CF patients; (25)) that were represented in our sample collection, and found that clinical isolates clustered in transcriptional space based on the host-preference of epidemic clones (*p <* 0.001; Figure 2B; Supplementary Methods).

We next explored whether there were any patterns in gene expression associated with clone predisposition for specific human hosts and identified, using a negative binomial generalised linear model, a clear expression signature of 624 genes positively associated, and 514 genes inversely associated, with affinity for causing CF infection (Wald test, FDR = 0.05; Figure 2C; Table S3).

### Increased survival within macrophages of high CF affinity clones

To identify a potential mechanism by which differential gene expression could alter the host-preference of epidemic clones, we conducted a multi-dimensional phenotypic characterisation of 49 representative isolates (almost all from non-CF patients to minimise the contribution of secondary host-specific adaptation) from epidemic clones with low (ST235, ST111), intermediate (ST253), and high levels of CF affinity (ST17, ST27, ST146) obtained from the International Pseudomonas Consortium Database (21). We initially examined isolate behaviour in established assays of *Pseudomonas* virulence (biofilm formation, siderophore production, swim and twitch motility, and production of caseinase and gelatinase) but could find no correlation with clonal host affinity (Figure S8).

We therefore decided to test the ability of representative isolates of different epidemic clones to withstand intracellular killing by macrophages that, together with neutrophils, are thought to be the first line of defence against bacterial lung infection (41, 42). We found significantly increased intracellular survival and replication of isolates from the high CF affinity clone (ST27) compared to isolates from the low/intermediate clones ST111 and ST235 in both wildtype and CF (F508del homozygous) isogenic macrophage cell lines (Figure 2D; Table S4), suggesting that enhanced host innate immune evasion might explain the intrinsic success of particular epidemic clones in infecting CF patients.

### Host preference of epidemic strains is mediated by DksA1 expression

**Figure 3:**
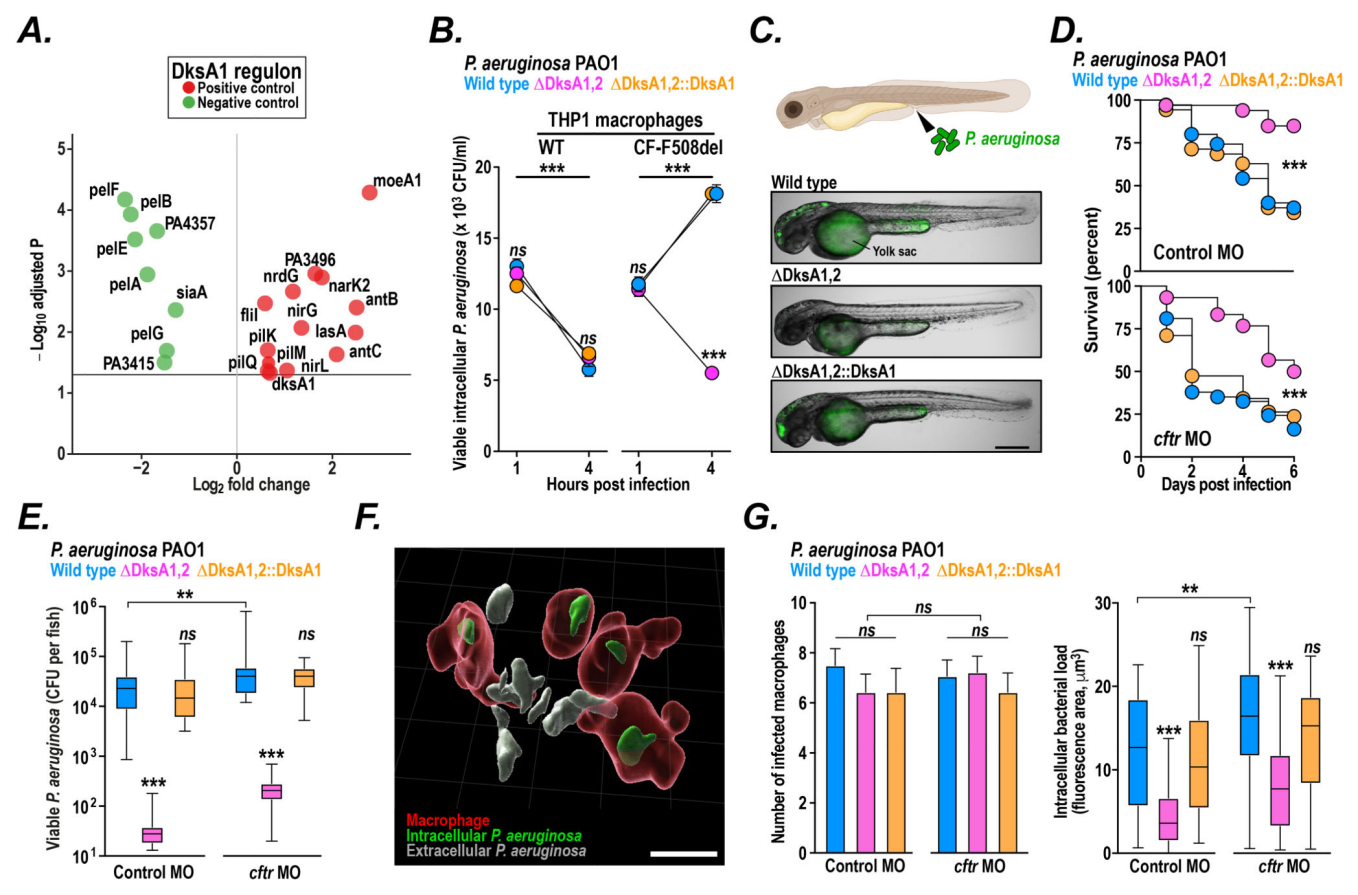
Activation of the DksA1 regulon contributes to Cystic Fibrosis host preference of *P. aeruginosa* clones. **(A)** Volcano plot visualisation of the Log2-fold expression change with CF proportion for genes positively controlled (*red*) and negatively controlled (*green*) within the DskA1 regulon as defined by Fortuna et al. (44). Bulk RNA seq data was analysed from 241 clinical isolates of epidemic clones *(24)* included in our strain collection. (**B**) DksA1 promotes survival of *P. aeruginosa* within CF macrophages. Viable intracellular *P. aeruginosa* (quantified through enumeration of cell-associated colony forming units; CFU) were measured at 1h and 4h post infection of differentiated wildtype (WT) and isogenic F508del homozygous knockin (CF-F508del) THP1 cells with wildtype (*blue*), isogenic DskA1-DskA2 double knockout (ΔDksA1,2; *pink*), and knockout complemented with DksA1 ((ΔDksA1,2::DksA1; *yellow*) *P. aeruginosa* PAO1. Data (mean ± SEM) are representative of at least three independent experiments performed in at least triplicate. *** p < 0.001; *ns* not significant (two-tailed Student’s t-test). (**C**) (B) *Top*: Cartoon of zebrafish (created with *BioRender.com*) illustrating injection site for GFP-labelled fluorescent *P. aeruginosa. Bottom*: Representative fluorescence and DIC images of whole infected zebrafish larvae at 1 day post-infection (Scale bar: 150 μm; the labelled yolk sac is autofluorescent). (**D**) Survival analysis of control (*top*) and *cftr* morphant (cftr MO; *bottom*) zebrafish larvae infected intravenously (250-350 CFU) with *P. aeruginosa* PAO1 wildtype (*blue*), ΔDksA1,2 knockout (*pink*), and ΔDksA1,2::DksA1 complemented (*yellow*) fluorescent strains plotted as the percentage of surviving animals over 6 days (average of 2 independent experiments; n = 30-38 fish for each condition); *** *p* < 0.001 (Mantel-Cox Log-rank test). (**E**) Viable *P. aeruginosa* in zebrafish larvae at Day 1 post infection with *P. aeruginosa* PAO1 wildtype (*blue*), ΔDksA1,2 knockout (*pink*), and ΔDksA1,2::DksA1 complemented (*yellow*) fluorescent strains (plotted as mean ± IQR colony forming units (CFU) per fish of at least 3 independent experiments; n = 15-20 larvae per condition. *** *p* < 0.001; *ns* not significant (two-way ANOVA with Tukey’s post-test). (**F**,**G**) Control and *cftr* morphant zebrafish larvae with mCherry-labelled macrophages (*Tg(mpeg1:mcherry-F)ump2 (45)*) were intramuscularly infected with 250-350 GFP-labelled *P. aeruginosa* PAO1 wildtype, ΔDksA1,2 or ΔDksA1,2::DksA1 strains) and the infection tracked using real-time intravital confocal microscopy. (**F**) Representative 3D reconstruction of confocal imaging showing macrophages (*red*) and automatic classification of extracellular (*grey*) and intracellular (*green*) *P. aeruginosa* (Scale bar 10 μm). (**G**) Quantification of the number of infected macrophages at the site of injection (*left*) and the level of intracellular bacterial load (calculated by the volume of bacteria-associated fluorescence observed within each macrophage) at 6 hours post infection with *P. aeruginosa* PAO1 wildtype (*blue*), ΔDksA1,2 knockout (*pink*), and ΔDksA1,2::DksA1 complemented (*yellow*) fluorescent strains. Mean ± IQR of at least 54 cells per condition (from *n =* 4-6 larvae) recorded from 2 independent experiments. ** *p* < 0.01; *** *p <* 0.001; *ns* not significant (two-way ANOVA with Tukey’s post-test).

To further explore the bacterial mechanisms contributing to enhanced intracellular survival of bacterial isolates from high CF affinity clones, we interrogated the differentially-expressed gene set (Figure 2C) and discovered that both the expression of the stringent response modulator DksA1 and the activation of its regulon were associated with CF affinity (Figure 3A). Since DksA1 had previously been implicated in enhancing *P. aeruginosa* survival within mouse macrophages and increasing tolerance to H_2_O_2_ (43), we examined the impact of deleting DksA1 on bacterial survival by using *in vitro* and *in vivo* models of non-CF and CF infection employing *P. aeruginosa* PAO1 wildtype and isogenic DksA1 and DksA2 double knockout (ΔDksA1,2), and complemented (ΔDksA1,2::DksA1) strains (ΔDksA1,2 double knockout mutants were used due to gene redundancy (44).

We first tested bacterial survival in macrophages and found that, while all three strains were effectively killed in wildtype THP1 macrophages, only the ΔDksA1,2 strain could be killed by isogenic CF (F508del knock-in) cells while wildtype and complemented bacteria were able to resist macrophage killing and replicate intracellularly (Figure 3B). Our findings reveal a number of important features of host-*Pseudomonas* interactions: firstly, that there are intrinsic defects in CF macrophages that facilitate intracellular survival of *P. aeruginosa* (observations that are supported by previous *in vitro* reports (45) and by our *in vivo* experiments in zebrafish, where deletion (46) or morpholino knockdown of the Cystic Fibrosis Transmembrane Regulator (*cftr*) compromises survival after intravenous infection (Figure S9)); secondly, that intracellular survival in CF macrophages is mediated by DskA1, raising intriguing mechanistic questions about the role of the stringent response in surviving the phagosomal environment; and finally, that differences in DksA1 expression across epidemic clones may explain their observed different abilities to survive within macrophages and, potentially as a consequence, their varying intrinsic host preferences.

To explore the potential role of DksA1 further, we examined the behaviour of fluorescently labeled wildtype, ΔDksA1,2 mutant and complemented *P. aeruginosa* PAO1 during *in vivo* infection in zebrafish larvae (Figure 3C-G). We observed increased survival of both control and *cftr* morpholino-treated fish after infection with ΔDksA1,2 bacteria compared to wildtype and complemented strains (Figure 3D); findings which correlated with an observed decreased survival of ΔDksA1,2 bacteria *in vivo* (Figure 3E).

We then exploited the optical transparency of zebrafish larvae to track the interaction of macrophages with *P. aeruginosa* following intramuscular infection using intravital confocal microscopy. Utilizing a fluorescent macrophage reporter fish line (*Tg(mpeg1:mcherry-F)ump2*; (47)), we could clearly identify macrophages and distinguish extracellular from intracellular fluorescent bacteria (Figure 3F). We found no difference in the mobilization of macrophage to the site of infection, or the proportion of infected macrophages, in control and *cftr* morpholino fish infected with wildtype, ΔDksA1,2 mutant or complemented *P. aeruginosa* PAO1 bacteria (Figure S9; Figure 3G). We did however observe a clear reduction in macrophage bacterial burden (suggesting reduced intracellular replication) following ΔDksA1,2 infection of both control and *cftr* morpholino fish lines (Figure 3G), confirming the critical role of DksA1 for intracellular survival during *in vivo P. aeruginosa* infection.

Taken together, our data indicate that intrinsic elevations in DksA1 expression levels in some epidemic clones may have enabled them to exploit potential innate immune defects in CF and adopt the specific evolutionary strategy of replicating within macrophages.

### Convergent host-specific adaptation of *P. aeruginosa*

We next examined how, once selected from the environment, epidemic clones of *P. aeruginosa* have adapted to the human host through multiple rounds of within-patient evolution by analysing the recent mutation history of individual clones.

**Figure 4:**
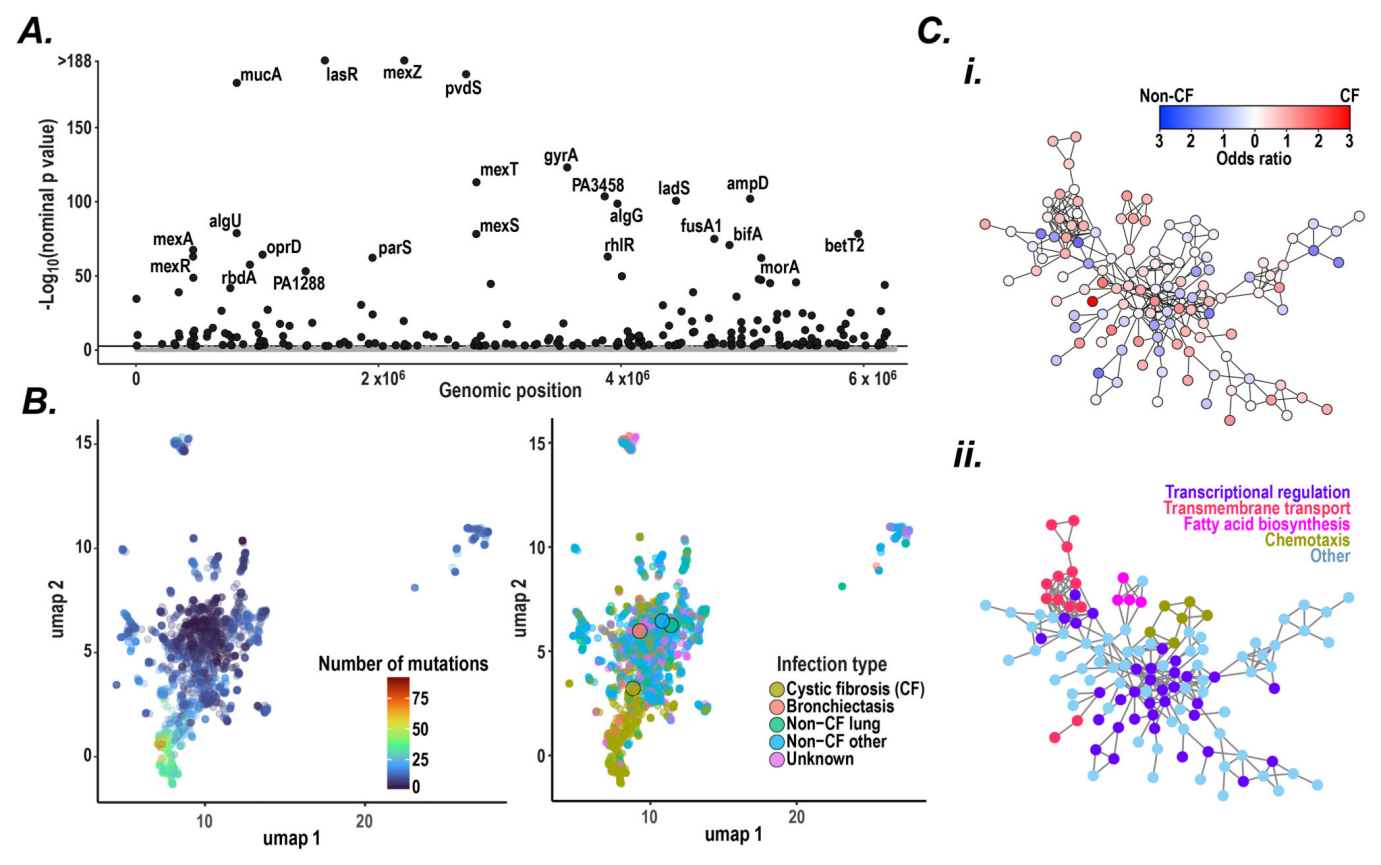
Host-specific pathoadaptation of *P. aeruginosa* (**A**) Manhattan plot showing nominal *p* values (plotted as -Log10) from genome-wide mutational burden test across all genes in *P. aeruginosa* PAO1. Significance was assessed using a Poisson test comparing the expected and observed number of mutations in each gene accounting for the proportion of genomes that gene was found in the pan-genome (FDR = 0.1; genes with a significant mutational burden, termed pathoadaptive, shown in *black*, others in *grey*). (**B**) UMAP projections of host adaptation of isolates (based on acquired mutations in pathoadaptive genes) colour-coded by (*left*) number of pathoadaptive mutations and (*right*) type of infection (centroids denoted by larger dots). Isolates without any pathoadaptive mutations were removed from the analysis. (**C**) Protein-protein interaction network for the pathoadaptive genes extracted from the STRING database (only main connected component shown, full graphs shown in Fig. S12; (56)). Genes are shown as nodes which are connected by an edge if they had an interaction reported in STRING (confidence > 0.7). *Top:* To estimate host-specific pathoadaption, the number of cystic fibrosis (CF) vs non-CF mutations (determined by stratifying mutations in pathoadaptive genes on terminal branches by the infection type of isolates) were compared using a Fisher exact test (FDR = 0.1) and expressed as an odds ratio for each gene. *Bottom:* Gene nodes were colour-coded by class of functional annotation (based on overrepresented pathways using Gene Ontology (89) biological process enrichment analysis with TopGO (57) among CF: transmembrane transport and fatty acid biosynthesis, and non-CF: transcriptional regulation and chemotaxis).

By reconstructing mutations that had likely occurred since the emergence of each clone, we found strong evidence for convergent molecular evolution, identifying 224 out of 5641 genes that had a higher total mutational burden than expected by chance (Poisson test, FDR = 0.05; Figure 4A; Table S5), which we term ‘pathoadaptive’. Mutations in these pathoadaptive genes were more likely to be non-synonymous and deleterious (by variant effect annotation (48)) than those found in other genes (Fisher exact test *p* < 1.0 x 10^-16^), and were predicted to be more likely to cause protein dysfunction, as estimated by both sequence conservation methods (*SIFT* (49); Wilcoxon rank-sum test, *p* = 9.04 x 10^-15^) and structural modelling approaches (*FoldX* (50); Wilcoxon rank-sum test, *p* = 1.34 x 10^-6^) (Figure S10), suggesting that pathoadaptation is largely driven by loss-of-function mutations. We explored the functional impact of pathoadaptive mutations experimentally by using existing RNAseq datasets (25) to examine the effect of transcription factor variants on expression of their previously characterised regulons (51–54) and found that clinical isolates with pathoadaptive variants in several transcription factor had statistically significant shifts in regulon expression levels compared to controls (two tailed t-test with adjusted p-value < 0.00014; Figure S10), supporting the concept of a general loss-of-function evolutionary process driving *P. aeruginosa* pathoadaptation.

We were able to functionally annotate the majority of these pathoadaptive genes using prior published information (55), identifying many of them as having established roles in recognised pathogenic processes including biofilm formation, antibiotic resistance and LPS modification (Figure S11). The number of genes with an established function was much higher among pathoadaptive genes than in other genes (Fisher exact test *p* < 1.0 x 10^-16^; Figure S11), potentially reflecting their central role in *P. aeruginosa* pathobiology. We also characterised the function of 41 pathoadaptive genes experimentally by *de novo* screening relevant transposon mutants in a series of functional assays to quantify virulence traits (Supplementary Methods; Figure S11).

We next examined the nature of host adaptation achieved by individual *P. aeruginosa* isolates by using the profile of their pathoadaptive gene mutations to map them in evolutionary space (defined by the presence or absence of mutations in the 224 pathoadaptive genes). We found that CF isolates clustered separately from others and had accumulated more mutations, suggesting that the CF lung is a distinct niche with different selective pressures compared to other lung or non-lung environments (Figure 4B).

We found that the products of these pathoadaptive genes were tightly interconnected, with more protein-protein interactions than expected by chance (*STRING* database (56); *p* < 1 x 10^-16^; Figure 4C; Figure S12), indicating their likely coordinated functional roles. We observed that 70 genes were more frequently mutated in CF isolates while 55 genes were more commonly mutated in non-CF isolates (Fisher exact test, FDR = 0.1). Among genes that were more commonly mutated in CF or non-CF, we found several overrepresented pathways (using Gene Ontology biological pathway enrichment analysis with TopGO (57)), suggesting that distinct functional programmes were being modified as part of host-specific adaptation (Figure 4C). For example, CF isolates were more likely to have mutated AlgU, a key regulator of mucoidy (58) (with mutations occurring predominantly at the interface between this sigma-H factor and its negative regulator protein, MucA), and PcnA, a putative nicotinamidase (with mutations found within the protein core or at sites of protein-protein interaction; Figure S13). In contrast, non-CF isolates were more likely to have mutated LadS, a calcium-responsive histidine kinase (59) (with mutations concentrated in the N-terminal (sensor) and transmembrane domains), and in the putative choline transporter BetT2 (60), with helix-breaking mutations found within the transmembrane domain (Figure S13).

### Distinct evolutionary trajectories lead to host specialisation

**Figure 5:**
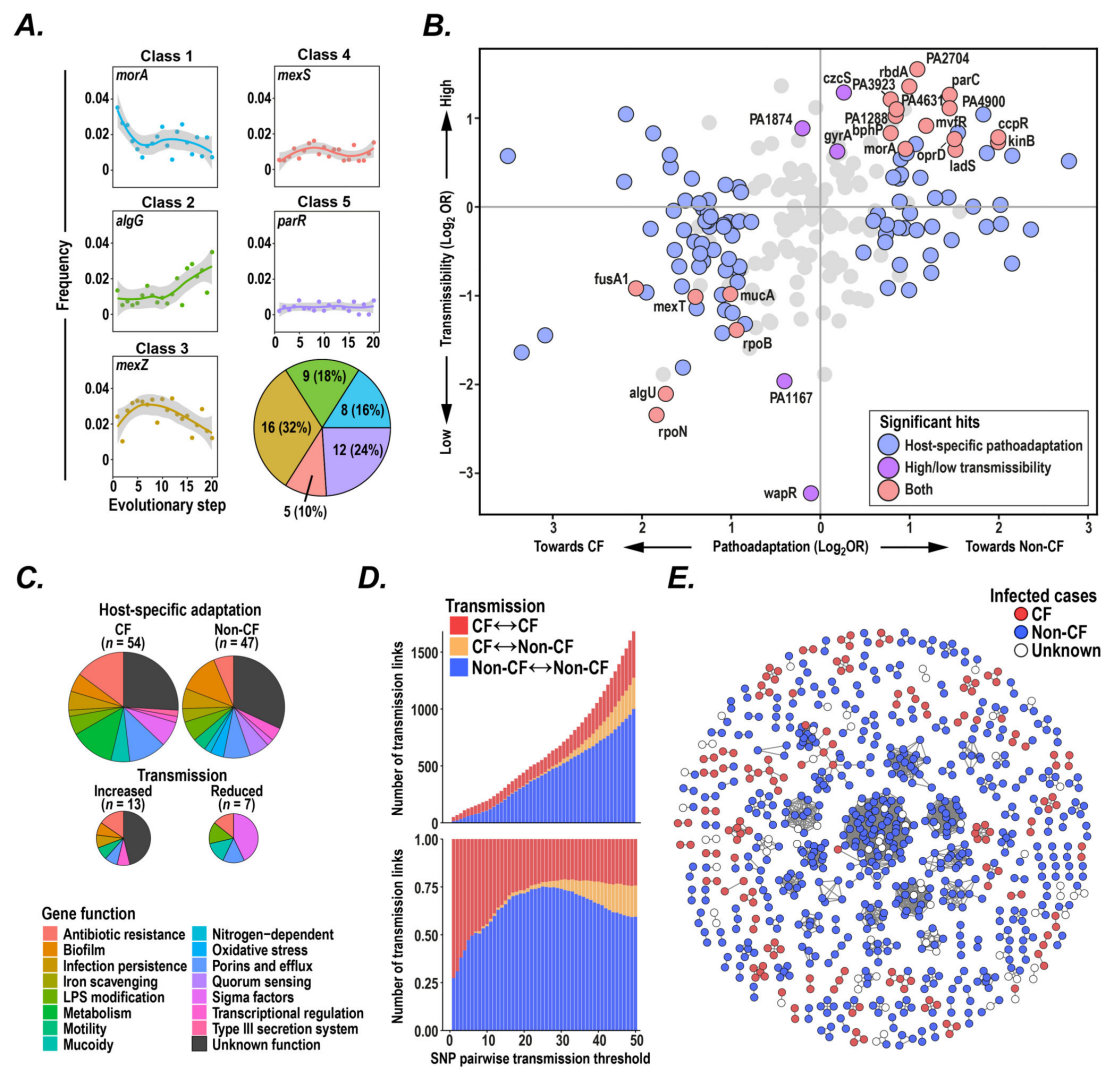
Evolutionary trajectories of *P. aeruginosa* during pathoadaptation. (**A**) Normalised frequency of mutations over evolutionary time in specific pathoadaptive genes. The trajectories of the 50 most commonly mutated genes were manually assigned to one of 5 classes (Figure S15), based on the shape of their mutation frequency curves (relative size of each class and representative examples (with trendlines from locally-weighted smoothing) shown). (**B**) The relative transmissibility and host-specific adaptation of pathoadaptive genes was calculated. To estimate host-specific pathoadaptation, the number of cystic fibrosis (CF) vs non-CF mutations (determined by stratifying mutations in pathoadaptive genes on terminal branches by the infection type of isolates) were compared using a Fisher exact test (FDR = 0.1) and expressed as an odds ratio. To assess the transmissibility of pathoadaptive changes, the number of mutations that had been observed in at least two isolates were compared with mutations that had only been observed once using a Fisher exact test (FDR = 0.1). Genes were colour-coded if showing significant host-specific adaptation (*blue*), changes in transmissibility (*purple*), or both (*pink*). Genes with zero or infinite odds ratio not shown. (**C**) Functional annotation of pathoadaptive genes associated in (*top*) host-specific adaptation and (*bottom*) changes in transmissibility. (**D**) The number (*top*) and proportion (*bottom*) of transmission links across a range of pairwise SNP thresholds occurring between CF to CF (*red*), CF to non-CF (*yellow*), and non-CF to non-CF (*blue*) individuals (data were down-sized to contain equal numbers of CF and non-CF infections). (**E**) Transmission clusters involving patients with CF (*red*), non-CF (*blue*), or unknown status (*white*). Nodes representing isolates were connected by edges if pairwise SNP distances were 26 SNPs or less. This cut-off represents the 95th percentile of the within-host genetic diversity analysed in 81 patients.

We then used ancestral state reconstruction to determine the order of acquisition of each pathoadaptive mutation and thereby recreate the evolutionary trajectory of each isolate. We found that, on average, CF isolates had longer trajectories than non-CF isolates (with 20.5 compared to 11.2 steps, Wilcoxon signed-rank test p < 1.0 x 10^-16^, Figure S14). By looking at the frequency of mutations in pathoadaptive genes at each evolutionary time-step, we were able to cluster genes into 5 groups with distinct temporal signatures (Figure 5A; Figure S15), suggesting that mutations in specific genes may be important at different stages of evolution (as noted previously, specifically for AMR evolution (61)).

We wondered therefore whether specific sets of pathoadaptive genes might be driving distinct evolutionary processes such as host-specific adaptation, person-to-person transmission, or both. To examine this, we inferred the impact of each pathoadaptive gene on host-specific pathoadaptation (by examining the relative frequency of gene mutations occurring in CF compared to non-CF lung isolates), and on bacterial transmissibility (based on the frequency of specific gene mutations being found in isolates from at least two patients) to create a map of the contribution of each pathoadaptive gene to each of these evolutionary processes (Figure 5B), annotating each gene by previously known or experimentally derived function, or the type of temporal mutation signature observed (Figure 5C; Figure S16).

We found that, while some pathoadaptive genes were associated with changes in either transmissibility or host-specific pathoadaptation, many were implicated in both processes (Figure S16). For example, mutations in several genes (such as *mvfR* and *morA*) occurred early in the evolutionary trajectories of isolates and were associated with both adaptation to the non-CF host and increased transmissibility, while mutations in other genes (such as in *fusA1* and *algU*) occurred late in evolutionary journeys and were associated with adaptation to the CF host and decreased transmissibility (Figure S16).

Since we observed a likely deleterious impact of several pathoadaptive mutations on transmissibility, we examined whether pathoadaptation might lead to host specialisation and result in reduced transmission of isolates between CF and non-CF individuals. To explore this possibility, we used the genomic relatedness of isolates to plot the number (and proportion) of transmission links over a range of SNP pairwise thresholds (representing transmission chains of various lengths) and found strong evidence for CF-to-CF patient transmission and non-CF to non-CF patient cross-infection but very little CF to non-CF transmission (Figure 5D). Additionally, we reconstructed transmission clusters at a specific SNP threshold (26 SNPs), based on the measured genetic diversity within individual patients (Supplementary Methods), and found transmission clusters of variable sizes but very few containing both CF and non-CF patients (Figure 5E). It seems likely therefore that host-specific pathoadaptation of epidemic *P. aeruginosa* clades limits transmission between different hosts.

## Discussion

Our findings describe the key sequential steps involved in the evolution of *P. aeruginosa* from an environmental organism to a major human pathogen. We identify horizontal gene acquisition as a likely driver for the emergence of epidemic clones from the environment through saltational evolution (as previously described for *M. abscessus* (38)) and infer their spatio-temporal spread which suggests an increasing rate of new epidemic clone expansions over time (accepting that only extant clones are considered). We identify an intrinsic and variable host-specific affinity across epidemic clones with CF preference potentially causally associated with improved intracellular survival in macrophages. We then describe how deleterious mutations in a discrete set of functionally interrelated genes likely mediate further host specialisation (through multiple rounds of within-patient adaptation) and onward transmission, thereby plausibly explain the observed lack of person-to-person transmission between CF and non-CF patients.

Our work highlights the importance of preventing pathogenic evolution by minimising cross-infection, not just within CF cohorts (where infection control measures are well established) but also between non-CF patients, and emphasises how global surveillance and targeted monitoring of high-risk patient groups will be needed to detect expansion, pathoadaptation, and transmission of new and extant epidemic *P. aeruginosa* clones.

## Methods summary

### Genomic datasets and clone assignment

We collated Pseudomonas aeruginosa genome datasets from studies of antibiotic resistance (18, 23–25), from individuals with cystic fibrosis (14, 26) and non-CF bronchiectasis (22); from the International *Pseudomonas* Consortium (21); and from studies targeting high-risk clones (19, 20, 27). Newly sequenced genomes from the TeleCF study, which involved adults with CF (n = 15) who underwent home monitoring for six months and were chronically infected with *Pseudomonas*, and from bacteaemia infections (n = 365) as part of the UK BSAC bacteraemia resistance programme (62) and from patients attending hospitals in Cambridgeshire, UK were included. DNA was extracted using QIAxtractor (QIAgen), and samples were sequenced on the Illumina HiSeq 2000 and 2500 and X10 platforms.

Variants were called by mapping reads against the *P. aeruginosa* PAO1 reference genome (accession number AE004091.2) using the multiple_mappings_to_bam 1.6 pipeline with default parameters (https://github.com/sanger-pathogens/bact-gen-scripts) employing BWA (63) for mapping followed by stringent QC filtering and removing samples with an excess number of minority variants. Ariba 2.14.6 (64) was used for multi-locus sequencing typing (28). FastTree (2.1.10) was used to infer a global phylogenetic tree (65).

Clone were assigned by first grouping samples based on pairwise SNP distances using the ultra-metric pairwise group method with arithmetic means (UPGMA) and then applying a cut-off of 7000 SNPs. SNP-sites was used to infer a clone-specific alignment of variable sites (66). Gubbins version 2.4.1 (67) was used to remove recombination for individual clones with at least four available genomes.

### Dating and phylogeography

Molecular dating was performed for all 21 epidemic clone separately. Potential hypermutators (distorting the temporal signal) were removed by identifying samples with an unusual ratio of transition and transversion mutations. The temporal signal was assessed with TempEst (68) by comparing collection dates with root-to-tip distances using non-dated phylogenetic tress inferred with RAxML 8.2.12 (69). The significance of the signal was assessed using a permutation test using a custom script (https://github.com/chrisruis/tree_scripts/blob/main/bootstrap_TempEst_rttd_date.R). Clones with a significant temporal signal in this test (P < 0.05) were taken forward for molecular dating with BEAST 2.6.6 (29). We modelled the population history using the coalescent Bayesian skyline population prior. Convergence was assessed with Tracer 1.7.1 (70) with 10% burn-in. For clones that didn’t pass the bootstrap randomisation test (N = 9), a uniform prior for the substitution rate was set informed by the above clones.

For clones that passed the initial test, we ran a more thorough date randomisation test as described previously (71). The estimated median substitution rates and most recent common ancestor dates for randomised BEAST runs (n=10) did not overlap with those of the runs using real collection dates, indicating a significant temporal signal. To test whether each epidemic clone has undergone a historical population expansion, we analysed Bayesian skyline plot estimates of relative genetic diversity across the posterior distribution.

The association index was computed to find evidence of geographic clustering within clone phylogenies (72). We identified clones for further spatiotemporal analysis where less than 5% of randomisations had a higher association index than the non-permuted dataset. Asymmetric phylogeographic discrete trait reconstructions of the isolate continents were then performed using the BEAST classic 1.9.0 package of BEAST 2.6.6 (29). Subsampling to account for overrepresentation of certain continents was repeated five times and results compared between subsamples. Spread 0.9.7.1 (73) was used to identify candidate migration routes between continents (Bayes factor >= 3).

### Pan-genome analysis

Genomes were assembled from short-read data and Panaroo 1.2.8 (39) was used to cluster the gene sequences from all samples into gene families and to infer a graphical pan-genome, which was reduced, ordered against the *P. aeruginosa* PAO1 genome, pruned of long-range connections, and then visualised (see Supplementary Methods for details). Parsimony ancestral character state reconstruction was then used to infer gene gains and losses on the branches of the rooted tree leading to the ancestral epidemic and sporadic clones. Gene functions were then annotated using EggNog-mapper 2.1.6 (74), with the number of genes gained and annotated within a specific COG functional category compared using a Fisher exact test (adjusted p-value < 0.05).

### Macrophage infection experiments

Isogenic F508del homozygous THP1 cells were created from wild type THP1 cells (obtained from ATCC) using CRISPR-Cas9 editing and confirmed by Sanger sequencing (see Supplementary Methods). Wild type (WT) and F508del THP-1 monocytes were cultured, seeded at 200,000 cells/mL, and differentiated into macrophages (as previously described (75)) before being exposed to pooled clinical isolates of *P. aeruginosa* at a multiplicity of infection (MOI) of 1:1 and then incubated at 37 °C for 1 hour before the supernatant was removed and cells were lysed at 1h time point or incubated in fresh media for further time points (2h or 4h) before supernatant removal, cell lysis, and DNA extraction and sequencing. Strain abundance was quantified using the mSWEEP 1.4.0 sequence-based deconvolution method (76). Strains with less than 1% abundance at the 1h time point were excluded from the analysis.

### Transcriptomic analysis

Gene expression data for clinical *P. aeruginosa* strains (and the UCBPP- PA14 wildtype control strain) was obtained (25), and pseudoaligned to strain-specific gene indices to produce abundance estimates using Kallisto (77). Length-scaled abundance estimates were size-factor normalised by the median ratio method and modelled as a response to CF proportion per genomic cluster (as defined by the number of CF vs non-CF patients and environmental samples) using a negative binomial generalised linear model (GLM) with DESeq2 (78). The coefficients for gene models were assessed using the Wald test (adjusted p-value < 0.05). To assess the distribution and clustering of transcriptional diversity of strains with respect to CF proportion, we used k-means clustering (k=20) on the principal components (PCs) of the gene expression data, and then then calculated the mean standard deviation (*σ*) of the CF proportion by cluster (mean *σ* = 0.135). A permutation test was used to assess significance (see Supplementary Methods for details)

### Zebrafish infection models

The following zebrafish lines were used (see Supplementary Methods for details): wild type AB line; the knockout cftr sh540 mutant (46); Tg(mpeg1:mcherry-F)ump2 line (47). The morpholino for cftr knockdown (5’-GACACATTTTGGACACTCACACCAA-3’) were prepared and injected into one-cell-stage as previously described (79). Systemic infections were achieved by microinjection of GFP-expressing *P. aeruginosa* strains into the caudal vein of 30 hours post-fertilization (hpf) zebrafish embryos as previously described (80), with survival post infection assessed daily and viable *in vivo P. aeruginosa* quantified by colony forming units (CFU) at 1 day post infection (dpi). Macrophage responses were examined by intramuscular injection of anesthetized Tg(mpeg1:mcherry-F)ump2 larvae at 3 days post fertilisation (dpf) with GFP-expressing fluorescent *P. aeruginosa* as previously described (79, 81). Macrophage chemotaxis, phagocytosis, and intracellular *P. aeruginosa* burden were quantified by confocal microscopy (see Supplementary Methods for details).

### Mutational burden analysis

Treetime 0.8.1 (82) was used to reconstruct ancestral character states of every nucleotide position in every clone. We then implemented a pipeline (83) to identify single nucleotide changes and annotate variant effect in their phylogenetic context using the gene annotation from Pseudomonas.com (PAO1 107) and the ancestral character state reconstructions (55). Parsimony ancestral character state reconstruction was used to infer ancestral insertions and deletions, using SNPeff (48) for variant effect annotation.

We assessed the mutational burden of every gene based on the number of non-synonymous variants across all clones (using a Poisson test, adjusted p-value < 0.05). The 224 genes passing the adjusted p-value threshold were used to query the STRING 11.5 database (56) of protein-protein interaction. Pathoadaptive genes were assigned to 17 functional categories based on the gene products description on Pseudomonas.com (55) (Figure S1). A Fisher exact test was used to compare the number of assigned with the number of unassigned genes among pathoadaptive genes and non-hits.

### Impact of amino acid changes on protein stability and structural analysis

All amino acid changes were analysed with SIFT 4G 6.2.1 (49) and FoldX 5 (50) (see Supplementary Methods for details). A two-tailed t-test was used to compare the averaged scores per gene/protein scores between mutational burden test hits and non-hits. Mutational frequencies were mapped on the structural models of the identified hotspot genes in *P. aeruginosa* using the Chimera molecular modelling package (84). Models were downloaded from the Protein Data Bank and UniProt (85).

### Phenotyping of pathoadaptive gene mutations

PAO1 mutants with transposon insertions in 154 pathoadaptive genes (selected from the Manoil library (86) were arrayed in 96 well plates and imaged using the Phenobooth Imager (Singer Instruments) to quantify the following phenotypic traits: swimming motility, twitching motility, siderophore production, caseinase activity, gelatinase activity, and rhamnolipid production (see Supplementary Methods for details).

To assess the association between genetic variants and the expression of transcription factor (TF) regulons, gene expression data from (25) were pseudoaligned to strain-specific gene sets and the normalised expression levels of TF regulons were compared between strains with and without genetic variants using Welch’s two-sample *t*-tests (adjusted p-value < 0.05) (see Supplementary Methods for details).

### Transmission and host selectivity of pathoadaptive mutations

To assess the transmissibility of pathoadaptive changes, the number of mutations that had been observed in at least two isolates (from different patients) was compared with hitherto untransmitted mutations using a Fisher exact test (adjusted p-value < 0.1). TopGO 2.4.6 was used for functional enrichment analysis of the host-specific Gene Ontology biological pathway annotation compared to background (57), using annotations from *Pseudomonas.com* (55) (p-value < 0.05).

Mutations in pathoadaptative genes were stratified by the (ancestral) infection type (CF or non-CF) of every branch based on outgroup-rooted rooted clone trees. To assess host-specific pathoadapdation, the number of CF vs non-CF mutations were compared using a Fisher exact test (adjusted p-value < 0.1). Mutations on branches with non-concordant ancestral infection types were discarded.

Trajectories were inferred as the sequence of mutations in pathoadaptive genes since the emergence of the clone ancestor as implied by the PAO1-rooted tree stratified by cystic fibrosis (CF) and non-CF infection types. Mutation frequencies were position normalised and the frequency plots of the 40 genes with the lowest p-value from the mutational-burden test were manually assigned into five groups of genes with similar frequency curve shapes. Trendlines were generated by locally-weighted smoothing.

We established a relatedness cut-off to define potential transmission links using pairwise SNP differences between pairs of isolate genomes from the same patient (n = 81 patients). We then identified potential transmission events as isolates from the same clone sampled from different patients that differed by 26 SNPs or fewer, visualised using Cytoscape.

## Supplementary Material

Fig.1

Fig.2

## Data Availability

Short-read DNA and RNA sequence data for the clinical isolates was downloaded from the European Nucleotide Archive (ENA). THP1 pooled infection assay DNA sequencing data was uploaded to the ENA under PRJEB20836 (Supplementary Table S4). Short-read DNA sequencing data for newly sequenced isolates genomes (or where only assemblies were previously available) was uploaded to the ENA (TeleCF: ERP022089, IPC: PRJNA325248, AZ: PRJEB66158, LES: PRJEB69223, Bronchiectasis UK PRJEB69219). ENA run accessions can be found in Supplementary Table S1. The analysis codes and the PhyloEffects software were made available on GitHub and snapshots provided on Zenodo (83, 87). Intermediate and additional data were made available in a Zenodo repository (88).

## References

[1] Whitby JL, Rampling A (1972). Pseudomonas Æruginosa contamination in domestic and hospital environments. Lancet.

[2] Green SK, Schroth MN, Cho JJ, Kominos SK, Vitanza-jack VB (1974). Agricultural plants and soil as a reservoir for Pseudomonas aeruginosa. Appl Microbiol.

[3] Pirnay J-P, Matthijs S, Colak H, Chablain P, Bilocq F, Van Eldere J, De Vos D, Zizi M, Triest L, Cornelis P (2005). Global Pseudomonas aeruginosa biodiversity as reflected in a Belgian river. Environ Microbiol.

[4] Ringen LM, Drake CH (1952). A study of the incidence of Pseudomonas aeruginosa from various natural sources. J Bacteriol.

[5] Crone S, Vives-Flórez M, Kvich L, Saunders AM, Malone M, Nicolaisen MH, Martínez-García E, Rojas-Acosta C, Catalina Gomez-Puerto M, Calum H, Whiteley M (2020). The environmental occurrence of Pseudomonas aeruginosa. APMIS.

[6] ECDC (2019). Healthcare-associated infections acquired in intensive care units Annual epidemiological report for 2017.

[7] Murphy TF, Brauer AL, Eschberger K, Lobbins P, Grove L, Cai X, Sethi S (2008). Pseudomonas aeruginosa in chronic obstructive pulmonary disease. Am J Respir Crit Care Med.

[8] Rajan S, Saiman L (2002). Pulmonary infections in patients with cystic fibrosis. Semin Respir Infect.

[9] Finch S, McDonnell MJ, Abo-Leyah H, Aliberti S, Chalmers JD (2015). A Comprehensive Analysis of the Impact of Pseudomonas aeruginosa Colonization on Prognosis in Adult Bronchiectasis. Ann Am Thorac Soc.

[10] Kosorok MR, Zeng L, West SE, Rock MJ, Splaingard ML, Laxova A, Green CG, Collins J, Farrell PM (2001). Acceleration of lung disease in children with cystic fibrosis after Pseudomonas aeruginosa acquisition. Pediatr Pulmonol.

[11] Tacconelli E, Carrara E, Savoldi A, Harbarth S, Mendelson M, Monnet DL, Pulcini C, Kahlmeter G, Kluytmans J, Carmeli Y, Ouellette M, WHO Pathogens Priority List Working Group (2018). Discovery, research, and development of new antibiotics: the WHO priority list of antibiotic-resistant bacteria and tuberculosis. Lancet Infect Dis.

[12] Ikuta KS (2022). Global mortality associated with 33 bacterial pathogens in 2019: a systematic analysis for the Global Burden of Disease Study 2019. Lancet.

[13] Mitchelmore PJ, Randall J, Bull MJ, Moore KA, O’Neill PA, Paszkiewicz K, Mahenthiralingam E, Scotton CJ, Sheldon CD, Withers NJ, Brown AR (2017). Molecular epidemiology of Pseudomonas aeruginosa in an unsegregated bronchiectasis cohort sharing hospital facilities with a cystic fibrosis cohort. Thorax.

[14] Stapleton PJ, Izydorcyzk C, Clark S, Blanchard A, Wang PW, Yau Y, Waters V, Guttman DS (2020). Pseudomonas aeruginosa strain sharing in early infection among children with cystic fibrosis. Clin Infect Dis.

[15] Tramper-Stranders GA, van der Ent CK, Wolfs TFW, Kimpen JLL, Fleer A, Johansen U, Johansen HK, Høiby N (2008). Pseudomonas aeruginosa diversity in distinct paediatric patient groups. Clin Microbiol Infect.

[16] Woo TE, Lim R, Surette MG, Waddell B, Bowron JC, Somayaji R, Duong J, Mody CH, Rabin HR, Storey DG, Parkins MD (2018). Epidemiology and natural history of Pseudomonas aeruginosa airway infections in non-cystic fibrosis bronchiectasis. ERJ Open Res.

[17] AbdulWahab A, Taj-Aldeen SJ, Ibrahim E, Abdulla SH, Muhammed R, Ahmed I, Abdeen Y, Sadek O, Abu-Madi M (2014). Genetic relatedness and host specificity of Pseudomonas aeruginosa isolates from cystic fibrosis and non-cystic fibrosis patients. Infect Drug Resist.

[18] Kos VN, Déraspe M, McLaughlin RE, Whiteaker JD, Roy PH, Alm RA, Corbeil J, Gardner H (2015). The resistome of Pseudomonas aeruginosa in relationship to phenotypic susceptibility. Antimicrob Agents Chemother.

[19] Moore MP, Lamont IL, Williams D, Paterson S, Kukavica-Ibrulj I, Tucker NP, Kenna DTD, Turton JF, Jeukens J, Freschi L, Wee BA (2021). Transmission, adaptation and geographical spread of the Pseudomonas aeruginosa Liverpool epidemic strain. Microb Genom.

[20] López-Causapé C, Sommer LM, Cabot G, Rubio R, Ocampo-Sosa AA, Johansen HK, Figuerola J, Cantón R, Kidd TJ, Molin S, Oliver A (2017). Evolution of the Pseudomonas aeruginosa mutational resistome in an international Cystic Fibrosis clone. Sci Rep.

[21] Freschi L, Vincent AT, Jeukens J, Emond-Rheault J-G, Kukavica-Ibrulj I, Dupont M-J, Charette SJ, Boyle B, Levesque RC (2018). The Pseudomonas aeruginosa pan-genome provides new insights on its population structure, horizontal gene transfer and pathogenicity. Genome Biol Evol.

[22] Hilliam Y, Moore MP, Lamont IL, Bilton D, Haworth CS, Foweraker J, Walshaw MJ, Williams D, Fothergill JL, De Soyza A, Winstanley C (2017). Pseudomonas aeruginosa adaptation and diversification in the non-cystic fibrosis bronchiectasis lung. Eur Respir J.

[23] Chilam J, Argimón S, Limas MT, Masim ML, Gayeta JM, Lagrada ML, Olorosa AM, Cohen V, Hernandez LT, Jeffrey B, Abudahab K (2021). Philippines Antimicrobial Resistance Surveillance Program, Genomic surveillance of Pseudomonas aeruginosa in the Philippines, 2013-2014. Western Pac Surveill Response J.

[24] Del Barrio-Tofiño E, López-Causapé C, Cabot G, Rivera A, Benito N, Segura C, Montero MM, Sorlí L, Tubau F, Gómez-Zorrilla S, Tormo N (2017). Genomics and Susceptibility Profiles of Extensively Drug-Resistant Pseudomonas aeruginosa Isolates from Spain. Antimicrob Agents Chemother.

[25] Khaledi A, Weimann A, Schniederjans M, Asgari E, Kuo T-H, Oliver A, Cabot G, Kola A, Gastmeier P, Hogardt M, Jonas D (2020). Predicting antimicrobial resistance in Pseudomonas aeruginosa with machine learning-enabled molecular diagnostics. EMBO Mol Med.

[26] Marvig RL, Sommer LM, Molin S, Johansen HK (2015). Convergent evolution and adaptation of Pseudomonas aeruginosa within patients with cystic fibrosis. Nat Genet.

[27] Turton JF, Wright L, Underwood A, Witney AA, Chan Y-T, Al-Shahib A, Arnold C, Doumith M, Patel B, Planche TD, Green J (2015). High-Resolution Analysis by Whole-Genome Sequencing of an International Lineage (Sequence Type 111) of Pseudomonas aeruginosa Associated with Metallo-Carbapenemases in the United Kingdom. J Clin Microbiol.

[28] Curran B, Jonas D, Grundmann H, Pitt T, Dowson CG (2004). Development of a multilocus sequence typing scheme for the opportunistic pathogen Pseudomonas aeruginosa. J Clin Microbiol.

[29] Bouckaert R, Heled J, Kühnert D, Vaughan T, Wu C-H, Xie D, Suchard MA, Rambaut A, Drummond AJ (2014). BEAST 2: a software platform for Bayesian evolutionary analysis. PLoS Comput Biol.

[30] Drummond AJ, Rambaut A, Shapiro B, Pybus OG (2005). Bayesian coalescent inference of past population dynamics from molecular sequences. Mol Biol Evol.

[31] McNeill WH (1984). Human Migration in Historical Perspective. Popul Dev Rev.

[32] Dobson AP, Carper ER (1996). Infectious Diseases and Human Population History. Bioscience.

[33] Kelly FJ, Fussell JC (2011). Air pollution and airway disease. Clin Exp Allergy.

[34] Chauhan AJ, Johnston SL (2003). Air pollution and infection in respiratory illness. Br Med Bull.

[35] Kampa M, Castanas E (2008). Human health effects of air pollution. Environ Pollut.

[36] Lemey P, Rambaut A, Drummond AJ, Suchard MA (2009). Bayesian phylogeography finds its roots. PLoS Comput Biol.

[37] Katsnelson MI, Wolf YI, Koonin EV (2019). On the feasibility of saltational evolution. Proc Natl Acad Sci USA.

[38] Bryant JM, Brown KP, Burbaud S, Everall I, Belardinelli JM, Rodriguez-Rincon D, Grogono DM, Peterson CM, Verma D, Evans IE, Ruis C (2021). Stepwise pathogenic evolution of *Mycobacterium abscessus*. Science.

[39] Tonkin-Hill G, MacAlasdair N, Ruis C, Weimann A, Horesh G, Lees JA, Gladstone RA, Lo S, Beaudoin C, Floto RA, Frost SDW (2020). Producing polished prokaryotic pangenomes with the Panaroo pipeline. Genome Biol.

[40] Martin K, Baddal B, Mustafa N, Perry C, Underwood A, Constantidou C, Loman N, Kenna DT, Turton JF (2013). Clusters of genetically similar isolates of Pseudomonas aeruginosa from multiple hospitals in the UK. J Med Microbiol.

[41] Day J, Friedman A, Schlesinger LS (2009). Modeling the immune rheostat of macrophages in the lung in response to infection. Proc Natl Acad Sci USA.

[42] Craig A, Mai J, Cai S, Jeyaseelan S (2009). Neutrophil recruitment to the lungs during bacterial pneumonia. Infect Immun.

[43] Fortuna A, Collalto D, Schiaffi V, Pastore V, Visca P, Ascenzioni F, Rampioni G, Leoni L (2022). The Pseudomonas aeruginosa DksA1 protein is involved in H2O2 tolerance and within-macrophages survival and can be replaced by DksA2. Sci Rep.

[44] Fortuna A, Bähre H, Visca P, Rampioni G, Leoni L (2021). The two Pseudomonas aeruginosa DksA stringent response proteins are largely interchangeable at the whole transcriptome level and in the control of virulence-related traits. Environ Microbiol.

[45] Turton KB, Ingram RJ, Valvano MA (2021). Macrophage dysfunction in cystic fibrosis: Nature or nurture?. J Leukoc Biol.

[46] Bernut A, Loynes CA, Floto RA, Renshaw SA (2020). Deletion of cftr Leads to an Excessive Neutrophilic Response and Defective Tissue Repair in a Zebrafish Model of Sterile Inflammation. Front Immunol.

[47] Bernut A, Herrmann J-L, Kissa K, Dubremetz J-F, Gaillard J-L, Lutfalla G, Kremer L (2014). Mycobacterium abscessus cording prevents phagocytosis and promotes abscess formation. Proc Natl Acad Sci USA.

[48] Cingolani P, Platts A, Wang LL, Coon M, Nguyen T, Wang L, Land SJ, Lu X, Ruden DM (2012). A program for annotating and predicting the effects of single nucleotide polymorphisms, SnpEff: SNPs in the genome of Drosophila melanogaster strain w1118; iso-2; iso-3. Fly.

[49] Vaser R, Adusumalli S, Leng SN, Sikic M, Ng PC (2016). SIFT missense predictions for genomes. Nat Protoc.

[50] Delgado J, Radusky LG, Cianferoni D, Serrano L (2019). FoldX 5.0: working with RNA, small molecules and a new graphical interface. Bioinformatics.

[51] Westbrock-Wadman S, Sherman DR, Hickey MJ, Coulter SN, Zhu YQ, Warrener P, Nguyen LY, Shawar RM, Folger KR, Stover CK (1999). Characterization of a Pseudomonas aeruginosa efflux pump contributing to aminoglycoside impermeability. Antimicrob Agents Chemother.

[52] Poole K, Tetro K, Zhao Q, Neshat S, Heinrichs DE, Bianco N (1996). Expression of the multidrug resistance operon mexA-mexB-oprM in Pseudomonas aeruginosa: mexR encodes a regulator of operon expression. Antimicrob Agents Chemother.

[53] Rust L, Pesci EC, Iglewski BH (1996). Analysis of the Pseudomonas aeruginosa elastase (lasB) regulatory region. J Bacteriol.

[54] Yahr TL, Frank DW (1994). Transcriptional organization of the trans-regulatory locus which controls exoenzyme S synthesis in Pseudomonas aeruginosa. J Bacteriol.

[55] Winsor GL, Griffiths EJ, Lo R, Dhillon BK, Shay JA, Brinkman FSL (2016). Enhanced annotations and features for comparing thousands of Pseudomonas genomes in the Pseudomonas genome database. Nucleic Acids Res.

[56] Szklarczyk D, Gable AL, Lyon D, Junge A, Wyder S, Huerta-Cepas J, Simonovic M, Doncheva NT, Morris JH, Bork P, Jensen LJ (2018). STRING v11: protein–protein association networks with increased coverage, supporting functional discovery in genome-wide experimental datasets. Nucleic Acids Res.

[57] Alexa A, Rahnenführer J, Lengauer T (2006). Improved scoring of functional groups from gene expression data by decorrelating GO graph structure. Bioinformatics.

[58] Martin DW, Schurr MJ, Yu H, Deretic V (1994). Analysis of promoters controlled by the putative sigma factor AlgU regulating conversion to mucoidy in Pseudomonas aeruginosa: relationship to sigma E and stress response. J Bacteriol.

[59] Broder UN, Jaeger T, Jenal U (2016). LadS is a calcium-responsive kinase that induces acute-to-chronic virulence switch in Pseudomonas aeruginosa. Nat Microbiol.

[60] Chen C, Beattie GA (2008). Pseudomonas syringae BetT is a low-affinity choline transporter that is responsible for superior osmoprotection by choline over glycine betaine. J Bacteriol.

[61] Santi I, Manfredi P, Maffei E, Egli A, Jenal U (2021). Evolution of Antibiotic Tolerance Shapes Resistance Development in Chronic Pseudomonas aeruginosa Infections. MBio.

[62] Reynolds R, Hope R, Williams L (2008). BSAC Working Parties on Resistance Surveillance, Survey, laboratory and statistical methods for the BSAC Resistance Surveillance Programmes. J Antimicrob Chemother.

[63] Li H (2013). Aligning sequence reads, clone sequences and assembly contigs with BWA-MEM. arXiv [q-bioGN].

[64] Hunt M, Mather AE, Sánchez-Busó L, Page AJ, Parkhill J, Keane JA, Harris SR (2017). ARIBA: rapid antimicrobial resistance genotyping directly from sequencing reads. Microb Genom.

[65] Price MN, Dehal PS, Arkin AP (2010). FastTree 2--approximately maximum-likelihood trees for large alignments. PLoS One.

[66] Page AJ, Taylor B, Delaney AJ, Soares J, Seemann T, Keane JA, Harris SR (2016). SNP-sites: rapid efficient extraction of SNPs from multi-FASTA alignments. Microb Genom.

[67] Croucher NJ, Page AJ, Connor TR, Delaney AJ, Keane JA, Bentley SD, Parkhill J, Harris SR (2015). Rapid phylogenetic analysis of large samples of recombinant bacterial whole genome sequences using Gubbins. Nucleic Acids Res.

[68] Rambaut A, Lam TT, Max Carvalho L, Pybus OG (2016). Exploring the temporal structure of heterochronous sequences using TempEst (formerly Path-O-Gen). Virus Evol.

[69] Stamatakis A (2014). RAxML version 8: a tool for phylogenetic analysis and post-analysis of large phylogenies. Bioinformatics.

[70] Rambaut A, Drummond AJ, Xie D, Baele G, Suchard MA (2018). Posterior Summarization in Bayesian Phylogenetics Using Tracer 1.7. Syst Biol.

[71] Menardo F, Duchêne S, Brites D, Gagneux S (2019). The molecular clock of Mycobacterium tuberculosis. PLoS Pathog.

[72] Parker J, Rambaut A, Pybus OG (2008). Correlating viral phenotypes with phylogeny: accounting for phylogenetic uncertainty. Infect Genet Evol.

[73] Bielejec F, Rambaut A, Suchard MA, Lemey P (2011). SPREAD: spatial phylogenetic reconstruction of evolutionary dynamics. Bioinformatics.

[74] Huerta-Cepas J, Forslund K, Coelho LP, Szklarczyk D, Jensen LJ, von Mering C, Bork P (2017). Fast Genome-Wide Functional Annotation through Orthology Assignment by eggNOG-Mapper. Mol Biol Evol.

[75] Bryant JM, Grogono DM, Rodriguez-Rincon D, Everall I, Brown KP, Moreno P, Verma D, Hill E, Drijkoningen J, Gilligan P, Esther CR (2016). Emergence and spread of a human-transmissible multidrug-resistant nontuberculous mycobacterium. Science.

[76] Mäklin T, Kallonen T, David S, Boinett CJ, Pascoe B, Méric G, Aanensen DM, Feil EJ, Baker S, Parkhill J, Sheppard SK (2020). High-resolution sweep metagenomics using fast probabilistic inference. Wellcome Open Res.

[77] Bray NL, Pimentel H, Melsted P, Pachter L (2016). Near-optimal probabilistic RNA-seq quantification. Nat Biotechnol.

[78] Love M, Anders S, Huber W (2014). Differential analysis of count data--the DESeq2 package. Genome Biol.

[79] Bernut A, Dupont C, Ogryzko NV, Neyret A, Herrmann J-L, Floto RA, Renshaw SA, Kremer L (2019). CFTR Protects against Mycobacterium abscessus Infection by Fine-Tuning Host Oxidative Defenses. Cell Rep.

[80] Belon C, Soscia C, Bernut A, Laubier A, Bleves S, Blanc-Potard A-B (2015). A Macrophage Subversion Factor Is Shared by Intracellular and Extracellular Pathogens. PLoS Pathog.

[81] Le Moigne V, Roux A-L, Mahoudo H, Christien G, Ferroni A, Dumitrescu O, Lina G, Bouchara J-P, Plésiat P, Gaillard J-L, Canaan S (2022). Serological biomarkers for the diagnosis of Mycobacterium abscessus infections in cystic fibrosis patients. J Cyst Fibros.

[82] Sagulenko P, Puller V, Neher RA (2018). TreeTime: Maximum-likelihood phylodynamic analysis. Virus Evol.

[83] Weimann A, Ruis C (2024). PhyloEffects. Zenodo.

[84] Pettersen EF, Goddard TD, Huang CC (2004). UCSF Chimera—a visualization system for exploratory research and analysis. Journal of.

[85] Suzek BE, Huang H, McGarvey P, Mazumder R, Wu CH (2007). UniRef: comprehensive and non-redundant UniProt reference clusters. Bioinformatics.

[86] Held K, Ramage E, Jacobs M, Gallagher L, Manoil C (2012). Sequence-verified two-allele transposon mutant library for Pseudomonas aeruginosa PAO1. J Bacteriol.

[87] Weimann A, Dinan A (2024). Computer codes for Weimann et al - Evolution and host-specific pathoadaptation of Pseudomonas aeruginosa. Zenodo.

[88] Weimann A (2024). Data for Weimann et al - Evolution and host-specific pathoadaptation of Pseudomonas aeruginosa. Zenodo.

[89] Ashburner M, Ball CA, Blake JA, Botstein D, Butler H, Cherry JM, Davis AP, Dolinski K, Dwight SS, Eppig JT, Harris MA (2000). Gene Ontology: tool for the unification of biology. Nat Genet.

[90] McKenna A, Hanna M, Banks E, Sivachenko A, Cibulskis K, Kernytsky A, Garimella K, Altshuler D, Gabriel S, Daly M, DePristo MA (2010). The Genome Analysis Toolkit: a MapReduce framework for analyzing next-generation DNA sequencing data. Genome Res.

[91] Li H (2011). A statistical framework for SNP calling, mutation discovery, association mapping and population genetical parameter estimation from sequencing data. Bioinformatics.

[92] Danecek P, Bonfield JK, Liddle J, Marshall J, Ohan V, Pollard MO, Whitwham A, Keane T, McCarthy SA, Davies RM, Li H (2021). Twelve years of SAMtools and BCFtools. Gigascience.

[93] Jolley KA, Bray JE, Maiden MCJ (2018). Open-access bacterial population genomics: BIGSdb software, the Pubmlst.org website and their applications. Wellcome Open Res.

[94] Yu G, Smith DK, Zhu H, Guan Y, Lam TT-Y (2017). Ggtree : An r package for visualization and annotation of phylogenetic trees with their covariates and other associated data. Methods Ecol Evol.

[95] Page AJ, De Silva N, Hunt M, Quail MA, Parkhill J, Harris SR, Otto TD, Keane JA (2016). Robust high-throughput prokaryote de novo assembly and improvement pipeline for Illumina data. Microb Genom.

[96] Zerbino DR, Birney E (2008). Velvet: algorithms for de novo short read assembly using de Bruijn graphs. Genome Res.

[97] Bankevich A, Nurk S, Antipov D, Gurevich AA, Dvorkin M, Kulikov AS, Lesin VM, Nikolenko SI, Pham S, Prjibelski AD, Pyshkin AV (2012). SPAdes: a new genome assembly algorithm and its applications to single-cell sequencing. J Comput Biol.

[98] Shannon P, Markiel A, Ozier O, Baliga NS, Wang JT, Ramage D, Amin N, Schwikowski B, Ideker T (2003). Cytoscape: a software environment for integrated models of biomolecular interaction networks. Genome Res.

[99] Cantalapiedra CP, Hernández-Plaza A, Letunic I, Bork P, Huerta-Cepas J (2021). eggNOG-mapper v2: Functional Annotation, Orthology Assignments, and Domain Prediction at the Metagenomic Scale. Mol Biol Evol.

[100] Valley HC, Bukis KM, Bell A, Cheng Y, Wong E, Jordan NJ, Allaire NE, Sivachenko A, Liang F, Bihler H, Thomas PJ (2019). Isogenic cell models of cystic fibrosis-causing variants in natively expressing pulmonary epithelial cells. J Cyst Fibros.

[101] Figurski DH, Helinski DR (1979). Replication of an origin-containing derivative of plasmid RK2 dependent on a plasmid function provided in trans. Proc Natl Acad Sci USA.

[102] Nivens DE, Ohman DE, Williams J, Franklin MJ (2001). Role of alginate and its O acetylation in formation of Pseudomonas aeruginosa microcolonies and biofilms. J Bacteriol.

[103] Benjamini Y, Hochberg Y (1995). Controlling the False Discovery Rate: A Practical and Powerful Approach to Multiple Testing. J R Stat Soc Series B Stat Methodol.

[104] Louden BC, Haarmann D, Lynne AM (2011). Use of Blue Agar CAS Assay for Siderophore Detection. J Microbiol Biol Educ.

[105] Varadi M, Anyango S, Deshpande M, Nair S, Natassia C, Yordanova G, Yuan D, Stroe O, Wood G, Laydon A, Žídek A (2022). AlphaFold Protein Structure Database: massively expanding the structural coverage of protein-sequence space with high-accuracy models. Nucleic Acids Res.

