# Supplementary material for: Evolution and host-specific adaptation of Pseudomonas aeruginosa
Source: Science. Author manuscript; Available in PMC 2025 Nov 18. (PMC7618370; doi:10.1126/science.adi0908)
Supplement: Fig.1 [file EMS209707-supplement-Fig_1.pdf]

Supplementary Materials for

Evolution and host-specific adaptation of *Pseudomonas aeruginosa*

Aaron Weimann[1,2,3,4], Adam M Dinan[1,2,3], Christopher Ruis[1,2,3,4], Audrey Bernut[5], Stéphane Pont[5], Karen Brown[1,2,6], Judy Ryan[1,2], Lúcia Santos[7], Louise Ellison[2], Emem Ukor[2,6], Arun P. Pandurangan[1,8,9], Sina Krokowski[1,2], Tom L. Blundell[1,8,9], Martin Welch[8], Beth Blane[9], Kim Judge[10], Rachel Bousfield[9,11], Nicholas Brown[11], Josephine M. Bryant[10], Irena Kukavica-Ibrulj[12], Giordano Rampioni[13,14], Livia Leoni[13], Patrick T. Harrison[7], Sharon J Peacock[9,11], Nicholas R. Thomson[10], Jeff Gauthier[12], Jo L Fothergill[15], Roger C Levesque[12], Julian Parkhill[4], R. Andres Floto[1,2,3,6,9,11]

Corresponding author:
Andres Floto (arf27@cam.ac.uk),
Julian Parkhill (jp369@cam.ac.uk), or
Roger Levesque (rclevesq@ibis.ulaval.ca)

The PDF file includes:

Materials and Methods
Figs. S1 to S16
Captions for Tables S1 to S7
References (90-105)

Other Supplementary Materials for this manuscript include the following:

Supplementary Tables 1-7 (tsv)

MATERIAL AND METHODS

Genomic datasets

Published datasets: We utilized the following *Pseudomonas aeruginosa* whole genome sequencing datasets: from studies of antibiotic resistance in healthcare setting in Germany (*25*), Spain (*24*), the Philippines (*23*), and world-wide (*18*); from studies of infection in individuals with cystic fibrosis (CF) from Denmark (*26*) and Canada (*14*) and with non-CF bronchiectasis from the UK(*22*); from the International *Pseudomonas* Consortium (*21*); and from studies targeting high-risk clones ST274 (*20*), ST111 (*27*), and the Liverpool Epidemic Strain (LES) (*19*).

Unpublished datasets: P. aeruginosa isolates from the TeleCF study: TeleCF (NCT01877707) was a single-centre, observational pilot study of home monitoring in adults with CF (*n* = 9) who were chronically infected with *P. aeruginosa* and had experienced at least two acute pulmonary exacerbations in the prior 12 months. Participants collected spontaneously expectorated sputum daily for 6 months in dedicated study freezers provided in their homes. Samples were collected and then thawed to room temperature, treated with an equal volume of 0.1% dithiothreitol, vortexed, incubated at room temperature for 15 minutes, and then vortexed again to ensure homogeneity before being streaked to purity and grown on *Pseudomonas* selective agar base prepared plates with cetrimide and sodium nalidixate supplement (PCN agar; Oxoid). Plates were incubated at 37°C for 48-72 h, to allow for the growth of slow-replicating and small colony variants. Representative colonies were placed into a 96-deep well microplate containing 1 mL of cetrimide broth, and grown for 6 hours at 37°C. These strains were then re-streaked for purity and grown on PCN plates at 37°C for 48-72 hours. Single colonies were chosen and re-arrayed into individual 96-deep well microplates, one microplate per initial sputum sample, and left to undergo static incubation at 37°C for a further 6 hours. To ensure minimal adaptation to the laboratory environment, the strains were stored at -80°C in 25% (v/v) glycerol solution for long-term preservation. DNA was extracted from the strains using a QIAxtractor (QIAgen) instrument, following the manufacturer's instructions. The Illumina protocol was used for library preparation, and sequencing was conducted on both the Illumina HiSeq 2500 and X10 platforms.

P. aeruginosa isolates from bacteraemia infections (n = 365): 224 isolates were cultured from bloodstream infections from patients attending Addenbrookes, Hinchingbrooke, and Papworth Hospitals (UK) between 2006 and 2013 with a further 60 isolates collected between 2017 and 2018. 81 isolates were collected as part of the BSAC bacteraemia resistance programme (*62*) from 25 contributing laboratories distributed across the UK and Ireland between 2001 and 2011 focusing on multi-drug resistant strains. DNA was extracted using QIAxtractor (QIAgen), according to the manufacturer's instructions. Library preparation was done according to the Illumina protocol, and samples were sequenced on the Illumina HiSeq 2000 and 2500 platforms.

Variant calling

Sequencing reads from all samples analysed were mapped against the *P. aeruginosa* PAO1 reference genome (accession number AE004091.2) using the multiple_mappings_to_bam 1.6 pipeline with default parameters (https://github.com/sanger-pathogens/bact-gen-scripts) with BWA (*63*) as the short-read aligner including an indel realignment step using GATK (*90*). Samples where less than 70% of the reads mapped to the reference genome were removed.

SNPs and indels were identified from the aligned reads using the same pipeline employing samtools mpileup (*91*) for generating read pile-ups and bcftools call (*92*) for variant identification. Variants were filtered by base call quality (>=50), mapping quality (>=20) and number of supporting reads (>=8) on the reverse strand (>=3) and the forward strand (>=3). Indels across strains were aggregated into a catalogue and the base at the start of every indel in the catalogue was quality checked as above.

We removed samples with an excess number of minority variants as they likely indicate strain-level contamination. Variants were filtered to identify minority variants using a previously described approach (*38*). Subsequently every sample with more than 8 minority variants was removed. Ariba 2.14.6 (*64*) was used to query the genomes using the multi-locus sequencing type (ST) scheme for *P. aeruginosa* (*28*) for the allele-sequence type combinations as available on pubMLST (*93*) (downloaded on Nov 11 2020). Ariba also identifies new allelic combinations. Samples where the sequence type could not be determined with Ariba were removed (n = 7). The oldest sample was chosen to represent every patient sequence type combination to minimise bias from within-host evolution. SNP-sites (*66*) was used to infer an alignment of the variable sites. FastTree (2.1.10) was used to infer a global phylogenetic tree (*65*) representing all environmental and animal isolates together with the earliest isolate of every majority ST per patient. Ggtree was used to visualise the trees and produce figures (*94*).

Clone assignment

The sample with the earliest collection date in each sequence type was chosen to represent every patient-sequence type combination to minimise bias from within-host evolution. PairSNP (Version 0.2.0) (https://github.com/gtonkinhill/pairsnp) was used to infer all pairwise SNP distances between sample pairs. The ultra-metric pairwise group method with arithmetic means (UPGMA) was used to cluster samples based on pairwise SNP distances. UPGMA infers a sample dendrogram which is then separated into clusters by applying a discrete SNP distance threshold. A global threshold of 7000 SNPs was chosen to separate the dendrogram into genomic clusters (which we refer to as clones). This threshold was selected to assign all samples from any recently emerged clones into the same cluster while at the same time being liberal enough to allow recently emerged recombinants and hypermutators to be clustered within their ancestral clones (*Supplementary Figure 1*). Samples with the same patient-sequence type combinations that were not included in the clustering were then assigned to the matching clone type. SNP-sites was used to infer a clone-specific alignment of variable sites (*66*). Gubbins version 2.4.1 (*67*) was used to remove recombination for individual clones with at least four available genomes. Gubbins infers a phylogenetic tree with RaxML (*69*) based on the final alignment.

Dating and phylogeography

Molecular dating was performed for all 21 epidemic clones separately including genome sequences with known collection dates and including only the earliest sample from each patient. To identify potential hypermutator branches that would violate the molecular clock assumption, the ratio of transition and transversion mutations was compared using a Fisher exact test. P-values were corrected using the Benjamini-Hochberg procedure at an FDR threshold of 5%. We initially assessed the temporal signal in each epidemic clone. We reconstructed a non-dated phylogenetic tree using RAxML 8.2.12 (*69*) with the general time reversible (GTR) model of nucleotide substitution and gamma rate heterogeneity with four gamma classes and compared sequence collection dates with root-to-tip distances using TempEst (*68*). Sequences that had accumulated far more or fewer mutations than expected given their collection date were removed from further analyses and a new phylogenetic tree reconstructed as above. To assess the significance of the temporal signal in each epidemic clone, we compared the correlation coefficient between collection date and root-to-tip distance with the real sequence dates with the distribution of correlation coefficients from 1000 date randomisations using a custom script (https://github.com/chrisruis/tree_scripts/blob/_main/bootstrap_TempEst__rttd_date.R). Clones with a significant temporal signal in this test ($P < 0.05$) were taken forward for molecular dating with BEAST 2.6.6 (*29*). We employed the HKY model of nucleotide substitution. We used an uncorrelated relaxed lognormal clock model with a lognormal prior on the mean substitution rate with mean set to the slope of the root-to-tip correlation calculated above and standard deviation set to 0.5. The variation in substitution rate across branches was modelled using a gamma prior

with alpha set to 0.54 and beta set to 0.38. We modelled the population history using the coalescent Bayesian skyline population prior. Three independent runs were conducted for each dataset with 100M steps; convergence was assessed with Tracer 1.7.1 (*70*) with 10% burn-in. For clones that didn't pass the bootstrap randomisation test (N = 9), a uniform prior for the substitution rate was set encompassing values across the 95% HPD estimates for the substitution rates inferred for the above clones. All other priors and parameters were kept the same.

To further establish a temporal signal in epidemic clone that passed the root-to-tip randomization test above, we ran a more thorough date randomisation test (*71*). Here, BEAST was run using a uniform prior with upper and lower bounds set to encompass the full range of substitution rates inferred previously for the clones above and all other priors set as before. The estimates from those BEAST runs were highly similar to the estimates obtained with the informed substitution rate prior. We then performed ten randomization runs where collection dates were randomized across sequences and BEAST run using the same uniform prior on substitution rate. The estimated median substitution rates and most recent common ancestor dates for the randomization runs did not overlap with those of the runs using real collection dates, indicating a significant temporal signal.

To test whether each epidemic clone has undergone a historical population expansion, we analysed Bayesian skyline plot estimates of relative genetic diversity across the posterior distribution. We inferred whether each sampled step in the MCMC chain exhibits an increase in relative genetic diversity of at least twofold relative to the root of the tree and examined the distribution of dates of this increase; median and 95% HPD estimates were calculated for each clone.

Prior to conducting Bayesian phylogeographic analyses, the association index was computed to find evidence of geographic clustering within clone phylogenies (*72*). Based on randomly permuting the locations 1,000 times, we identified clones for further spatiotemporal analysis where less than 5% of the randomisations had a higher association index than the non-permuted dataset. Samples from the same clone were subsampled to keep only one sequence for every cluster of genetically related samples from the same city to account for regional outbreaks. In addition, locations only containing one sample within a clone were removed from the analysis of that clone.

Asymmetric phylogeographic discrete trait reconstructions were performed using the BEAST classic 1.9.0 package of BEAST 2.6.6 (*29*). The continent was used as the label for each sample. The same priors were used as above including an informed log-normal substitution rate prior and Bayesian Skyline prior on the population size. Additionally, an exponential prior was employed for the rate of lineage movements with mean 1. The relative rates of migration between continent pairs were modelled with a gamma distribution with alpha and beta set to 1. To assess the robustness of our approach in the light of overrepresentation of certain continents, we subsampled the sequences of the most frequent continent so that the two most frequent continents were equally abundant. Subsampling was repeated five times and results compared between subsamples. Spread 0.9.7.1 (*73*) was used to identify migration routes between continents. We only reported routes that had a Bayes factor of at least 3 in both the full sample and in at least four out of five subsamples.

Pan-genome analysis

Genomes were assembled from short-read data using the Velvet or SPAdes assembler using the Assembly Improvement pipeline (*95*–*97*). Where several assemblies were available, the assembly with the fewest contigs was selected. Where the number of contigs exceeded 500 or less than 3000 genes were predicted (as determined by *panaroo-qc),* Shovill 1.1.0 (https://github.com/tseemann/shovill) was used to re-assemble the genome. Panaroo 1.2.8 with -

--clean-mode moderate (*39*) was used to cluster the gene sequences from all samples into gene families and infer a graphical pan-genome (for every patient-clone combinations the earliest sample was used).

Parsimony reconstruction was used to infer the presence or absence of each gene in the common ancestor at the root of each clone tree. We then picked the genome that was most similar to the ancestral genome in terms of gene presence and absence as the ancestral epidemic clone representative. The genome graph was then reduced to a random subset of the sporadic clones (N = 50) and epidemic clone representatives (N = 21). The graph was then ordered against the genome of *Pseudomonas aeruginosa PAO1* and any long-range connections (>100 genes distance) were cut as described previously (*38*). yFiles 1.1.1 (https://www.yworks.com/products/yfiles) was then used to infer a layout in Cytoscape 3.8.2 (*98*) for visualisation. An outgroup-rooted tree was inferred based on the clone representative genomes using FastTree (2.1.10) employing the PA7-type isolate strain AZPAE14941 as the outgroup. Using the reduced pan-genome, parsimony ancestral character state reconstruction was used to infer gene gains and losses on the branches of the rooted tree leading to the ancestral epidemic and sporadic clones. Co-gained genes were then aggregated into events based on the phylogenetic context. EggNog-mapper 2.1.6 (*99*) was used to annotate the gene family of each gene in each event using the representative sequence for the corresponding gene families from Panaroo.

A Fisher exact test was conducted to compare the number of genes gained and annotated with a specific COG category. Multiple-testing correction was applied to account for the number of tests (the number of COG categories) using the Benjamini-Hochberg method to control the false discovery rate at 10%.

THP1 F508del cell line

Isogenic F508del homozygous THP1 cells were created as follows: Alt-R® CRISPR-Cas9 (S.p. HiFi Cas9 Nuclease V3; crRNA; and tracrRNA), and ssODN HDR donor template (Ultramer® DNA Oligos) were purchased from Integrated DNA Technologies (IDT). Genotyping PCR primers were obtained from Eurofins Genomics.

Ribonucleoparticles (RNP) assembly and electroporation of the gene editing reagents were performed as previously described (*100*). Briefly, the 100uM stocks of crRNA and tracrRNA were combined at equimolar concentrations for a final duplex concentration of 44 μM, incubated at 95 ^{o}C for 5 min, and then allowed to cool down to room temperature. The crRNA:tracrRNA complex was combined at a 1:1.2 molar ratio with Cas9 nuclease protein and incubated for 20 min to form the RNP complex. THP-1 cells were electroporated using the Neon™ Transfection System (Thermo Fisher Scientific) and the Neon™ Transfection System 10 μl kit. For each electroporation reaction, 100,000 cells were resuspended in 5 μl of Buffer R, mixed with 7 μl of RNP complex, and electroporated with 300 ng ssODN HDR donor template. For the co-transfection of RNP and ssODN HDR donor template, ssODN HDR donor was added to the RNP:cells mixture before the electroporation step. The cells were seeded in a 24-well plate and 72 h after electroporation editing efficiency was evaluated.

After electroporation, cells were harvested for genomic DNA (gDNA) extraction using the DNeasy Blood and Tissue Kit (Qiagen). The target site was amplified using Q5 Hot Start High-Fidelity 2x Master Mix (New England Biolabs), PCR products were purified, and Sanger sequenced (Eurofins Genomics). The gene editing events were analysed using the Sanger sequencing files from unedited and edited cells as input into the Inference of CRISPR Edits (ICE) web tool (2019, v2.0. Synthego).

THP1 infection assay

Wild type (WT) and F508del THP-1 cells were cultured in RPMI-1640 (Invitrogen) medium supplemented with 10% heat-inactivated fetal bovine serum (Invitrogen), 100U/mL penicillin and 100ug/mL streptomycin (Sigma), in 5% CO_2 humidified atmosphere at 37 °C. THP-1 monocytes were seeded at 200,000 cells/mL in 24 well tissue culture plates (Corning), and differentiated into macrophages in the presence of 20 ng/mL phorbol 12-myristate 13-acetate (PMA, Sigma–Aldrich) for 48 hours followed by a recovery period of 24 hours in serum-supplemented RPMI-1640 medium without PMA (as previously described (*75*)). Cell differentiation was verified by detection of morphology changes using light microscopy.

Pooled clinical isolates of *P. aeruginosa* were cultured in low-salt LB (Thermo Fisher) at 37 °C with aeration at 200rpm overnight, resuspended in RPMI-1640 supplemented with serum and then added to differentiated WT or F508del THP-1 cells at a multiplicity of infection (MOI) of 1:1, centrifuged at 1800rpm for 3 minutes, and then incubated at 37 °C for 1 hour before the supernatant was removed and cells were lysed (using 2% Saponin) at 1h time point or incubated in fresh media for further time points (2h or 4h) before supernatant removal and cell lysis. DNA was extracted using the QiaAmp DNA mini kit (Qiagen).

Strain abundance was quantified using mSWEEP 1.4.0 sequence-based deconvolution method (*76*). First, we built an index using the 51 input genomes using the *build_index* command. The reads for every sample were then pseudoaligned to the index using the *pseudoalign* command. Finally, the abundances of the strains in our samples were estimated by running the *mSWEEP* command. Strains with less than 1% abundance at the 1h time point were excluded from the analysis. A difference in the abundance of ST27 strains vs ST111 and ST235 strains at the 4h relative to the 1h timepoint was assessed using a two-tailed t-test.

Mutant *ΔdksA1-2* and complemented *ΔdksA1-2::DksA1 P. aeruginosa* were cultured in Luria-Bertani medium (LB, Thermo Fisher) supplemented with 100ug/ml tetracycline, and PAO1 was incubated in LB only at 37 °C with aeration at 200rpm overnight.

Bacterial suspensions in RPMI-1640 supplemented with serum were added to the differentiated WT and F508del THP-1 cells at a multiplicity of infection (MOI) of 1:1, centrifuged at 1800rpm for 3 minutes and then incubated at 37 °C for 1 hour. THP-1 cells were lysed at timepoints 1 hour and 4 hours using 2% Saponin (Sigma) and colony forming units were calculated by plating the lysates on LB agar.

Transcriptomic analysis

Gene expression data for clinical *P. aeruginosa* strains (and the UCBPP- PA14 wildtype control strain) was obtained as described previously (*25*). Briefly, strains were grown in LB broth at 37 °C and harvested at late log phase (OD600 = 2). Sequencing was done on an Illumina HiSeq 2500. Expression data were pseudoaligned to strain-specific gene indices to produce abundance estimates using Kallisto (*77*) and orthologous genes present in 99% of strains, as determined by *Panaroo* as above, were retained for downstream analysis. Expression levels for missing genes were imputed with 0 values. Abundance estimates were scaled to the median length of each ortholog across strains. Length-scaled abundance estimates `were size-factor normalised by the median ratio method and modelled as a response to CF proportion per sequence type using a negative binomial generalised linear model (GLM) with DESeq2 (*78*). The coefficients for gene models were assessed using the Wald test, and the resulting p-values were corrected using the Benjamini-Hochberg method. Genes with adjusted p-values < 0.05 were considered to be significantly associated with CF proportion.

To assess the distribution and clustering of transcriptional diversity of strains with respect to CF proportion, we used *k*-means clustering on the principal components (PCs) of the gene

expression data. We performed clustering using a range of k values and chose k = 20 because it was associated with a relatively low within-cluster sum of squares (WCSS) and reasonable cluster sizes (mean cluster size = 12). We ran the algorithm 100 times with different initial centroids and used the initial centroid placement which minimised the WCSS. We then calculated the mean standard deviation (σ) of the CF proportion by cluster (mean σ = 0.135). Next, we randomly sampled the CF proportions per strain and re-calculated the mean σ each time, repeating this process 10,000 times in total. An empirical P value was calculated as the fraction of random samples with mean σ at least as low as that observed with our actual data. Since no random samples had mean σ as low as our actual observed value (smallest mean σ = 0.14), the empirical P is $< 1 \times 10^{-4}$.

Zebrafish infection experiments

Zebrafish lines: Experimental procedures were performed using the wild type AB line and the knockout cftrsh540 mutant (*46*). Macrophage activity was evaluated using the Tg(mpeg1:mcherry-F)ump2 line harboring red macrophages (*47*). For zebrafish anesthesia procedures, larvae are immersed in a 0.168 mg/mL Tricaine (Sigma-Aldrich) solution in fish water. When required, larvae were cryo-anesthetized by incubation on ice for 10 min then euthanized using an overdose of Tricaine (0.500 mg/mL).

Morpholino injection: Morpholino were purchased from Gene Tools. The morpholino for cftr knockdown (5'-GACACATTTTGGACACTCACACCAA-3') were prepared and injected into one-cell-stage as previously described (*79*). A standard morpholino control (5'-CCTCTTACCTCAGTTACAATTTATA-3') was used as a negative control.

Generation of fluorescent Pseudomonas aeruginosa strains: Plasmids were obtained from Addgene. *P. aeruginosa* GFP+ strains were obtained by triparental mating, using an E. coli strain containing the plasmid pRK2013 (*101*) as helper strains and an E. coli containing pMF230 (*102*) as the donor strain. GFP+ colonies were selected on LB agar plates complemented with carbenicillin 300 µg/ml and 5 µg/ml tetracycline (LBCarb/Tet) (Sigma-Aldrich).

Pseudomonas aeruginosa inoculates and microinjection into zebrafish embryos and larvae: P. aeruginosa expressing GFP were grown using LBCarb/Tet medium. To prepare *P. aeruginosa* inoculates, 1 ml of LBCarb/Tet medium was inoculated with a single colony of bacteria and incubated at 37°C overnight with shaking. 50 µl of this overnight culture was then added to 5 ml of LBCarb medium and incubated at 37°C with shaking to mid-log phase (OD600 0.6-0.8). *P. aeruginosa* were next harvested by centrifugation and resuspended in a volume of PBS (GibcoTM, Thermo Fisher ScientificTM). Then bacterial suspensions were homogenized with a 26-gauge needle and resuspended at an OD_{600} of 1 in PBS, then kept on ice until zebrafish infection challenges. Systemic infections were carried out by the microinjection of P. aeruginosa into the caudal vein of 30 hours post-fertilization (hpf) zebrafish embryos as described earlier (*80*) with some modifications. Briefly, tricaine-anaesthetized embryos were infected individually with 1-2 nl *P. aeruginosa* inoculate (≈250-350 colony forming units (CFU)). Inocula were checked a posteriori by microinjection onto LB agar plates. Survival post *P. aeruginosa* infection was assessed daily by counting dead embryos (no heartbeat) up to 6 days.

Zebrafish whole bacterial burden analysis: Growth of *P. aeruginosa in vivo* was assessed by CFU analysis at 1 day post-infection (dpi). To determine CFU, groups of five larvae were anesthetized, collected, euthanized and transferred individually into microfuge tubes with 1% Triton X-100 (Merck Millipore) in PBS. Larvae were then mechanically homogenized using a micro pestle (Eppendorf), washed to removed Triton and resuspended in PBS. 10-fold serial dilutions were plated on LB agar plates and incubated overnight at 37°C prior to CFU counts.

Zebrafish macrophage activity and Intra-macrophage bacterial burden analysis: Macrophage response was elicited through infection of *P. aeruginosa* expressing GFP into the muscle

compartment of anesthetized Tg(mpeg1:mcherry-F)ump2 larvae at 3 dpf as previously described (*79*, *81*). Macrophage chemotaxis and phagocytosis was evaluated by assessing the number of cells and/or infected cells at infection sites using confocal microscopy. Macrophage chemotaxis was determined at 2 hours post-infection (hpi), and phagocytosis at 4 hpi. Intra-macrophage P. aeruginosa loads, which reflects the bacterial killing, were assessed by quantifying the volume of bacteria inside macrophages at 6 hpi using confocal microscopy.

Zebrafish Microscopy, Image Processing and Analysis: Bright-field and fluorescence microscopy of infected embryos/larvae were performed using an Olympus MVX10 epifluorescent microscope equipped with a X-Cite120Q (Lumen Dynamics) 120-W mercury light source. Images were acquired with a digital color camera (Sony MiC5 Pro) and processed using CellSens (Olympus). Confocal fluorescence microscopy was performed using an ANDOR CSU-W1 confocal spinning disk on an inverted NIKON microscope (Ti Eclipse) and 20x/NA 0.75 air and 40x/NA 1.15 water objectives. Images were acquired with a W&B Zyla 4.2 camera (ANDOR) and processed using IQ3 3.6.5 software (ANDOR).

Overlays of fluorescent and DIC images and 2D reconstructions of image stacks were assembled using FIJI freeware. 3D reconstitution was produced using Imaris 9.0 software (Bitplane).

Mutational burden analysis

PAO1-rooted clones trees were used a input to Treetime 0.8.1 (*82*) to infer a maximum likelihood ancestral character state reconstruction of every nucleotide position. We then implemented a pipeline (https://github.com/aweimann/PhyloEffects) to identify single nucleotide changes and annotate variant effect in their phylogenetic context using the gene annotation from Pseudomonas.com (PAO1 107) (*55*) and the ancestral character state reconstructions. Parsimony ancestral character state reconstruction was used to infer ancestral insertions and deletions. Only unique insertions or deletions were kept. SNPeff version 4.3.1 (*48*) was used to annotate the effect of indels on gene function.

We performed a mutational burden test assuming a Poisson distribution of the mutational burden per gene. We compared the observed number of non-synonymous SNPs and indels within a gene across all clones with the expected number of variants in that gene based on the total number of variants across all clones. *Panaroo* (*39*) was used to estimate the prevalence of every orthologous gene family and infer an adjusted number of expected variants. Multiple-testing correction was used to account for the number of tests (namely the number of genes in the reference genome *PAO1*) using the Benjamini-Hochberg method (*103*) to control the false discovery rate at 5%. The 224 genes passing the adjusted p-value threshold were used to query the STRING 11.5 database (*56*) of protein-protein interaction. STRING reports the statistical significance of the number of interactions found among the input set of genes compared with the number of expected interactions by chance.

Pathoadaptive genes were assigned to 17 functional categories based on the gene products description on Pseudomonas.com (*55*) (Figure S1). Genes across the PAO1 genome were also stratified by whether they had an assigned gene product name. A Fisher exact test was used to compare the number of assigned with the number of unassigned genes among pathoadaptive genes and non-hits.

Genotype-TF regulon analysis

To assess the association between genetic variants and the expression of transcription factor (TF) regulons, gene expression data from (*25*) were pseudoaligned to strain-specific gene sets using Kallisto (*77*). Sample-specific size scaling factors for normalisation were derived from core gene expression using DESeq2 (*78*) and size-scaled counts were log_2 transformed for variance stabilisation. We performed binary scoring of TFs based on the presence (1) or absence (0) of

missense, nonsense or INDEL variants. The normalised expression levels of TF regulons were compared between strains with and without genetic variants using Welch's two-sample *t*-tests. P-values from *t*-tests were adjusted using the Benjamini-Hochberg method.

Phenotyping of pathoadaptive gene transposon mutants

PAO1 mutants with transposon insertions in 154 pathoadaptive genes (selected from the Manoil library (*86*) were arrayed in 96 well plates containing 120ul of no-salt LB broth. Plates were incubated overnight at 37C in a static incubator, after which, DMSO was added to a final concentration of 5% and plates were stored at -80°C. For all assays performed on arrayed mutants, the inoculum was in the form of a fresh over-night culture from the frozen stock plate. All agar plate-based phenotyping assays were performed in duplicate Plus Plates (Singer Instruments) that were inoculated using the ROTOR and 96-Long re-pads (Singer Instruments). Results from all agar plate assays were imaged using the Phenobooth Imager (Singer Instruments).

Swimming motility: the inoculum was stabbed through the agar until the pins contacted the base of the plate resulting in a column of bacteria within the agar layer. Agar plates were incubated at 37°C for between 4 and 6 hours for the formation of an opaque halo surrounding the point of inoculation which was indicative of swimming motility.

Twitching motility: Plus Plates were filled with 12.5ml of low salt LB with 1.5% agar to create a thin, even layer of agar on the bottom of the plate. Inoculum was stabbed through the agar layer into the plastic-agar interface by increasing the pressure applied by the pins on the ROTOR to 100%. Plates were incubated at 37°C for 6 hours under humid conditions, after which they were treated for 30 minutes with chilled TM developer solution (40% methanol and 10% acetic acid) at room temperature. Liquid was decanted from the plates and the agar layer was carefully removed to expose the adherent growth on the plastic of the plate. Plates were allowed to air dry which caused the halo to turn white and become more visible and easier to visualise using the Phenobooth.

Siderophore production: Chrome-azurol (CAS) agar for assaying siderophore production was produced as previously (*104*). To prepare the deferrated cas-amino acid solution, 10g cas-amino acids were dissolved in 100ml of sterile distilled water, and 3g 8-Hydroxyquinilone were dissolved in 100ml chloroform, the 2 mixtures were combined and shaken for around 10 minutes. After shaking, the mixture was transferred to a separating funnel and allowed to rest for 10 minutes. The bottom fraction was discarded, and the top layer collected in a fresh bottle containing 100ml of fresh chloroform. The process was repeated, and the final eluate was left to rest overnight on the bench with the lid loosely attached to allow residual chloroform to evaporate. The deferrated cas-amino acids were then filter sterilised and stored at 4°C. All glassware used for the preparation of CAS agar was rinsed once with 6M HCl followed by washing three times with sterile de-ionised water before use, to eliminate interference. Before the addition of agar, the medium was adjusted to pH 6.8 and after autoclaving, the agar was allowed to cool down to 50 °C before the addition of blue dye, glucose and deferrated cas amino acids. CAS agar plates were inoculated with overnight liquid culture and incubated at 37°C overnight before imaging using the Phenobooth. Formation of an orange halo around the point of inoculation indicated siderophore activity.

Caseinase activity: Skim milk agar was prepared by combining sterile, molten tryptic soy agar (40g dissolved in 1 litre of water) with sterile 10% marvel milk (50ml per litre of agar). Plates were inoculated with overnight culture and incubated at 37°C overnight after which they were observed for the formation of a clear halo.

Gelatinase activity: Medium consisting of nutrient broth (made according to the manufacturer instructions), 1.5% agar and 3% porcine gelatine was dispensed into plus plates. The temperature was maintained at 55°C and the medium was stirred continuously using a magnetic stirrer to ensure a uniform distribution of gelatine throughout the medium. Agar plates were inoculated with overnight culture and incubated at 37°C for 6-8 hours after which they were stored at 4°C overnight to enable the halo to develop fully. After incubation at 4°C, plates were flooded with a solution of 4.1M ammonium sulphate and incubated at room temperature to reveal clear halos that are the result of gelatine degradation by gelatinase. After 30 minutes, the liquid was discarded, and the plates were imaged.

Rhamnolipid production: Agar was prepared containing Trizma base (14.5g), peptone (10g), glucose (5g), NH_4Cl (0.7g), KCl (1.5g), $MgSO_4$ (0.39g), ddH2O (1l), adjusted to pH 7.4. before the addition of agar (15g) and autoclaving. The agar was then allowed to cool to approximately 50°C before it was supplemented with 10ml of sterile CTAB solution and 10ml of sterile methylene blue. Agar plates were inoculated with fresh overnight culture and incubated overnight at 37°C. On the second day, plates were transferred to 4°C for a further 48 hours to allow halos to develop.

Impact of amino acid changes on protein stability and structural analysis

All amino acid changes were analysed with SIFT 4G 6.2.1 (*49*) using the UniProt90 database (*85*). Since *Pseudomonas* does not have a pre-computed database, we built our own SIFT database using the genome and gene annotations from Pseudomonas.com. All non-synonymous mutations were then annotated with the *annotator* command. FoldX 5 (*50*) was used to predict the difference of total energy between the variant and wild type allele for every variant. AlphaFold models were downloaded from Uniprot and the *RepairPDB* command of FoldX was used to repair residues with bad torsion angles or total energy and van der Walls' clashes (*105*). The repaired PDBs were then used as input to the *BuildModel* FoldX command. FoldX and SIFT scores were then averaged per gene/protein and a two-tailed t-test was used to compare the average scores between mutational burden test hits and non-hits.

Mutational frequencies were mapped on the structural models of the identified hotspot genes (*algU*, *ladS*, *pcnA* and *betT2*) in *P. aeruginosa* using the Chimera molecular modelling package (*84*). Coordinates for AlgU were downloaded from the Protein Data Bank. Predicted AlphaFold models (*105*) were downloaded from UniProt for *ladS, pcnA* and *betT2*.

Transmission and host selectivity of pathoadaptive mutations

To assess the transmissibility of pathoadaptive changes, the number of mutations that had been observed in at least two isolates (from different patients) was compared with mutations that had only been observed once using a Fisher exact test. TopGO 2.4.6 was used for functional enrichment analysis of the host-specific Gene Ontology biological pathway annotation compare to the background (*57*) enrichment. Gene Ontology biological process annotations were downloaded for the PAO1 strain from *Pseudomonas.com* (*55*).

Mutations in pathoadaptative genes were stratified by the (ancestral) infection type (CF or non-CF) of every branch based on outgroup-rooted rooted clone trees. To assess host-specific pathoadapdation, the number of CF vs non-CF mutations were compared using a Fisher exact test. Mutations on branches with non-concordant ancestral infection types were discarded. Multiple-testing correction to account for the number of tests (the number of pathoadaptive genes) was achieved using the Benjamini-Hochberg method to control the false discovery rate at 10%.

Pathoadaptive trajectories

Trajectories were inferred as the sequence of mutations in pathoadaptive genes since the emergence of the clone ancestor (random assignment was used where several mutations

coincided on one branch) as implied by the PAO1-rooted tree stratified by cystic fibrosis (CF) and non-CF infection types. Mutation frequencies were position normalised and the frequency plots of the 40 genes with the lowest p-value from the mutational-burden test were manually assigned into five groups of genes with similar frequency curve shapes. Trendlines were generated by locally-weighted smoothing.

Transmission analysis
We established a relatedness cut-off to define potential transmission links using pairwise SNP differences between pairs of isolate genomes from the same patient (n = 81 patients). Potential hypermutators were removed prior to this analysis as described above. We defined a patient-level cut-off as the 95th percentile of the distribution of within-host SNP distances for every patient as described previously (*75*). A global cut-off was derived as the 95th percentile of the distribution of all patient cut-offs. We then identified potential transmission events as isolates from the same clone sampled from different patients that differed by 26 SNPs or fewer. Transmission clusters were annotated and laid out using Cytoscape.

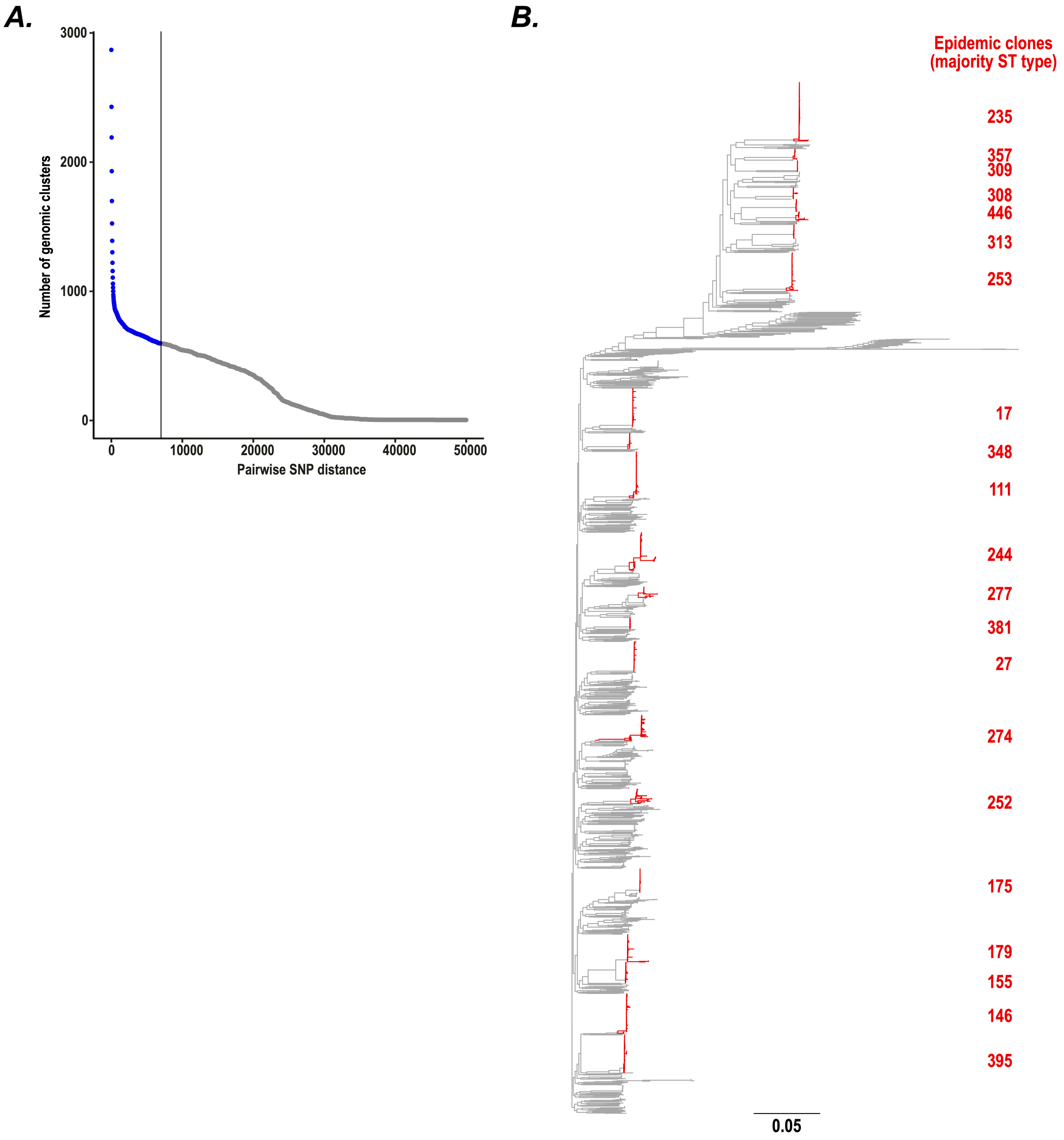

Figure S1. (A) Relationship between pairwise short nucleotide polymorphism (SNP) distance threshold and implied number of genomic clusters. Isolates genomes were clustered based on their pairwise SNP distance using the unweighted pair group method with arithmetic means (UPGMA). UPGMA infers a sample dendrogram which is then separated into cluster by applying a discrete SNP distance threshold. A vertical black line at 7000 SNPs denotes the threshold that was chosen to define the genomic clusters (clones; *blue*) in our dataset. **(B)** Maximum likelihood phylogenetic tree generated from genomes of all study isolates (major epidemic clones labelled in red).

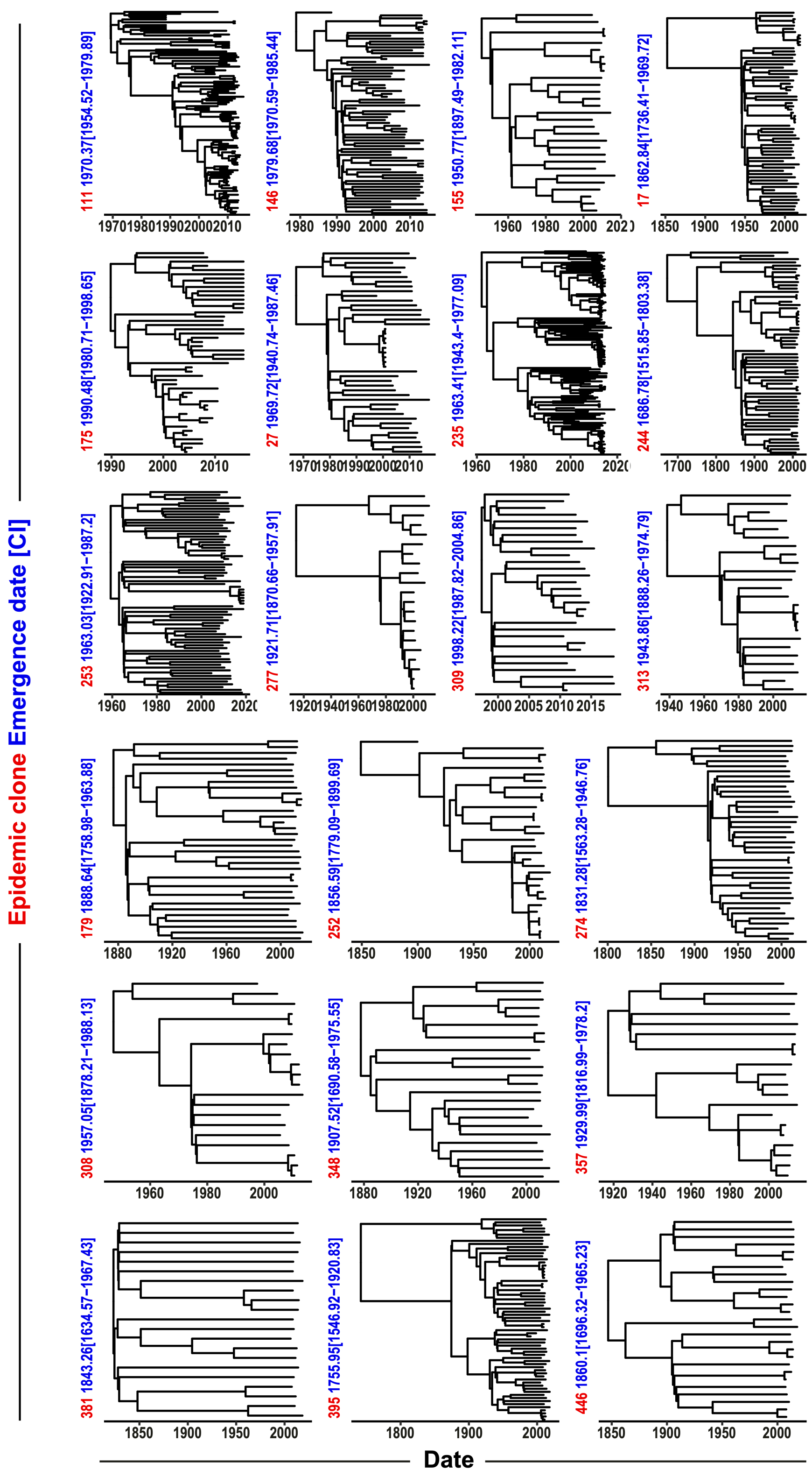

Figure S2. Emergence dates of epidemic clones. Bayesian inferred phylogenetic trees for all epidemic clones with mean and 90% highest posterior density interval estimates of the emergence date.

Epidemic clones (Majority ST)

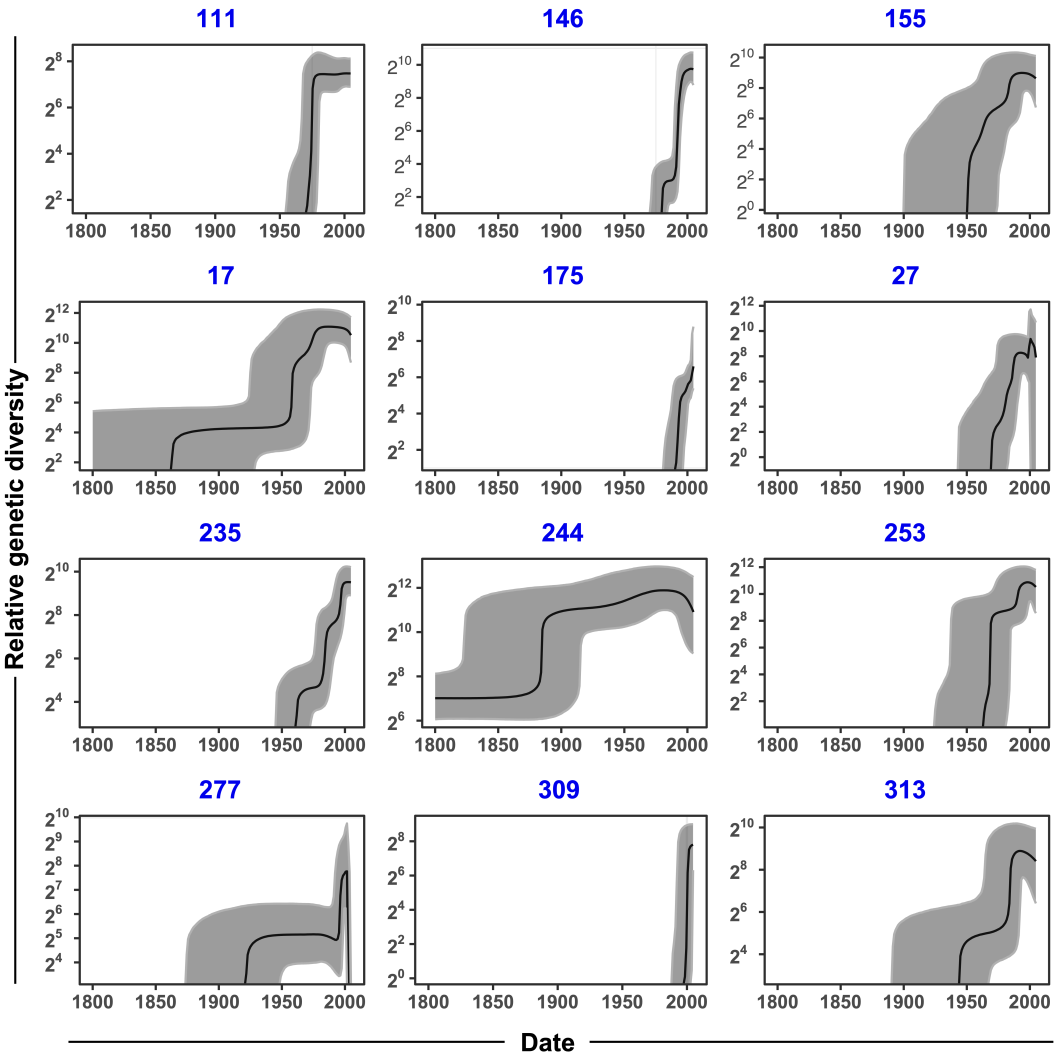

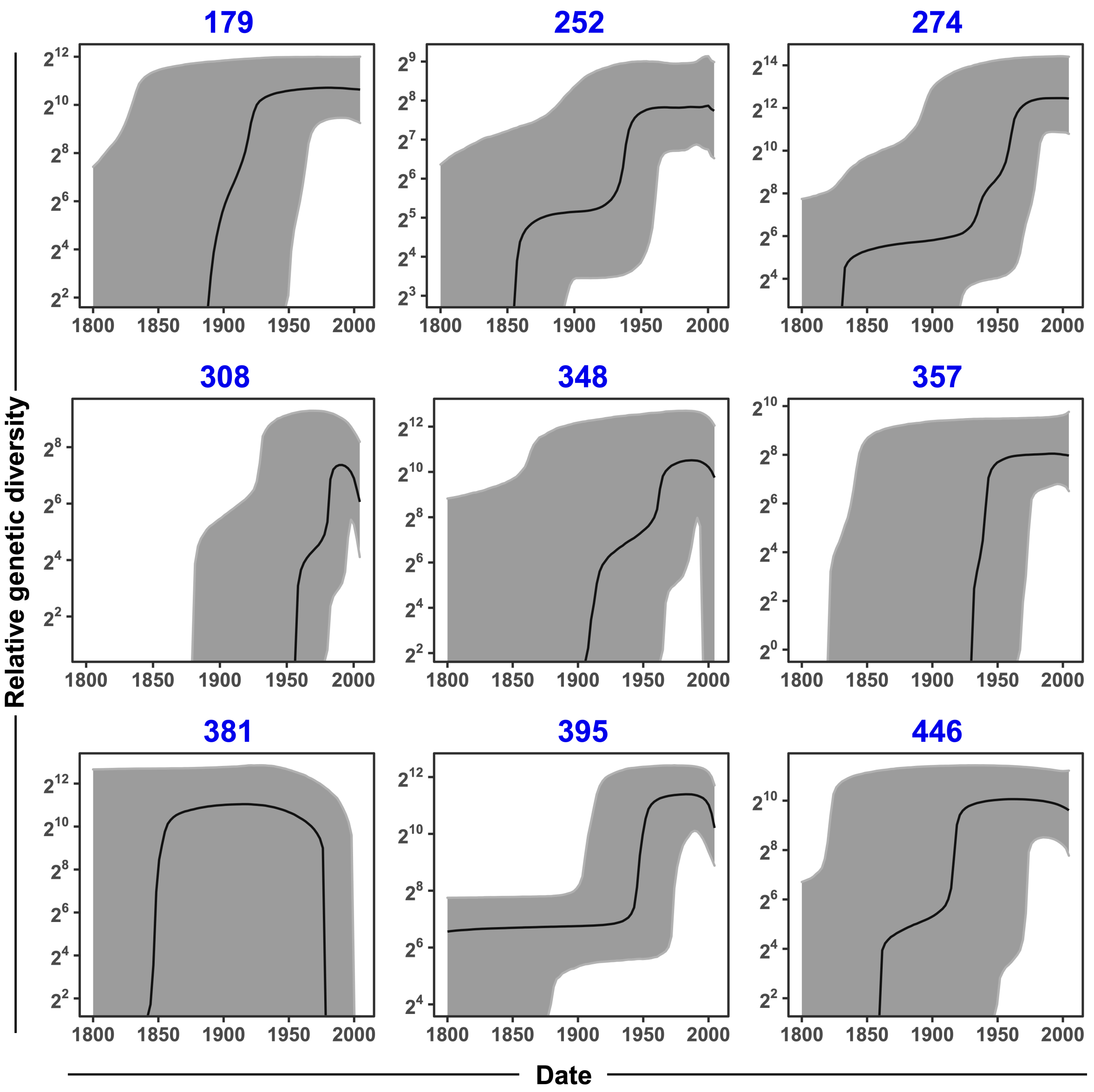

Figure S3. Population expansion of epidemic clones. Relative genetic diversity estimates (as a proxy for population size) based on Bayesian Skyline plot demographic model were inferred for all epidemic clones. 90% highest posterior density intervals are shown in grey.

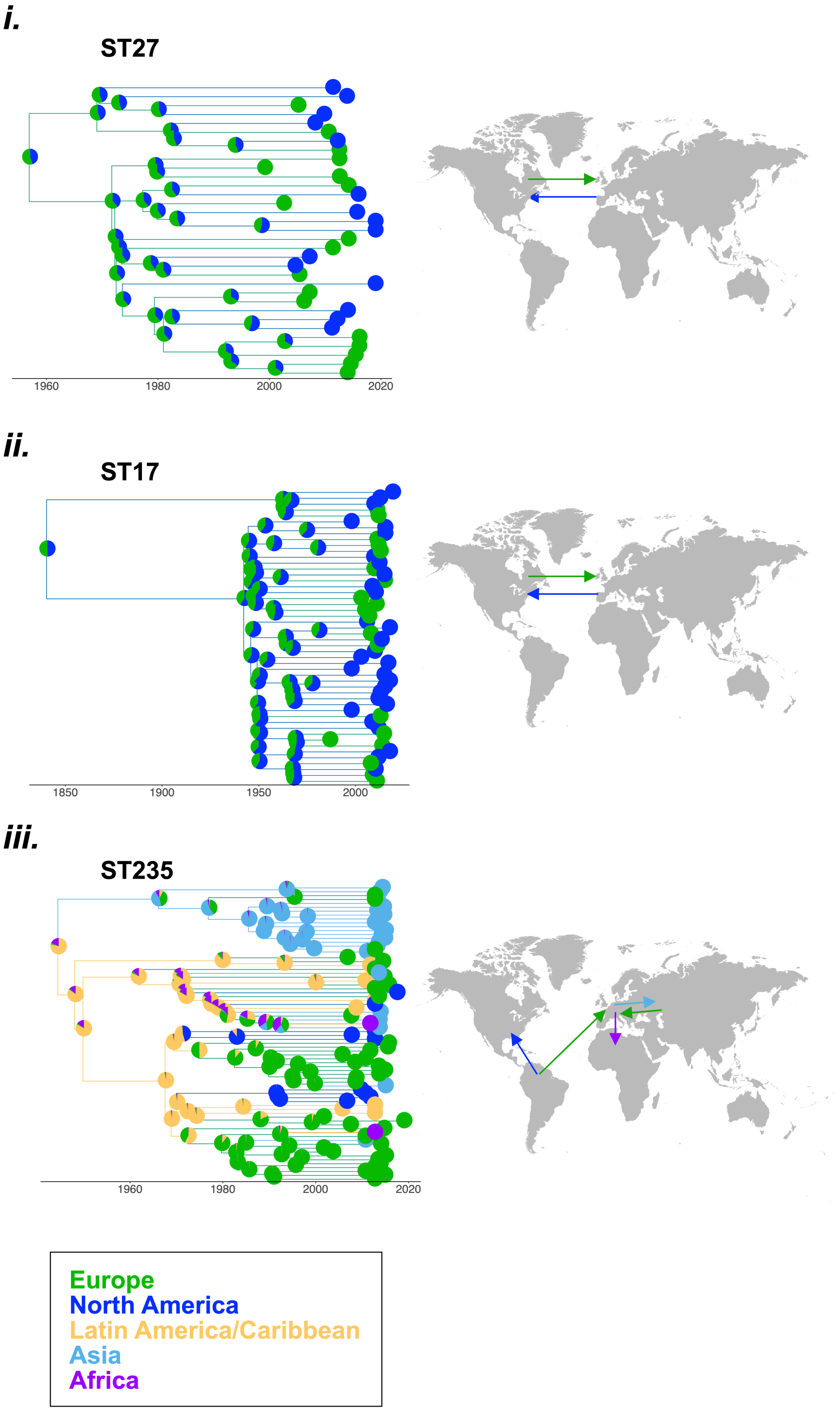

Figure S4. Phylogeography of *P. aeruginosa*. *Left* Bayesian inferred phylogeographic trees shown for three epidemic clones (i) ST27, (ii) ST17, (iii) ST235. Edges were colour-coded by the most probable continent based on the full distribution of trees (*N* = 27,000). Proportion of trees supporting individual continent are shown at nodes. *Right* Arrows show the statistically-supported direction of inter-continental migrations routes (Bayes Factor > 3). Arrows are colour-coded by the recipient continent.

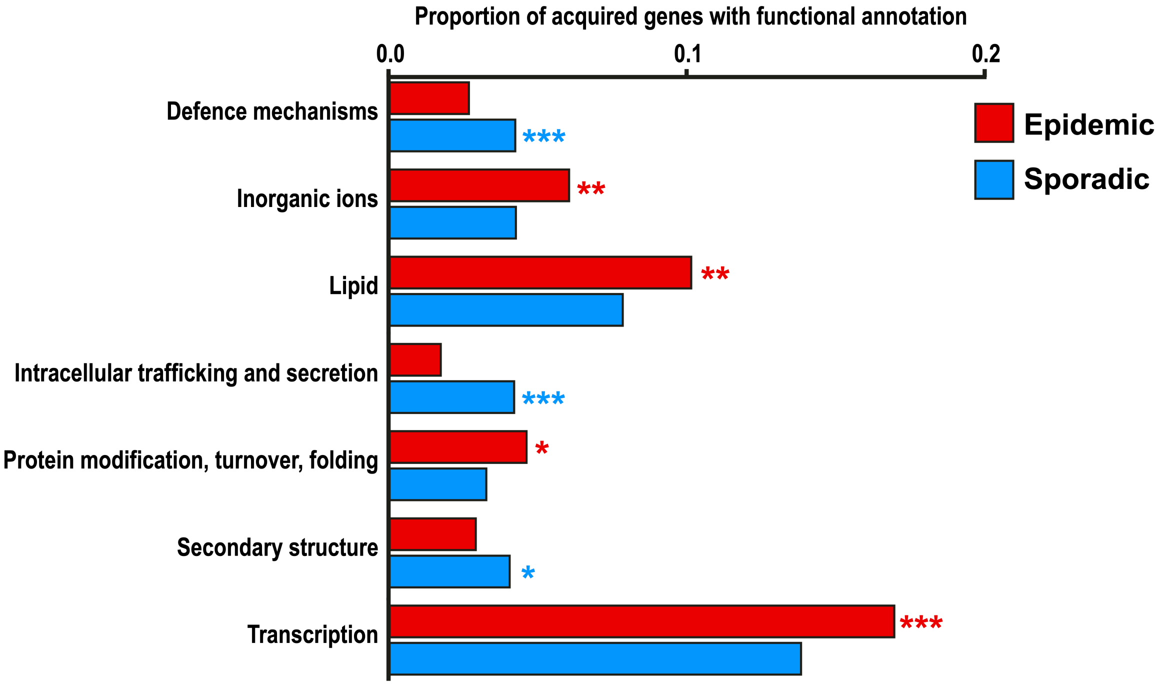

Figure S5. Gene gain events in epidemic and sporadic *P. aeruginosa* clones. Proportion of acquired genes with functional annotations (based on COG database), inferred by maximum parsimony ancestral genome reconstruction within epidemic (*red*) and sporadic (*blue*) clones. All COG categories with significantly different numbers of acquired genes between epidemic and sporadic clones are shown. * p value < 0.05; ** p value < 0.01; *** p value < 0.001.

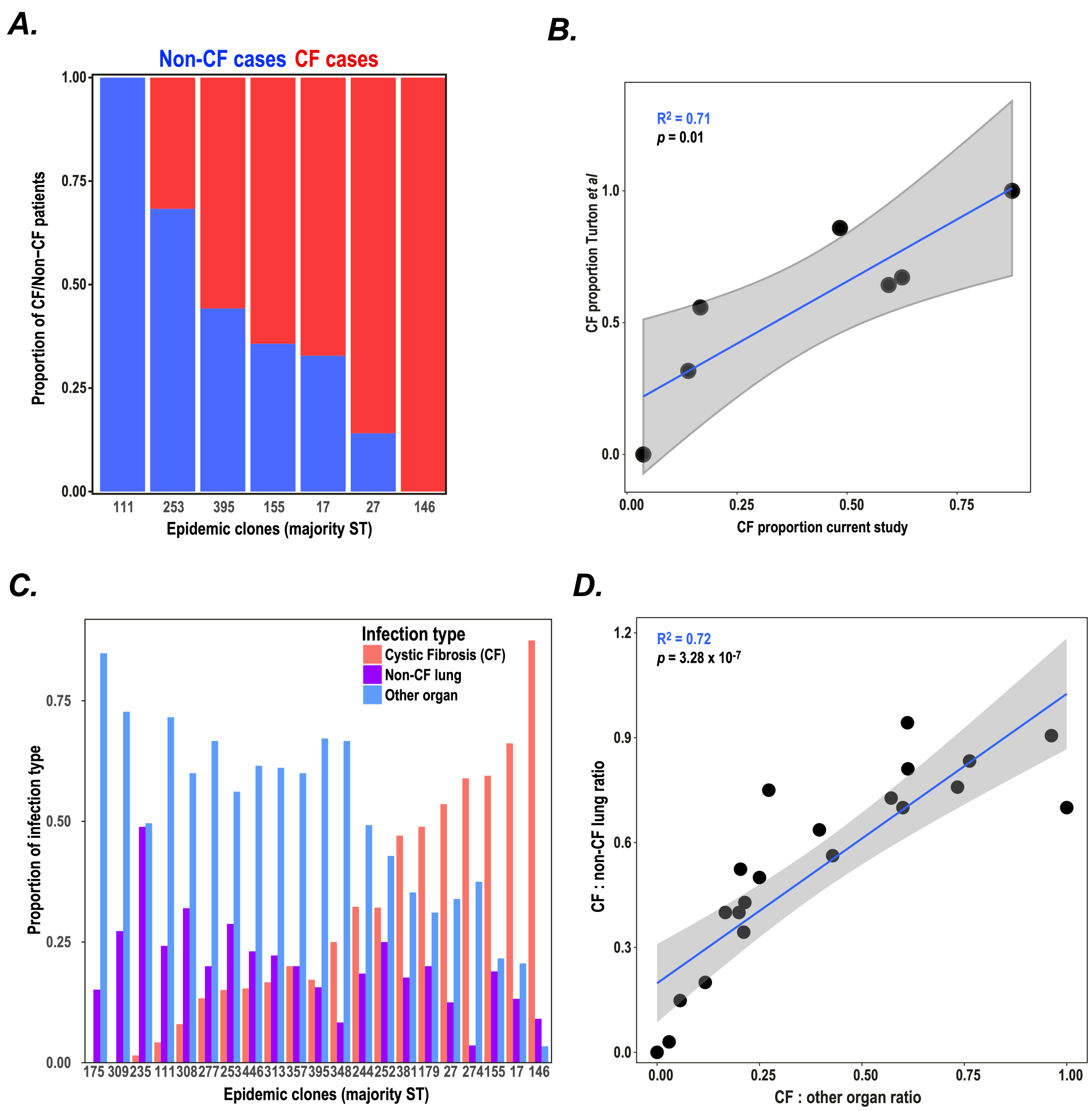

Figure S6. Further analysis of varying host preference of epidemic *P. aeruginosa* clones. (**A**) Proportion of cystic fibrosis (CF; *red*) and non-CF infections (*blue*) caused by epidemic clones also represented in our study data, analysed from data collected as part of a surveillance study with UK hospitals between 2010 and 2012 (*40*). (**B**) Comparison of CF proportion in individual epidemic clones between our study and previous surveillance study *(33)* (Adjusted $R^2 = 0.71$, $p = 0.01$, F-test). (**C**) Proportion of infections caused by epidemic clones (labelled by their majority multi-locus sequence type, ST) in patients with cystic fibrosis (CF; *red*), non-CF lung infection (*purple*), and non-lung infections (other organ; *blue*). (**D**) Comparison of ratio of CF to non-CF lung infections compared to the ratio of CF to other organ infections for major epidemic clones (Adjusted $R^2 = 0.72$, $p = 3.28 \times 10^{-7}$, F-test).

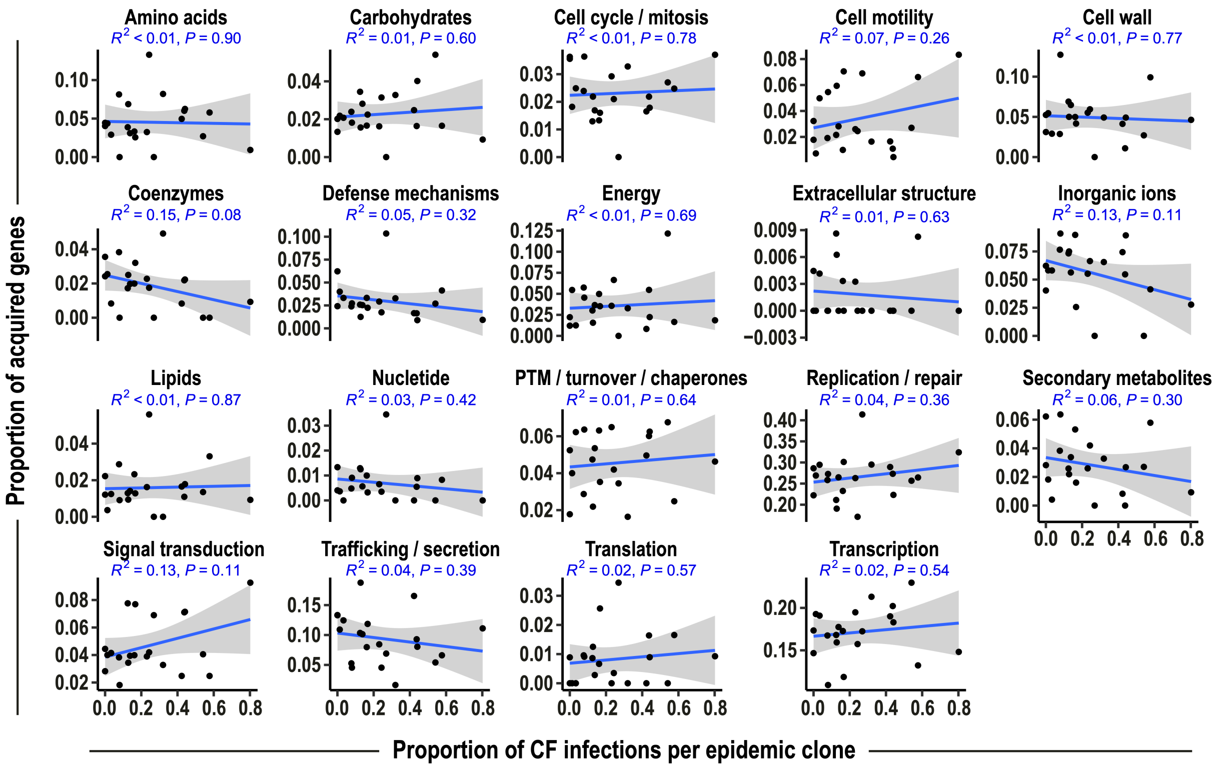

Figure S7. Gene gain events in epidemic clones across the *P. aeruginosa* CF affinity spectrum. Ancestrally-acquired genes were annotated (based on COG database) using maximum parsimony ancestral genome reconstruction across ancestral genome representatives. Each panel shows the proportion of acquired genes annotated within a specific functional category across the CF proportions of the underlying clones. Linear trendlines are shown in blue with shaded area in grey denoting the 95% confidence level interval. Adjusted R^2 and p-values are shown based on fitting a linear model for every category (F-test).

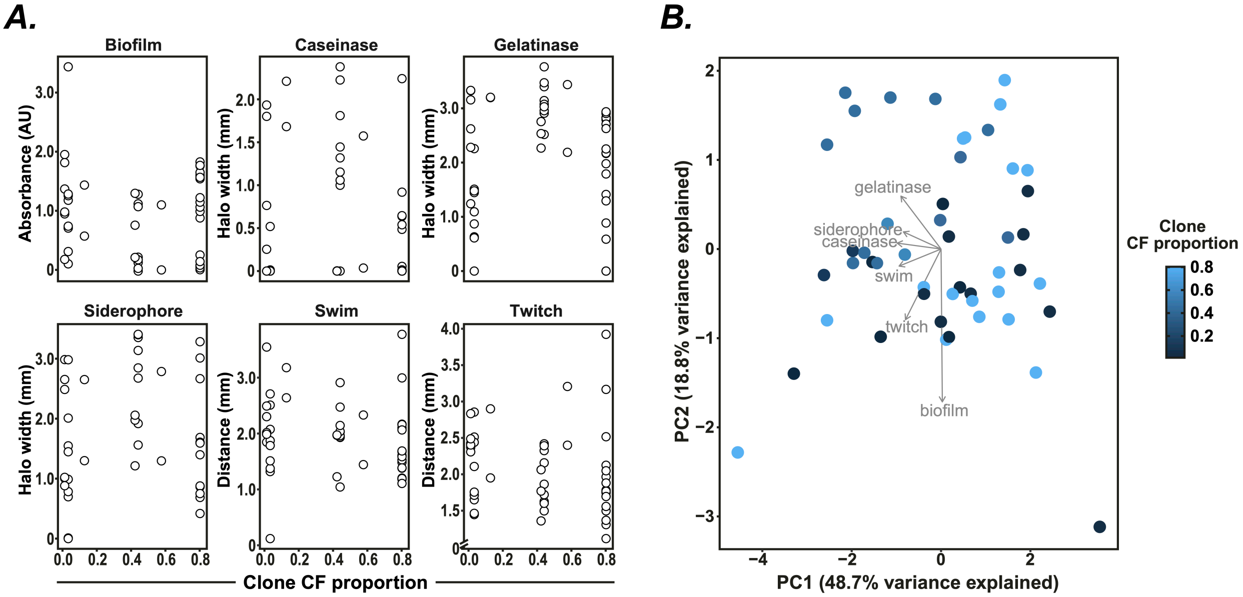

Figure S8. Multi-dimensional virulence phenotyping of *P. aeruginosa* clinical isolates. Virulence phenotypes of 49 clinical isolates as determined using a series of phenotypic assays (see Method section). (**A**) Virulence phenotype quantification for each isolate plotted against the clonal CF proportion of each clinical isolate. (**B**) Principal component (PC) analysis of virulence phenotype quantification for all tested clinical isolates, visualised using the two principal component (PC) axes explaining the most variation (variance explained annotated on the axes) based on PC analysis of all virulence factor measurements. Clonal CF proportions annotated for each isolate using blue colour scale.

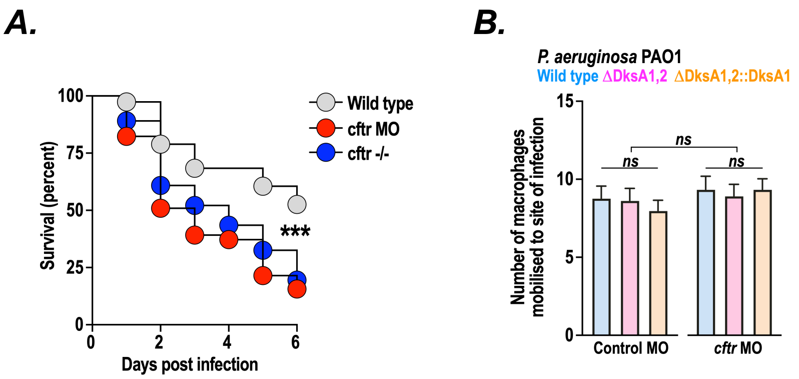

Figure S9. Modelling *P. aeruginosa* infection in zebrafish. (**A**) Wildtype (*grey*), cftr knockout (*cftr* -/-; *blue*) (*46*) and *cftr* morphant (*cftr* MO; *red*) zebrafish were intravenously infected with 250-350 colony forming units (cfu) wildtype *P. aeruginosa* PAO1. Survival analysis of *P. aeruginosa*-infected larvae. Data plotted as percentage of surviving animals over 6 days (average of 2 independent experiments; n=66). *** p < 0.001 (Mantel-Cox Log-rank test). (**B**) Control and *cftr* morphant zebrafish larvae with mCherry-labelled macrophages (*Tg(mpeg1:mcherry-F)ump2* (*47*)) were intramuscularly infected with 250-350 GFP-labelled *P. aeruginosa* PAO1 wildtype (*blue*), ΔDksA1,2 (*pink*) or PA01ΔDksA1,2::DksA1 complemennted (*yellow*) strains and the infection tracked using real-time intravital confocal microscopy. Mean ± SEM (standard error of the mean) number of macrophages mobilized to the infected muscle at 2 hours post infection (hpi). (n=14 fish; 2 independent experiments). *ns* non-significant (two-way ANOVA with Tukey's post-test).

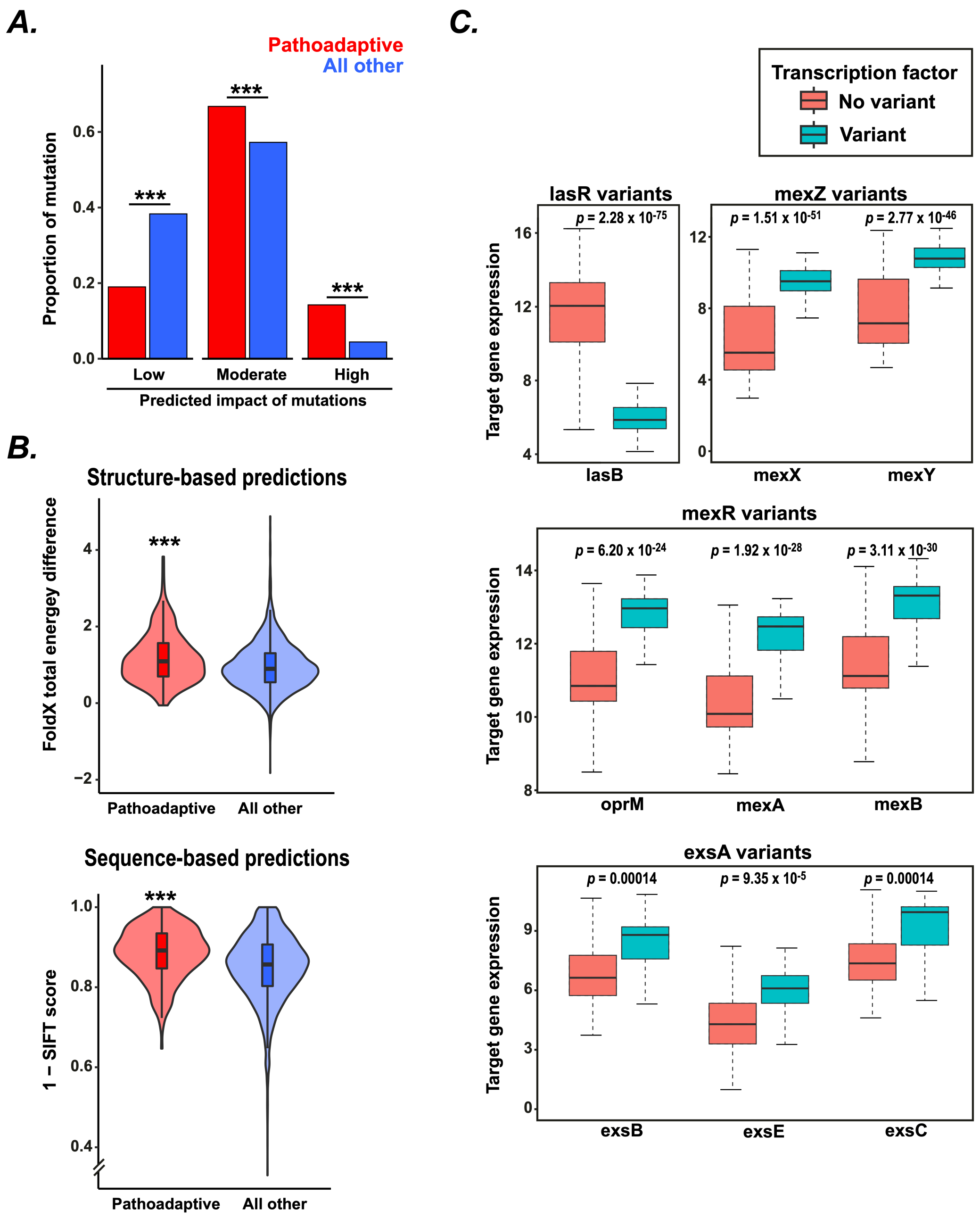

Figure S10. Predicted impact of mutations on protein function (**A**) Proportion of mutations with low, moderate, and high predicted impact in pathoadaptive (*red*) or all other (*blue*) genes (other; *blue*) estimated using SNPeff (*48*). (**B**) The estimated impact of missense mutations on protein stability predicted by (top) FoldX (*50*) (*** p = 1.34 x 10^{-6}) or (*bottom*) protein function predicted by Sorting Intolerant from Tolerant (SIFT) (*49*) analysis (*** p = 9.04 x 10^{-15}) in pathoadaptive (red) or all other (*blue*) genes. (**C**) Boxplots of pathoadaptive transcription factor regulon expression of clinical isolates with variants (*green*) or no variants (*red*) (Benjamini-Hochberg adjusted p-values from a two-sample Welch t-test shown separately for every target gene). Observed changes in target expression all consistent with the presence of loss of function mutations affecting transcription factors.

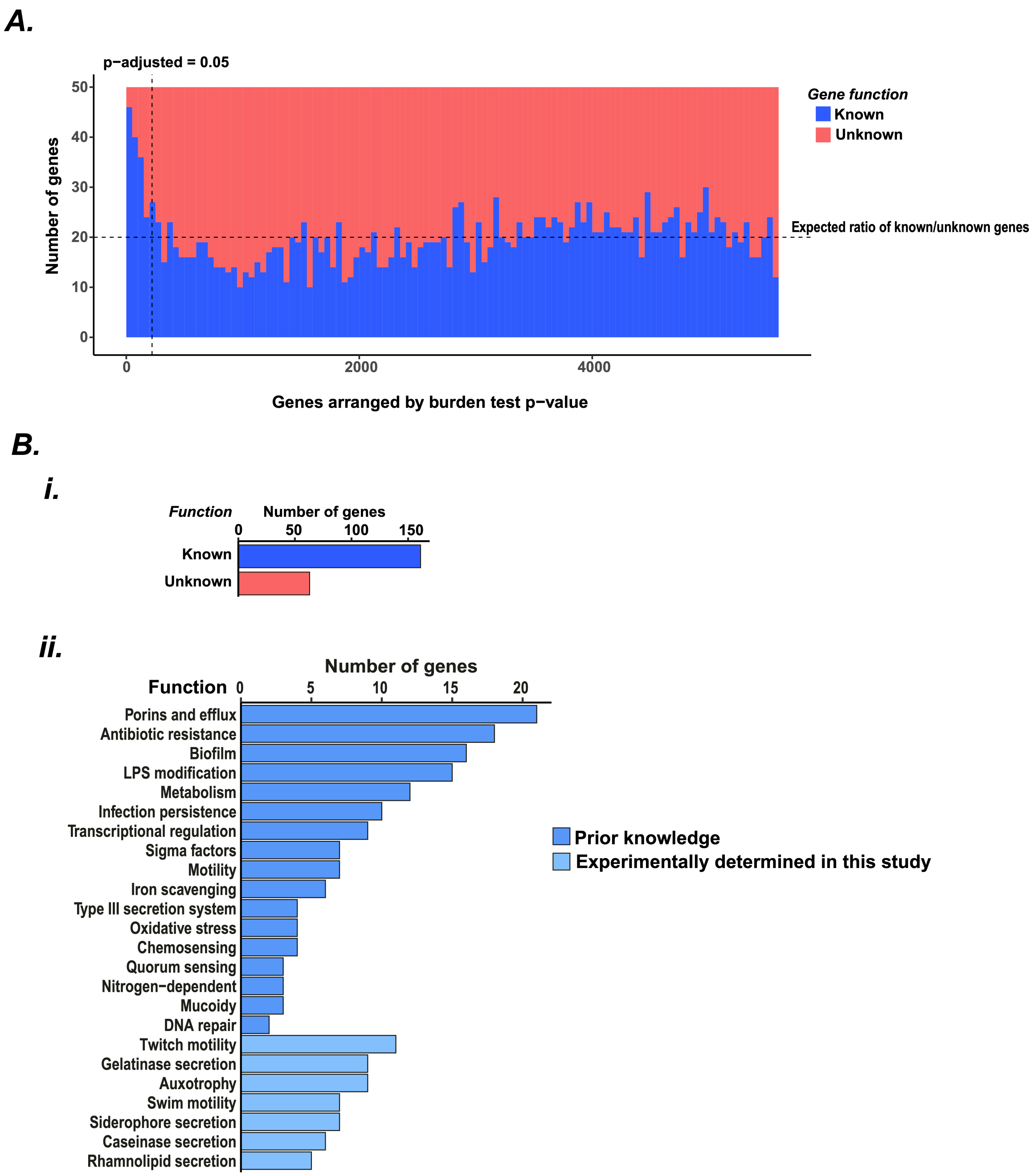

Figure S11. Functional characterisation of pathoadaptive genes. (**A**) Histogram of genes with known (*blue*) or unknown (*red*) function arranged by their adjusted p-value from a Poisson test comparing the mutational burden as expected with the observed number of mutations, with pathoadaptive genes having adjusted *p* values of <0.05. (**B**) *(i)* Fraction of pathoadaptive genes with known (*blue*) and unknown (*red*) function. (ii) Number of pathoadaptive genes with (*dark blue*) known functions (across 17 categories) based on prior knowledge or (*light blue*) experimentally determined in this study using sequence-confirmed transposon mutants from the Manoil library (*86*) representing isogenic disruption of 154 of the 224 pathoadaptive genes.

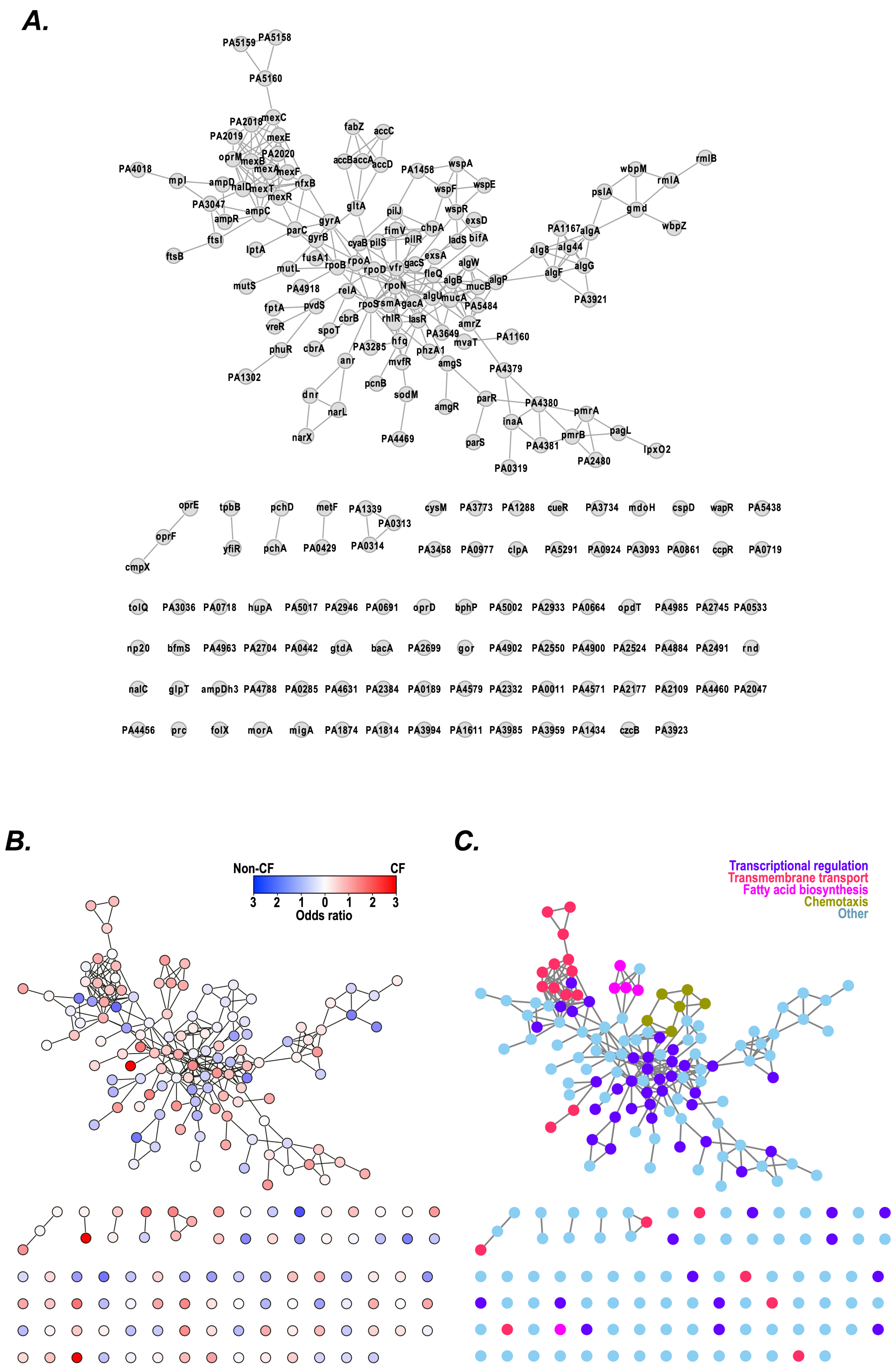

Figure S12. Protein-protein interaction networks of pathoadaptive genes. Genes are depicted as nodes and connected by an edge if their proteins are known to interact according to the STRING database (confidence > 0.7). Protein-protein interaction networks for all pathoadaptive genes (A) labelled by gene name or annotated by (B) the odds-ratio of host-specific pathoadaptation (assessed by comparing the number of CF vs non-CF mutations using a Fisher exact test (FDR = 0.1), or (C) biological process based on Gene Ontology (GO) terms.

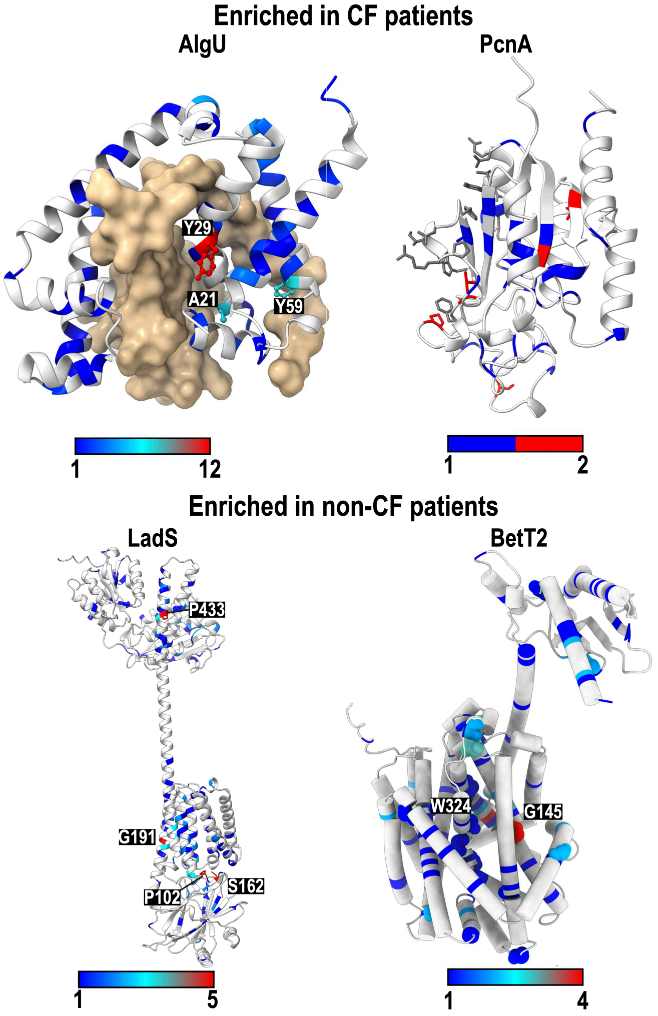

Figure S13. Examples of structural analysis of pathoadaptive mutations. Examples of structural analysis of gene products where mutations are enriched in CF patients (AlgU, PcnA) or in non-CF patients (LadS, BetT2). Frequency of mutations (information entropy) were colour-coded and mapped onto each structure (the most frequent positions were labelled). AlgU, an RNA polymerase sigma-H factor known to regulate mucoidy (*58*), is shown in complex with its negative regulator MucA as cartoon and molecular surface representations respectively with mutations occurring at the interface between these two proteins. PcnA, a putative nicotinamidase, had mutations within the protein core or at sites of protein-protein interaction. LadS, a calcium-responsive histidine kinase (*59*), acquired mutations in the N terminal (sensor) and transmembrane domains. BetT2 (*60*), a putative choline transporter, showed helix-breaking mutations within its transmembrane domain. Models based on experimentally-derived structures (for AlgU, PDB 6IN7) or AlphaFold predictions (for others).

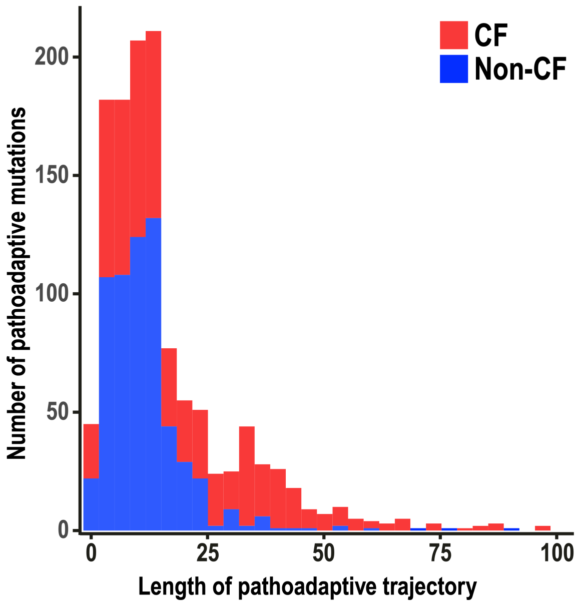

Figure S14. Evolutionary trajectory lengths in *P. aeruginosa.* Comparison of the lengths of evolutionary trajectories between isolates infecting CF (*red*) and non-CF (*blue*) individuals. Trajectories were inferred as the sequence of mutations in pathoadaptive genes since the emergence of the clone ancestor as defined by the mid-point-rooted tree stratified by CF and non-CF infection types. Evolutionary trajectories were down-sampled to contain an equal number of samples from CF and non-CF infections.

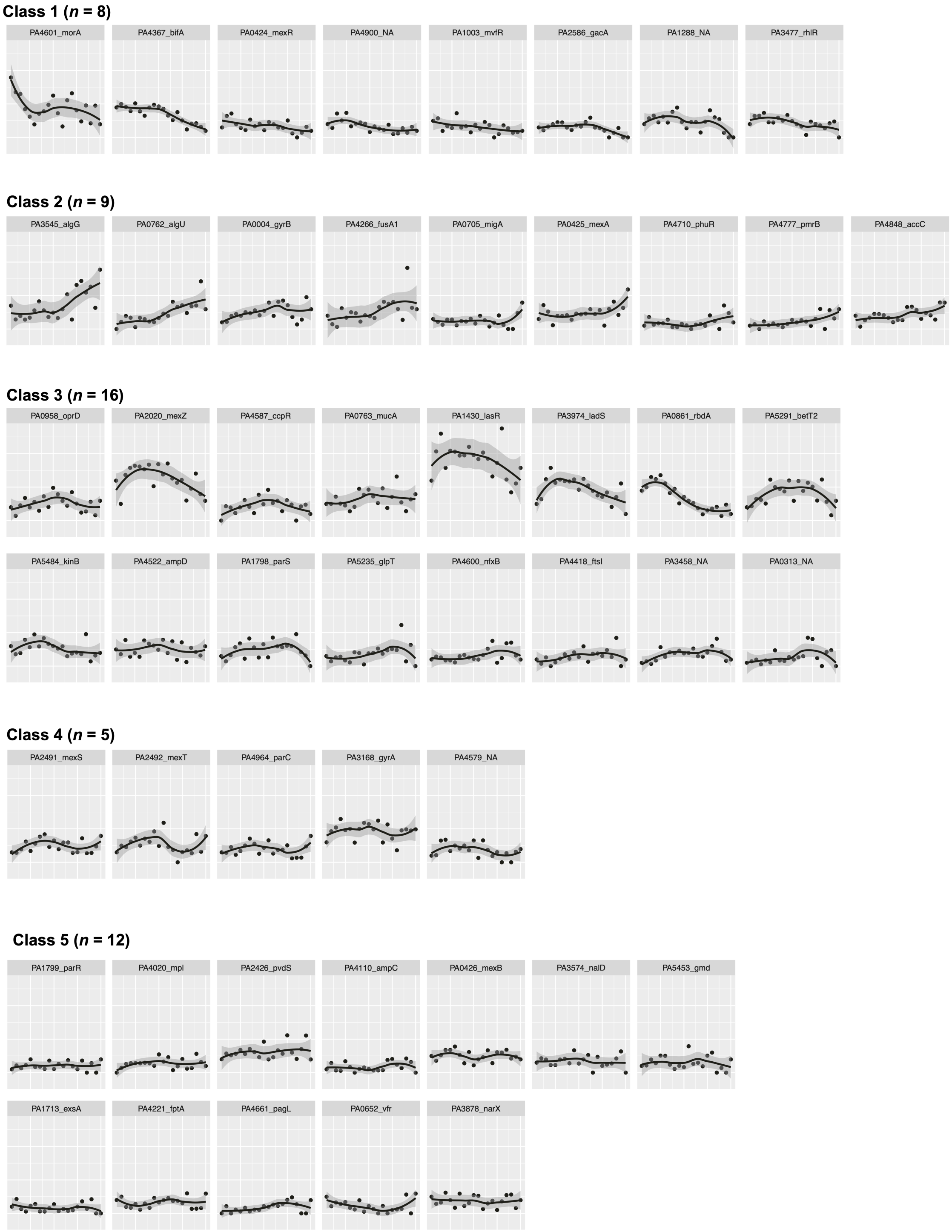

Figure S15. Frequency of mutations over evolutionary time in pathoadaptive genes. Mutation frequencies were position-normalised and the trajectories of the 50 most frequently mutated genes were manually assigned to one of five classes of genes with similar frequency curve shapes. Trendlines from locally weighted smoothing are shown. X axis shows evolutionary time.

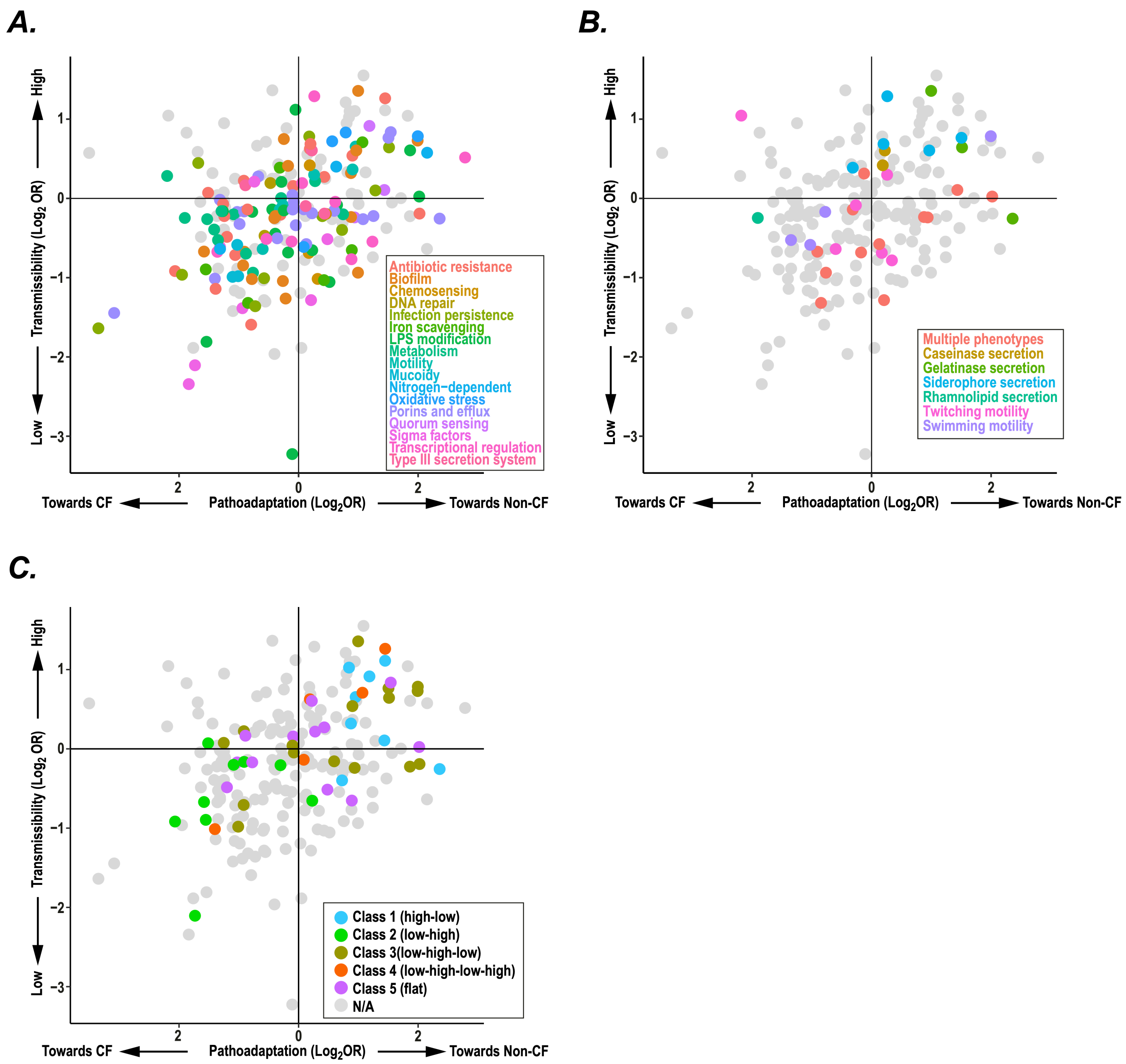

Figure S16. The relative transmissibility and host-specific adaptation of pathoadaptive genes. To estimate host-specific pathoadaptation, the number of cystic fibrosis (CF) vs non-CF mutations (determined by stratifying mutations in pathoadaptive genes on terminal branches by the infection type of isolates) were compared using a Fisher exact test (FDR = 0.1) and expressed as an odds ratio. To assess the transmissibility of pathoadaptation, the number of mutations that had been observed in at least two isolates were compared with mutations that had only been observed once using a Fisher exact test (FDR = 0.1). Genes were colour-coded (A) by established function, (B) based on experimentally-derived functions based on our analyses of corresponding transposon mutants, or (C) by the class of mutation frequency change over evolutionary time.

References

1. J. L. Whitby, A. Rampling, PSEUDOMONAS ÆRUGINOSA CONTAMINATION IN DOMESTIC AND HOSPITAL ENVIRONMENTS. *Lancet* **299**, 15–17 (1972).

2. S. K. Green, M. N. Schroth, J. J. Cho, S. K. Kominos, V. B. Vitanza-jack, Agricultural plants and soil as a reservoir for Pseudomonas aeruginosa. *Appl. Microbiol.* **28**, 987–991 (1974).

3. J.-P. Pirnay, S. Matthijs, H. Colak, P. Chablain, F. Bilocq, J. Van Eldere, D. De Vos, M. Zizi, L. Triest, P. Cornelis, Global Pseudomonas aeruginosa biodiversity as reflected in a Belgian river. *Environ. Microbiol.* **7**, 969–980 (2005).

4. L. M. Ringen, C. H. Drake, A study of the incidence of Pseudomonas aeruginosa from various natural sources. *J. Bacteriol.* **64**, 841–845 (1952).

5. S. Crone, M. Vives-Flórez, L. Kvich, A. M. Saunders, M. Malone, M. H. Nicolaisen, E. Martínez-García, C. Rojas-Acosta, M. Catalina Gomez-Puerto, H. Calum, M. Whiteley, R. Kolter, T. Bjarnsholt, The environmental occurrence of Pseudomonas aeruginosa. *APMIS* **128**, 220–231 (2020).

6. ECDC, "Healthcare-associated infections acquired in intensive care units. Annual epidemiological report for 2017" (European Centre for Disease Prevention and Control., 2019).

7. T. F. Murphy, A. L. Brauer, K. Eschberger, P. Lobbins, L. Grove, X. Cai, S. Sethi, Pseudomonas aeruginosa in chronic obstructive pulmonary disease. *Am. J. Respir. Crit. Care Med.* **177**, 853–860 (2008).

8. S. Rajan, L. Saiman, Pulmonary infections in patients with cystic fibrosis. *Semin. Respir. Infect.* **17**, 47–56 (2002).

9. S. Finch, M. J. McDonnell, H. Abo-Leyah, S. Aliberti, J. D. Chalmers, A Comprehensive Analysis of the Impact of Pseudomonas aeruginosa Colonization on Prognosis in Adult Bronchiectasis. *Ann. Am. Thorac. Soc.* **12**, 1602–1611 (2015).

10. M. R. Kosorok, L. Zeng, S. E. West, M. J. Rock, M. L. Splaingard, A. Laxova, C. G. Green, J. Collins, P. M. Farrell, Acceleration of lung disease in children with cystic fibrosis after Pseudomonas aeruginosa acquisition. *Pediatr. Pulmonol.* **32**, 277–287 (2001).

11. E. Tacconelli, E. Carrara, A. Savoldi, S. Harbarth, M. Mendelson, D. L. Monnet, C. Pulcini, G. Kahlmeter, J. Kluytmans, Y. Carmeli, M. Ouellette, K. Outterson, J. Patel, M. Cavaleri, E. M. Cox, C. R. Houchens, M. L. Grayson, P. Hansen, N. Singh, U. Theuretzbacher, N. Magrini, WHO Pathogens Priority List Working Group, Discovery, research, and development of new antibiotics: the WHO priority list of antibiotic-resistant bacteria and tuberculosis. *Lancet Infect. Dis.* **18**, 318–327 (2018).

12. K. S. Ikuta, L. R. Swetschinski, G. Robles Aguilar, F. Sharara, T. Mestrovic, A. P. Gray, N. Davis Weaver, E. E. Wool, C. Han, A. Gershberg Hayoon, A. Aali, S. M. Abate, M. Abbasi-Kangevari, Z. Abbasi-Kangevari, S. Abd-Elsalam, G. Abebe, A. Abedi, A. P. Abhari, H. Abidi, R. G. Aboagye, A. Absalan, H. Abubaker Ali, J. M. Acuna, T. D. Adane, I. Y. Addo, O. A. Adegboye, M. Adnan, Q. E. S. Adnani, M. S. Afzal, S. Afzal, Z. B. Aghdam, B. O. Ahinkorah,

A. Ahmad, A. R. Ahmad, R. Ahmad, S. Ahmad, S. Ahmad, S. Ahmadi, A. Ahmed, H. Ahmed, J. Q. Ahmed, T. Ahmed Rashid, M. Ajami, B. Aji, M. Akbarzadeh-Khiavi, C. J. Akunna, H. Al Hamad, F. Alahdab, Z. Al-Aly, M. A. Aldeyab, A. V. Aleman, F. A. N. Alhalaiqa, R. K. Alhassan, B. A. Ali, L. Ali, S. S. Ali, Y. Alimohamadi, V. Alipour, A. Alizadeh, S. M. Aljunid, K. Allel, S. Almustanyir, E. K. Ameyaw, A. M. L. Amit, N. Anandavelane, R. Ancuceanu, C. L. Andrei, T. Andrei, D. Anggraini, A. Ansar, A. E. Anyasodor, J. Arabloo, A. Y. Aravkin, D. Areda, T. Aripov, A. A. Artamonov, J. Arulappan, R. T. Aruleba, M. Asaduzzaman, T. Ashraf, S. S. Athari, D. Atlaw, S. Attia, M. Ausloos, T. Awoke, B. P. Ayala Quintanilla, T. M. Ayana, S. Azadnajafabad, A. Azari Jafari, B, Darshan B, M. Badar, A. D. Badiye, N. Baghcheghi, S. Bagherieh, A. A. Baig, I. Banerjee, A. Barac, M. Bardhan, F. Barone-Adesi, H. J. Barqawi, A. Barrow, P. Baskaran, S. Basu, A.-M. M. Batiha, N. Bedi, M. A. Belete, U. I. Belgaumi, R. G. Bender, B. Bhandari, D. Bhandari, P. Bhardwaj, S. Bhaskar, K. Bhattacharyya, S. Bhattarai, S. Bitaraf, D. Buonsenso, Z. A. Butt, F. L. Caetano dos Santos, J. Cai, D. Calina, P. Camargos, L. A. Cámera, R. Cárdenas, M. Cevik, J. Chadwick, J. Charan, A. Chaurasia, P. R. Ching, S. G. Choudhari, E. K. Chowdhury, F. R. Chowdhury, D.-T. Chu, I. S. Chukwu, O. Dadras, F. T. Dagnaw, X. Dai, S. Das, A. Dastiridou, S. A. Debela, F. W. Demisse, S. Demissie, D. Dereje, M. Derese, H. D. Desai, F. N. Dessalegn, S. A. A. Dessalegni, B. Desye, K. Dhaduk, M. Dhimal, S. Dhingra, N. Diao, D. Diaz, S. Djalalinia, M. Dodangeh, D. Dongarwar, B. T. Dora, F. Dorostkar, H. L. Dsouza, E. Dubljanin, S. J. Dunachie, O. C. Durojaiye, H. A. Edinur, H. B. Ejigu, M. Ekholuenetale, T. C. Ekundayo, H. El-Abid, M. Elhadi, M. A. Elmonem, A. Emami, L. Engelbert Bain, D. B. Enyew, R. Erkhembayar, B. Eshrati, F. Etaee, A. F. Fagbamigbe, S. Falahi, A. Fallahzadeh, E. J. A. Faraon, A. Fatehizadeh, G. Fekadu, J. C. Fernandes, A. Ferrari, G. Fetensa, I. Filip, F. Fischer, M. Foroutan, P. A. Gaal, M. A. Gadanya, A. M. Gaidhane, B. Ganesan, M. Gebrehiwot, R. Ghanbari, M. Ghasemi Nour, A. Ghashghaee, A. Gholamrezanezhad, A. Gholizadeh, M. Golechha, P. Goleij, D. Golinelli, A. Goodridge, D. A. Gunawardane, Y. Guo, R. D. Gupta, S. Gupta, V. B. Gupta, V. K. Gupta, A. Guta, P. Habibzadeh, Haddadi Avval, Atlas, R. Halwani, A. Hanif, M. A. Hannan, H. Harapan, S. Hassan, H. Hassankhani, K. Hayat, B. Heibati, G. Heidari, M. Heidari, R. Heidari-Soureshjani, C. Herteliu, D. Z. Heyi, K. Hezam, P. Hoogar, N. Horita, M. M. Hossain, M. Hosseinzadeh, M. Hostiuc, S. Hostiuc, S. Hoveidamanesh, J. Huang, S. Hussain, N. R. Hussein, S. E. Ibitoye, O. S. Ilesanmi, I. M. Ilic, M. D. Ilic, M. T. Imam, M. Immurana, L. R. Inbaraj, A. Iradukunda, N. E. Ismail, C. C. D. Iwu, C. J. Iwu, J, Linda Merin, M. Jakovljevic, E. Jamshidi, T. Javaheri, F. Javanmardi, J. Javidnia, S. K. Jayapal, U. Jayarajah, R. Jebai, R. P. Jha, T. Joo, N. Joseph, F. Joukar, J. J. Jozwiak, S. E. O. Kacimi, V. Kadashetti, L. R. Kalankesh, R. Kalhor, V. K. Kamal, H. Kandel, N. Kapoor, S. Karkhah, B. G. Kassa, N. J. Kassebaum, P. D. Katoto, M. Keykhaei, H. Khajuria, A. Khan, I. A. Khan, M. Khan, M. N. Khan, M. A. B. Khan, M. M. Khatatbeh, M. M. Khater, H. R. Khayat Kashani, J. Khubchandani, H. Kim, M. S. Kim, R. W. Kimokoti, N. Kissoon, S. Kochhar, F. Kompani, S. Kosen, P. A. Koul, S. L. Koulmane Laxminarayana, F. Krapp Lopez, K. Krishan, V. Krishnamoorthy, V. Kulkarni, N. Kumar, O. P. Kurmi, A. Kuttikkattu, H. H. Kyu, D. K. Lal, J. Lám, I. Landires, S. Lasrado, S.-W. Lee, J. Lenzi, S. Lewycka, S. Li, S. S. Lim, W. Liu, R. Lodha, M. J. Loftus, A. Lohiya, L. Lorenzovici, M. Lotfi, A. Mahmoodpoor, M. A. Mahmoud, R. Mahmoudi, A. Majeed, J. Majidpoor, A. Makki, G. A. Mamo, Y. Manla, M. Martorell, C. N. Matei, B. McManigal, E. Mehrabi Nasab, R. Mehrotra, A. Melese, O. Mendoza-Cano, R. G. Menezes, A.-F. A. Mentis, G. Micha, I. M. Michalek, A. C. Micheletti Gomide Nogueira de Sá, N. Milevska Kostova, S. A. Mir, M. Mirghafourvand, S. Mirmoeeni, E. M. Mirrakhimov, M. Mirza-Aghazadeh-Attari, A. S. Misganaw, A. Misganaw, S. Misra, E. Mohammadi, M. Mohammadi, A. Mohammadian-Hafshejani, S. Mohammed, S. Mohan, M. Mohseni, A. H. Mokdad, S. Momtazmanesh, L. Monasta, C. E. Moore, M. Moradi, M. Moradi Sarabi, S. D. Morrison, M. Motaghinejad, H. Mousavi Isfahani, A. Mousavi Khaneghah, S. A. Mousavi-Aghdas, S. Mubarik, F. Mulita, G. B. B. Mulu, S. B. Munro, S. Muthupandian, T. S. Nair, A.

A. Naqvi, H. Narang, Z. S. Natto, M. Naveed, B. P. Nayak, S. Naz, I. Negoi, S. A. Nejadghaderi, S. Neupane Kandel, C. H. Ngwa, R. K. Niazi, A. T. Nogueira de Sá, N. Noroozi, H. Nouraei, A. Nowroozi, V. Nuñez-Samudio, J. J. Nutor, C. I. Nzoputam, O. J. Nzoputam, B. Oancea, R. Obaidur, V. A. Ojha, A. P. Okekunle, O. C. Okonji, A. T. Olagunju, B. O. Olusanya, A. Omar Bali, E. Omer, N. Otstavnov, B. Oumer, M. P A, J. R. Padubidri, K. Pakshir, T. Palicz, A. Pana, S. Pardhan, J. L. Paredes, U. Parekh, E.-C. Park, S. Park, A. Pathak, R. Paudel, U. Paudel, S. Pawar, H. Pazoki Toroudi, M. Peng, U. Pensato, V. C. F. Pepito, M. Pereira, M. F. P. Peres, N. Perico, I.-R. Petcu, Z. Z. Piracha, I. Podder, N. Pokhrel, R. Poluru, M. J. Postma, N. Pourtaheri, A. Prashant, I. Qattea, M. Rabiee, N. Rabiee, A. Radfar, S. Raeghi, S. Rafiei, P. R. Raghav, L. Rahbarnia, V. Rahimi-Movaghar, M. Rahman, M. A. Rahman, A. M. Rahmani, V. Rahmanian, P. Ram, M. M. A. N. Ranjha, S. J. Rao, M.-M. Rashidi, A. Rasul, Z. A. Ratan, S. Rawaf, R. Rawassizadeh, M. S. Razeghinia, E. M. M. Redwan, M. T. Regasa, G. Remuzzi, M. A. Reta, N. Rezaei, A. Rezapour, A. Riad, R. K. Ripon, K. E. Rudd, B. Saddik, S. Sadeghian, U. Saeed, M. Safaei, A. Safary, S. Z. Safi, M. Sahebazzamani, A. Sahebkar, H. Sahoo, S. Salahi, S. Salahi, H. Salari, S. Salehi, H. Samadi Kafil, A. M. Samy, N. Sanadgol, S. Sankararaman, F. Sanmarchi, B. Sathian, M. Sawhney, G. K. Saya, S. Senthilkumaran, A. Seylani, P. A. Shah, M. A. Shaikh, E. Shaker, M. Z. Shakhmardanov, M. M. Sharew, A. Sharifi-Razavi, P. Sharma, R. A. Sheikhi, A. Sheikhy, P. H. Shetty, M. Shigematsu, J. I. Shin, H. Shirzad-Aski, K. M. Shivakumar, P. Shobeiri, S. A. Shorofi, S. Shrestha, M. M. Sibhat, N. B. Sidemo, M. K. Sikder, L. M. L. R. Silva, J. A. Singh, P. Singh, S. Singh, M. S. Siraj, S. S. Siwal, V. Y. Skryabin, A. A. Skryabina, B. Socea, D. D. Solomon, Y. Song, C. T. Sreeramareddy, M. Suleman, R. Suliankatchi Abdulkader, S. Sultana, M. Szócska, S.-A. Tabatabaeizadeh, M. Tabish, M. Taheri, E. Taki, K.-K. Tan, S. Tandukar, N. Y. Tat, V. Y. Tat, B. N. Tefera, Y. M. Tefera, G. Temesgen, M.-H. Temsah, S. Tharwat, A. Thiyagarajan, I. I. Tleyjeh, C. E. Troeger, K. K. Umapathi, E. Upadhyay, S. Valadan Tahbaz, P. R. Valdez, J. Van den Eynde, H. R. van Doorn, S. Vaziri, G.-I. Verras, H. Viswanathan, B. Vo, A. Waris, G. T. Wassie, N. D. Wickramasinghe, S. Yaghoubi, G. A. T. Y. Yahya, S. H. Yahyazadeh Jabbari, A. Yigit, V. Yiğit, D. K. Yon, N. Yonemoto, M. Zahir, B. A. Zaman, S. B. Zaman, M. Zangiabadian, I. Zare, M. S. Zastrozhin, Z.-J. Zhang, P. Zheng, C. Zhong, M. Zoladl, A. Zumla, S. I. Hay, C. Dolecek, B. Sartorius, C. J. L. Murray, M. Naghavi, Global mortality associated with 33 bacterial pathogens in 2019: a systematic analysis for the Global Burden of Disease Study 2019. *Lancet* **400**, 2221–2248 (2022).

13. P. J. Mitchelmore, J. Randall, M. J. Bull, K. A. Moore, P. A. O'Neill, K. Paszkiewicz, E. Mahenthiralingam, C. J. Scotton, C. D. Sheldon, N. J. Withers, A. R. Brown, Molecular epidemiology of Pseudomonas aeruginosa in an unsegregated bronchiectasis cohort sharing hospital facilities with a cystic fibrosis cohort. *Thorax*, doi: 10.1136/thoraxjnl-2016-209889 (2017).

14. P. J. Stapleton, C. Izydorcyzk, S. Clark, A. Blanchard, P. W. Wang, Y. Yau, V. Waters, D. S. Guttman, Pseudomonas aeruginosa strain sharing in early infection among children with cystic fibrosis. *Clin. Infect. Dis.*, doi: 10.1093/cid/ciaa788 (2020).

15. G. A. Tramper-Stranders, C. K. van der Ent, T. F. W. Wolfs, J. L. L. Kimpen, A. Fleer, U. Johansen, H. K. Johansen, N. Høiby, Pseudomonas aeruginosa diversity in distinct paediatric patient groups. *Clin. Microbiol. Infect.* **14**, 935–941 (2008).

16. T. E. Woo, R. Lim, M. G. Surette, B. Waddell, J. C. Bowron, R. Somayaji, J. Duong, C. H. Mody, H. R. Rabin, D. G. Storey, M. D. Parkins, Epidemiology and natural history of Pseudomonas aeruginosa airway infections in non-cystic fibrosis bronchiectasis. *ERJ Open Res* **4** (2018).

17. A. AbdulWahab, S. J. Taj-Aldeen, E. Ibrahim, S. H. Abdulla, R. Muhammed, I. Ahmed, Y. Abdeen, O. Sadek, M. Abu-Madi, Genetic relatedness and host specificity of Pseudomonas aeruginosa isolates from cystic fibrosis and non-cystic fibrosis patients. *Infect. Drug Resist.* **7**, 309–316 (2014).

18. V. N. Kos, M. Déraspe, R. E. McLaughlin, J. D. Whiteaker, P. H. Roy, R. A. Alm, J. Corbeil, H. Gardner, The resistome of Pseudomonas aeruginosa in relationship to phenotypic susceptibility. *Antimicrob. Agents Chemother.* **59**, 427–436 (2015).

19. M. P. Moore, I. L. Lamont, D. Williams, S. Paterson, I. Kukavica-Ibrulj, N. P. Tucker, D. T. D. Kenna, J. F. Turton, J. Jeukens, L. Freschi, B. A. Wee, N. J. Loman, S. Holden, S. Manzoor, P. Hawkey, K. W. Southern, M. J. Walshaw, R. C. Levesque, J. L. Fothergill, C. Winstanley, Transmission, adaptation and geographical spread of the Pseudomonas aeruginosa Liverpool epidemic strain. *Microb Genom* **7** (2021).

20. C. López-Causapé, L. M. Sommer, G. Cabot, R. Rubio, A. A. Ocampo-Sosa, H. K. Johansen, J. Figuerola, R. Cantón, T. J. Kidd, S. Molin, A. Oliver, Evolution of the Pseudomonas aeruginosa mutational resistome in an international Cystic Fibrosis clone. *Sci. Rep.* **7**, 5555 (2017).

21. L. Freschi, A. T. Vincent, J. Jeukens, J.-G. Emond-Rheault, I. Kukavica-Ibrulj, M.-J. Dupont, S. J. Charette, B. Boyle, R. C. Levesque, The Pseudomonas aeruginosa pan-genome provides new insights on its population structure, horizontal gene transfer and pathogenicity. *Genome Biol. Evol.*, doi: 10.1093/gbe/evy259 (2018).

22. Y. Hilliam, M. P. Moore, I. L. Lamont, D. Bilton, C. S. Haworth, J. Foweraker, M. J. Walshaw, D. Williams, J. L. Fothergill, A. De Soyza, C. Winstanley, Pseudomonas aeruginosa adaptation and diversification in the non-cystic fibrosis bronchiectasis lung. *Eur. Respir. J.* **49** (2017).

23. J. Chilam, S. Argimón, M. T. Limas, M. L. Masim, J. M. Gayeta, M. L. Lagrada, A. M. Olorosa, V. Cohen, L. T. Hernandez, B. Jeffrey, K. Abudahab, C. M. Hufano, S. B. Sia, M. T. G. Holden, J. Stelling, D. M. Aanensen, C. C. Carlos, Philippines Antimicrobial Resistance Surveillance Program, Genomic surveillance of Pseudomonas aeruginosa in the Philippines, 2013-2014. *Western Pac Surveill Response J* **12**, 4–18 (2021).

24. E. Del Barrio-Tofiño, C. López-Causapé, G. Cabot, A. Rivera, N. Benito, C. Segura, M. M. Montero, L. Sorlí, F. Tubau, S. Gómez-Zorrilla, N. Tormo, R. Durá-Navarro, E. Viedma, E. Resino-Foz, M. Fernández-Martínez, C. González-Rico, I. Alejo-Cancho, J. A. Martínez, C. Labayru-Echverria, C. Dueñas, I. Ayestarán, L. Zamorano, L. Martinez-Martinez, J. P. Horcajada, A. Oliver, Genomics and Susceptibility Profiles of Extensively Drug-Resistant Pseudomonas aeruginosa Isolates from Spain. *Antimicrob. Agents Chemother.* **61**, AAC.01589-17 (2017).

25. A. Khaledi, A. Weimann, M. Schniederjans, E. Asgari, T.-H. Kuo, A. Oliver, G. Cabot, A. Kola, P. Gastmeier, M. Hogardt, D. Jonas, M. R. Mofrad, A. Bremges, A. C. McHardy, S. Häussler, Predicting antimicrobial resistance in Pseudomonas aeruginosa with machine learning-enabled molecular diagnostics. *EMBO Mol. Med.* **12**, e10264 (2020).

26. R. L. Marvig, L. M. Sommer, S. Molin, H. K. Johansen, Convergent evolution and adaptation of Pseudomonas aeruginosa within patients with cystic fibrosis. *Nat. Genet.* **47**, 57–64 (2015).

27. J. F. Turton, L. Wright, A. Underwood, A. A. Witney, Y.-T. Chan, A. Al-Shahib, C. Arnold, M. Doumith, B. Patel, T. D. Planche, J. Green, R. Holliman, N. Woodford, High-Resolution Analysis by Whole-Genome Sequencing of an International Lineage (Sequence Type 111) of Pseudomonas aeruginosa Associated with Metallo-Carbapenemases in the United Kingdom. *J. Clin. Microbiol.* **53**, 2622–2631 (2015).

28. B. Curran, D. Jonas, H. Grundmann, T. Pitt, C. G. Dowson, Development of a multilocus sequence typing scheme for the opportunistic pathogen Pseudomonas aeruginosa. *J. Clin. Microbiol.* **42**, 5644–5649 (2004).

29. R. Bouckaert, J. Heled, D. Kühnert, T. Vaughan, C.-H. Wu, D. Xie, M. A. Suchard, A. Rambaut, A. J. Drummond, BEAST 2: a software platform for Bayesian evolutionary analysis. *PLoS Comput. Biol.* **10**, e1003537 (2014).

30. A. J. Drummond, A. Rambaut, B. Shapiro, O. G. Pybus, Bayesian coalescent inference of past population dynamics from molecular sequences. *Mol. Biol. Evol.* **22**, 1185–1192 (2005).

31. W. H. McNeill, Human Migration in Historical Perspective. *Popul. Dev. Rev.* **10**, 1–18 (1984).

32. A. P. Dobson, E. R. Carper, Infectious Diseases and Human Population History. *Bioscience* **46**, 115–126 (1996).

33. F. J. Kelly, J. C. Fussell, Air pollution and airway disease. *Clin. Exp. Allergy* **41**, 1059–1071 (2011).

34. A. J. Chauhan, S. L. Johnston, Air pollution and infection in respiratory illness. *Br. Med. Bull.* **68**, 95–112 (2003).

35. M. Kampa, E. Castanas, Human health effects of air pollution. *Environ. Pollut.* **151**, 362–367 (2008).

36. P. Lemey, A. Rambaut, A. J. Drummond, M. A. Suchard, Bayesian phylogeography finds its roots. *PLoS Comput. Biol.* **5**, e1000520 (2009).

37. M. I. Katsnelson, Y. I. Wolf, E. V. Koonin, On the feasibility of saltational evolution. *Proc. Natl. Acad. Sci. U. S. A.* **116**, 21068–21075 (2019).

38. J. M. Bryant, K. P. Brown, S. Burbaud, I. Everall, J. M. Belardinelli, D. Rodriguez-Rincon, D. M. Grogono, C. M. Peterson, D. Verma, I. E. Evans, C. Ruis, A. Weimann, D. Arora, S. Malhotra, B. Bannerman, C. Passemar, K. Templeton, G. MacGregor, K. Jiwa, A. J. Fisher, T. L. Blundell, D. J. Ordway, M. Jackson, J. Parkhill, R. A. Floto, Stepwise pathogenic evolution of *Mycobacterium abscessus*. *Science* **372** (2021).

39. G. Tonkin-Hill, N. MacAlasdair, C. Ruis, A. Weimann, G. Horesh, J. A. Lees, R. A. Gladstone, S. Lo, C. Beaudoin, R. A. Floto, S. D. W. Frost, J. Corander, S. D. Bentley, J. Parkhill, Producing polished prokaryotic pangenomes with the Panaroo pipeline. *Genome Biol.* **21**, 180 (2020).

40. K. Martin, B. Baddal, N. Mustafa, C. Perry, A. Underwood, C. Constantidou, N. Loman, D. T. Kenna, J. F. Turton, Clusters of genetically similar isolates of Pseudomonas aeruginosa from multiple hospitals in the UK. *J. Med. Microbiol.* **62**, 988–1000 (2013).

41. J. Day, A. Friedman, L. S. Schlesinger, Modeling the immune rheostat of macrophages in the lung in response to infection. *Proc. Natl. Acad. Sci. U. S. A.* **106**, 11246–11251 (2009).

42. A. Craig, J. Mai, S. Cai, S. Jeyaseelan, Neutrophil recruitment to the lungs during bacterial pneumonia. *Infect. Immun.* **77**, 568–575 (2009).

43. A. Fortuna, D. Collalto, V. Schiaffi, V. Pastore, P. Visca, F. Ascenzioni, G. Rampioni, L. Leoni, The Pseudomonas aeruginosa DksA1 protein is involved in H2O2 tolerance and within-macrophages survival and can be replaced by DksA2. *Sci. Rep.* **12**, 10404 (2022).

44. A. Fortuna, H. Bähre, P. Visca, G. Rampioni, L. Leoni, The two Pseudomonas aeruginosa DksA stringent response proteins are largely interchangeable at the whole transcriptome level and in the control of virulence-related traits. *Environ. Microbiol.* **23**, 5487–5504 (2021).

45. K. B. Turton, R. J. Ingram, M. A. Valvano, Macrophage dysfunction in cystic fibrosis: Nature or nurture? *J. Leukoc. Biol.* **109**, 573–582 (2021).

46. A. Bernut, C. A. Loynes, R. A. Floto, S. A. Renshaw, Deletion of cftr Leads to an Excessive Neutrophilic Response and Defective Tissue Repair in a Zebrafish Model of Sterile Inflammation. *Front. Immunol.* **11** (2020).

47. A. Bernut, J.-L. Herrmann, K. Kissa, J.-F. Dubremetz, J.-L. Gaillard, G. Lutfalla, L. Kremer, Mycobacterium abscessus cording prevents phagocytosis and promotes abscess formation. *Proc. Natl. Acad. Sci. U. S. A.* **111**, E943-52 (2014).

48. P. Cingolani, A. Platts, L. L. Wang, M. Coon, T. Nguyen, L. Wang, S. J. Land, X. Lu, D. M. Ruden, A program for annotating and predicting the effects of single nucleotide polymorphisms, SnpEff: SNPs in the genome of Drosophila melanogaster strain w1118; iso-2; iso-3. *Fly* **6**, 80–92 (2012).

49. R. Vaser, S. Adusumalli, S. N. Leng, M. Sikic, P. C. Ng, SIFT missense predictions for genomes. *Nat. Protoc.* **11**, 1–9 (2016).

50. J. Delgado, L. G. Radusky, D. Cianferoni, L. Serrano, FoldX 5.0: working with RNA, small molecules and a new graphical interface. *Bioinformatics* **35**, 4168–4169 (2019).

51. S. Westbrock-Wadman, D. R. Sherman, M. J. Hickey, S. N. Coulter, Y. Q. Zhu, P. Warrener, L. Y. Nguyen, R. M. Shawar, K. R. Folger, C. K. Stover, Characterization of a Pseudomonas aeruginosa efflux pump contributing to aminoglycoside impermeability. *Antimicrob. Agents Chemother.* **43**, 2975–2983 (1999).

52. K. Poole, K. Tetro, Q. Zhao, S. Neshat, D. E. Heinrichs, N. Bianco, Expression of the multidrug resistance operon mexA-mexB-oprM in Pseudomonas aeruginosa: mexR encodes a regulator of operon expression. *Antimicrob. Agents Chemother.* **40**, 2021–2028 (1996).

53. L. Rust, E. C. Pesci, B. H. Iglewski, Analysis of the Pseudomonas aeruginosa elastase (lasB) regulatory region. *J. Bacteriol.* **178**, 1134–1140 (1996).

54. T. L. Yahr, D. W. Frank, Transcriptional organization of the trans-regulatory locus which controls exoenzyme S synthesis in Pseudomonas aeruginosa. *J. Bacteriol.* **176**, 3832–3838 (1994).

55. G. L. Winsor, E. J. Griffiths, R. Lo, B. K. Dhillon, J. A. Shay, F. S. L. Brinkman, Enhanced annotations and features for comparing thousands of Pseudomonas genomes in the Pseudomonas genome database. *Nucleic Acids Res.* **44**, D646-53 (2016).

56. D. Szklarczyk, A. L. Gable, D. Lyon, A. Junge, S. Wyder, J. Huerta-Cepas, M. Simonovic, N. T. Doncheva, J. H. Morris, P. Bork, L. J. Jensen, C. von Mering, STRING v11: protein–protein association networks with increased coverage, supporting functional discovery in genome-wide experimental datasets. *Nucleic Acids Res.* **47**, D607–D613 (2018).

57. A. Alexa, J. Rahnenführer, T. Lengauer, Improved scoring of functional groups from gene expression data by decorrelating GO graph structure. *Bioinformatics* **22**, 1600–1607 (2006).

58. D. W. Martin, M. J. Schurr, H. Yu, V. Deretic, Analysis of promoters controlled by the putative sigma factor AlgU regulating conversion to mucoidy in Pseudomonas aeruginosa: relationship to sigma E and stress response. *J. Bacteriol.* **176**, 6688–6696 (1994).

59. U. N. Broder, T. Jaeger, U. Jenal, LadS is a calcium-responsive kinase that induces acute-to-chronic virulence switch in Pseudomonas aeruginosa. *Nat Microbiol* **2**, 16184 (2016).

60. C. Chen, G. A. Beattie, Pseudomonas syringae BetT is a low-affinity choline transporter that is responsible for superior osmoprotection by choline over glycine betaine. *J. Bacteriol.* **190**, 2717–2725 (2008).

61. I. Santi, P. Manfredi, E. Maffei, A. Egli, U. Jenal, Evolution of Antibiotic Tolerance Shapes Resistance Development in Chronic Pseudomonas aeruginosa Infections. *MBio* **12** (2021).

62. R. Reynolds, R. Hope, L. Williams, BSAC Working Parties on Resistance Surveillance, Survey, laboratory and statistical methods for the BSAC Resistance Surveillance Programmes. *J. Antimicrob. Chemother.* **62 Suppl 2**, ii15-28 (2008).

63. H. Li, Aligning sequence reads, clone sequences and assembly contigs with BWA-MEM, *arXiv [q-bio.GN]* (2013). http://arxiv.org/abs/1303.3997.

64. M. Hunt, A. E. Mather, L. Sánchez-Busó, A. J. Page, J. Parkhill, J. A. Keane, S. R. Harris, ARIBA: rapid antimicrobial resistance genotyping directly from sequencing reads. *Microb Genom* **3**, e000131 (2017).

65. M. N. Price, P. S. Dehal, A. P. Arkin, FastTree 2--approximately maximum-likelihood trees for large alignments. *PLoS One* **5**, e9490 (2010).

66. A. J. Page, B. Taylor, A. J. Delaney, J. Soares, T. Seemann, J. A. Keane, S. R. Harris, SNP-sites: rapid efficient extraction of SNPs from multi-FASTA alignments. *Microb Genom* **2**, e000056 (2016).

67. N. J. Croucher, A. J. Page, T. R. Connor, A. J. Delaney, J. A. Keane, S. D. Bentley, J. Parkhill, S. R. Harris, Rapid phylogenetic analysis of large samples of recombinant bacterial whole genome sequences using Gubbins. *Nucleic Acids Res.* **43**, e15 (2015).

68. A. Rambaut, T. T. Lam, L. Max Carvalho, O. G. Pybus, Exploring the temporal structure of heterochronous sequences using TempEst (formerly Path-O-Gen). *Virus Evol* **2**, vew007 (2016).

69. A. Stamatakis, RAxML version 8: a tool for phylogenetic analysis and post-analysis of large phylogenies. *Bioinformatics* **30**, 1312–1313 (2014).

70. A. Rambaut, A. J. Drummond, D. Xie, G. Baele, M. A. Suchard, Posterior Summarization in Bayesian Phylogenetics Using Tracer 1.7. *Syst. Biol.* **67**, 901–904 (2018).

71. F. Menardo, S. Duchêne, D. Brites, S. Gagneux, The molecular clock of Mycobacterium tuberculosis. *PLoS Pathog.* **15**, e1008067 (2019).

72. J. Parker, A. Rambaut, O. G. Pybus, Correlating viral phenotypes with phylogeny: accounting for phylogenetic uncertainty. *Infect. Genet. Evol.* **8**, 239–246 (2008).

73. F. Bielejec, A. Rambaut, M. A. Suchard, P. Lemey, SPREAD: spatial phylogenetic reconstruction of evolutionary dynamics. *Bioinformatics* **27**, 2910–2912 (2011).

74. J. Huerta-Cepas, K. Forslund, L. P. Coelho, D. Szklarczyk, L. J. Jensen, C. von Mering, P. Bork, Fast Genome-Wide Functional Annotation through Orthology Assignment by eggNOG-Mapper. *Mol. Biol. Evol.* **34**, 2115–2122 (2017).

75. J. M. Bryant, D. M. Grogono, D. Rodriguez-Rincon, I. Everall, K. P. Brown, P. Moreno, D. Verma, E. Hill, J. Drijkoningen, P. Gilligan, C. R. Esther, P. G. Noone, O. Giddings, S. C. Bell, R. Thomson, C. E. Wainwright, C. Coulter, S. Pandey, M. E. Wood, R. E. Stockwell, K. A. Ramsay, L. J. Sherrard, T. J. Kidd, N. Jabbour, G. R. Johnson, L. D. Knibbs, L. Morawska, P. D. Sly, A. Jones, D. Bilton, I. Laurenson, M. Ruddy, S. Bourke, I. C. Bowler, S. J. Chapman, A. Clayton, M. Cullen, T. Daniels, O. Dempsey, M. Denton, M. Desai, R. J. Drew, F. Edenborough, J. Evans, J. Folb, H. Humphrey, B. Isalska, S. Jensen-Fangel, B. Jönsson, A. M. Jones, T. L. Katzenstein, T. Lillebaek, G. MacGregor, S. Mayell, M. Millar, D. Modha, E. F. Nash, C. O'Brien, D. O'Brien, C. Ohri, C. S. Pao, D. Peckham, F. Perrin, A. Perry, T. Pressler, L. Prtak, T. Qvist, A. Robb, H. Rodgers, K. Schaffer, N. Shafi, J. van Ingen, M. Walshaw, D. Watson, N. West, J. Whitehouse, C. S. Haworth, S. R. Harris, D. Ordway, J. Parkhill, R. A. Floto, Emergence and spread of a human-transmissible multidrug-resistant nontuberculous mycobacterium. *Science* **354**, 751–757 (2016).

76. T. Mäklin, T. Kallonen, S. David, C. J. Boinett, B. Pascoe, G. Méric, D. M. Aanensen, E. J. Feil, S. Baker, J. Parkhill, S. K. Sheppard, J. Corander, A. Honkela, High-resolution sweep metagenomics using fast probabilistic inference. *Wellcome Open Res* **5**, 14 (2020).

77. N. L. Bray, H. Pimentel, P. Melsted, L. Pachter, Near-optimal probabilistic RNA-seq quantification. *Nat. Biotechnol.* **34**, 525–527 (2016).

78. M. Love, S. Anders, W. Huber, Differential analysis of count data--the DESeq2 package. *Genome Biol.* **15**, 550 (2014).

79. A. Bernut, C. Dupont, N. V. Ogryzko, A. Neyret, J.-L. Herrmann, R. A. Floto, S. A. Renshaw, L. Kremer, CFTR Protects against Mycobacterium abscessus Infection by Fine-Tuning Host Oxidative Defenses. *Cell Rep.* **26**, 1828-1840.e4 (2019).

80. C. Belon, C. Soscia, A. Bernut, A. Laubier, S. Bleves, A.-B. Blanc-Potard, A Macrophage Subversion Factor Is Shared by Intracellular and Extracellular Pathogens. *PLoS Pathog.* **11**, e1004969 (2015).

81. V. Le Moigne, A.-L. Roux, H. Mahoudo, G. Christien, A. Ferroni, O. Dumitrescu, G. Lina, J.-P. Bouchara, P. Plésiat, J.-L. Gaillard, S. Canaan, G. Héry-Arnaud, J.-L. Herrmann, Serological biomarkers for the diagnosis of Mycobacterium abscessus infections in cystic fibrosis patients. *J. Cyst. Fibros.* **21**, 353–360 (2022).

82. P. Sagulenko, V. Puller, R. A. Neher, TreeTime: Maximum-likelihood phylodynamic analysis. *Virus Evol* **4**, vex042 (2018).

83. A. Weimann, C. Ruis, PhyloEffects, Zenodo (2024); https://doi.org/10.5281/zenodo.10606175.

84. E. F. Pettersen, T. D. Goddard, C. C. Huang, UCSF Chimera—a visualization system for exploratory research and analysis. *Journal of* (2004).

85. B. E. Suzek, H. Huang, P. McGarvey, R. Mazumder, C. H. Wu, UniRef: comprehensive and non-redundant UniProt reference clusters. *Bioinformatics* **23**, 1282–1288 (2007).

86. K. Held, E. Ramage, M. Jacobs, L. Gallagher, C. Manoil, Sequence-verified two-allele transposon mutant library for Pseudomonas aeruginosa PAO1. *J. Bacteriol.* **194**, 6387–6389 (2012).

87. A. Weimann, A. Dinan, Computer codes for Weimann et al - Evolution and host-specific pathoadaptation of Pseudomonas aeruginosa, Zenodo (2024); https://doi.org/10.5281/ZENODO.10625500.

88. A. Weimann, Data for Weimann et al - Evolution and host-specific pathoadaptation of Pseudomonas aeruginosa, Zenodo (2024); https://doi.org/10.5281/ZENODO.10600286.

89. M. Ashburner, C. A. Ball, J. A. Blake, D. Botstein, H. Butler, J. M. Cherry, A. P. Davis, K. Dolinski, S. S. Dwight, J. T. Eppig, M. A. Harris, D. P. Hill, L. Issel-Tarver, A. Kasarskis, S. Lewis, J. C. Matese, J. E. Richardson, M. Ringwald, G. M. Rubin, G. Sherlock, Gene Ontology: tool for the unification of biology. *Nat. Genet.* **25**, 25–29 (2000).

90. A. McKenna, M. Hanna, E. Banks, A. Sivachenko, K. Cibulskis, A. Kernytsky, K. Garimella, D. Altshuler, S. Gabriel, M. Daly, M. A. DePristo, The Genome Analysis Toolkit: a MapReduce framework for analyzing next-generation DNA sequencing data. *Genome Res.* **20**, 1297–1303 (2010).

91. H. Li, A statistical framework for SNP calling, mutation discovery, association mapping and population genetical parameter estimation from sequencing data. *Bioinformatics* **27**, 2987–2993 (2011).

92. P. Danecek, J. K. Bonfield, J. Liddle, J. Marshall, V. Ohan, M. O. Pollard, A. Whitwham, T. Keane, S. A. McCarthy, R. M. Davies, H. Li, Twelve years of SAMtools and BCFtools. *Gigascience* **10** (2021).

93. K. A. Jolley, J. E. Bray, M. C. J. Maiden, Open-access bacterial population genomics: BIGSdb software, the Pubmlst.org website and their applications. *Wellcome Open Res* **3**, 124 (2018).

94. G. Yu, D. K. Smith, H. Zhu, Y. Guan, T. T.-Y. Lam, Ggtree : An r package for visualization and annotation of phylogenetic trees with their covariates and other associated data. *Methods Ecol. Evol.* **8**, 28–36 (2017).

95. A. J. Page, N. De Silva, M. Hunt, M. A. Quail, J. Parkhill, S. R. Harris, T. D. Otto, J. A. Keane, Robust high-throughput prokaryote de novo assembly and improvement pipeline for Illumina data. *Microb Genom* **2**, e000083 (2016).

96. D. R. Zerbino, E. Birney, Velvet: algorithms for de novo short read assembly using de Bruijn graphs. *Genome Res.* **18**, 821–829 (2008).

97. A. Bankevich, S. Nurk, D. Antipov, A. A. Gurevich, M. Dvorkin, A. S. Kulikov, V. M. Lesin, S. I. Nikolenko, S. Pham, A. D. Prjibelski, A. V. Pyshkin, A. V. Sirotkin, N. Vyahhi, G. Tesler, M. A. Alekseyev, P. A. Pevzner, SPAdes: a new genome assembly algorithm and its applications to single-cell sequencing. *J. Comput. Biol.* **19**, 455–477 (2012).

98. P. Shannon, A. Markiel, O. Ozier, N. S. Baliga, J. T. Wang, D. Ramage, N. Amin, B. Schwikowski, T. Ideker, Cytoscape: a software environment for integrated models of biomolecular interaction networks. *Genome Res.* **13**, 2498–2504 (2003).

99. C. P. Cantalapiedra, A. Hernández-Plaza, I. Letunic, P. Bork, J. Huerta-Cepas, eggNOG-mapper v2: Functional Annotation, Orthology Assignments, and Domain Prediction at the Metagenomic Scale. *Mol. Biol. Evol.*, doi: 10.1093/molbev/msab293 (2021).

100. H. C. Valley, K. M. Bukis, A. Bell, Y. Cheng, E. Wong, N. J. Jordan, N. E. Allaire, A. Sivachenko, F. Liang, H. Bihler, P. J. Thomas, J. Mahiou, M. Mense, Isogenic cell models of cystic fibrosis-causing variants in natively expressing pulmonary epithelial cells. *J. Cyst. Fibros.* **18**, 476–483 (2019).

101. D. H. Figurski, D. R. Helinski, Replication of an origin-containing derivative of plasmid RK2 dependent on a plasmid function provided in trans. *Proc. Natl. Acad. Sci. U. S. A.* **76**, 1648–1652 (1979).

102. D. E. Nivens, D. E. Ohman, J. Williams, M. J. Franklin, Role of alginate and its O acetylation in formation of Pseudomonas aeruginosa microcolonies and biofilms. *J. Bacteriol.* **183**, 1047–1057 (2001).

103. Y. Benjamini, Y. Hochberg, Controlling the False Discovery Rate: A Practical and Powerful Approach to Multiple Testing. *J. R. Stat. Soc. Series B Stat. Methodol.* **57**, 289–300 (1995).

104. B. C. Louden, D. Haarmann, A. M. Lynne, Use of Blue Agar CAS Assay for Siderophore Detection. *J. Microbiol. Biol. Educ.* **12**, 51–53 (2011).

105. M. Varadi, S. Anyango, M. Deshpande, S. Nair, C. Natassia, G. Yordanova, D. Yuan, O. Stroe, G. Wood, A. Laydon, A. Žídek, T. Green, K. Tunyasuvunakool, S. Petersen, J. Jumper, E. Clancy, R. Green, A. Vora, M. Lutfi, M. Figurnov, A. Cowie, N. Hobbs, P. Kohli, G. Kleywegt, E. Birney, D. Hassabis, S. Velankar, AlphaFold Protein Structure Database:

massively expanding the structural coverage of protein-sequence space with high-accuracy models. *Nucleic Acids Res.* **50**, D439–D444 (2022).
