# Supplementary material for: Evolution and host-specific adaptation of Pseudomonas aeruginosa
Source: Science. Author manuscript; Available in PMC 2025 Nov 18. (PMC7618370; doi:10.1126/science.adi0908)
Supplement: Fig.2 [file EMS209707-supplement-Fig_2.pdf]

Structured Summary

Introduction

The major human bacterial pathogen, *Pseudomonas aeruginosa,* causes multidrug-resistant infections, particularly in people with underlying immunodeficiencies or inflammatory lung diseases, such as Cystic Fibrosis (CF). However, it remains unclear how *P. aeruginosa* has evolved into a highly adapted, globally disseminated pathogen.

Rationale

We therefore sought to understand the pathogenic evolution of *P. aeruginosa* by combining population-level genomic exploration with transcriptomic and phenotypic analyses.

Results

We analysed a global collection of 9,829 isolates of *P. aeruginosa,* identifying 21 major clones, which we term 'epidemic'. These epidemic clones caused most clinical *P. aeruginosa* infections worldwide, were widely distributed across the phylogenetic tree, and had all spread globally. We estimate that these epidemic clones emerged from ancestral locations distributed around the world, and then expanded non-synchronously between the late 17th and late 20th centuries, potentially driven by changes in human population density, migration patterns, and/or air pollution. Through pan-genome analysis, we identified significant differences between epidemic and sporadic isolate genomes in the acquisition of genes involved in specific cellular processes, such as transcriptional control (***Figure 0***).

We found that epidemic clones appeared to have intrinsic preferences for CF or non-CF individuals and discovered a clear expression signature of genes positively and negatively associated with CF affinity. We found that high CF affinity clones were better able to survive within CF macrophages, in part mediated by expression of the stringent response modulator DksA1, suggesting that enhanced host innate immune evasion might explain the intrinsic success at infecting CF patients of certain epidemic clones (***Figure 0***).

Examining the recent mutation history of individual clones to understand how epidemic clones of *P. aeruginosa* have adapted to the human host through multiple rounds of within-patient evolution by analysing, we identified 224 out of 5641 genes that had a higher total mutational burden than expected by chance, which we term 'pathoadaptive'.

We found that the products of these pathoadaptive genes were tightly interconnected, indicating their likely coordinated functional roles. Many genes were more frequently mutated in either CF or non-CF isolates, suggesting that distinct functional programmes were being modified as part of host-specific adaptation (***Figure 0***).

Pathoadaptive genes were frequently associated with changes in transmissibility and/or host-specific adaptation, thereby potentially driving host specialisation. In support of this notion, we found strong evidence of cross-infection either between CF patients or between non-CF patients, but very little CF to non-CF transmission (***Figure 0***).

Conclusion

Our findings describe the key sequential steps involved in the evolution of *P. aeruginosa* from an environmental organism to a major human pathogen: saltatory evolution caused by horizontal gene transfer generating epidemic clones; varying intrinsic host affinities of these clones (linked to specific transcriptional changes enabling survival within macrophages); and multiple rounds of convergent, host-specific adaptation, eventually resulting in the loss of their ability to transmit between different patient groups. Our work thus highlights the importance of global surveillance and cross-infection prevention in averting the emergence of future epidemic clones.

Caption

Figure 0. Host-specific evolution of *Pseudomonas aeruginosa.* We define key steps in the pathogenic evolution of *P. aeruginosa*: (1) Environmental clones with epidemic potential are created through horizontal gene transfer (revealed through comparative pan-genome graph analysis); (2) Intrinsic, transcriptionally-driven, varying preference of emerging clones for CF hosts, associated with an increased ability to survive within CF macrophages; (3) Distinct trajectories of subsequent evolution of isolates infecting CF versus non-CF patients, mediated by mutations in 224 ‘pathoadaptive’ genes, which influence transmissibility and/or host-specific adaptation; (4) As a result of host specialisation, transmission is constrained between CF and non-CF patients. Top panel created with BioRender.com
